# Insight Into the Nuclear and Mitochondrial Genome of the Caribbean King Crab *Maguimithrax spinosissimus* (Crustacea: Brachyura: Mithracidae) to Support Fisheries Management and Conservation Initiatives

**DOI:** 10.1002/ece3.71619

**Published:** 2025-06-29

**Authors:** Juan Antonio Baeza

**Affiliations:** ^1^ Department of Biological Sciences Clemson University Clemson South Carolina USA; ^2^ Departamento de Biología Marina Universidad Catolica del Norte Coquimbo Chile; ^3^ Smithsonian Marine Station Smithsonian Institution Fort Pierce Florida USA

## Abstract

The Caribbean King crab *Maguimithrax spinosissimus* is the largest brachyuran in the western Atlantic and target of subsistence, recreational, and/or artisanal fisheries. Also, its abbreviated larval period makes it a candidate for mariculture to support coral reef restoration efforts. In this study, I benefitted from a series of bioinformatics tools tailored for low‐sequencing‐depth next generation datasets that allow to gain insight into the genome of a species. K‐mer profiling indicated a haploid genome size that ranged between 2.16 Gbp (k‐mer = 51) and 2.63 Gbp (k‐mer = 21). At least one half and a maximum of three fourths of the nuclear genome comprised mobile elements. Just over one half (57.49%) of the repetitive elements in the nuclear genome of the Caribbean King Crab were not annotated. If only annotated mobile elements are taken into account, the most common were classified as Long Interspersed Nuclear Elements (29.18%). Much less common transposable elements included DNA transposons (5.63%), Long Terminal Repeats (2.69%), and Rolling Circles (1.93%). The mitogenome of the Caribbean King Crab was 15,714 bp long and encoded for 13 protein‐coding genes, 22 transfer RNA genes, and two ribosomal RNA genes. A maximum likelihood phylogenetic analysis based on translated PCGs supported the monophyletic status of the superfamily Majoidea and family Mithracidae but not the Majidae. The results from this study will help adjusting resources optimally to assemble a chromosome‐level nuclear genome in the Caribbean King crab. The assembled mitogenome is expected to support biomonitoring of this species in coral reefs using environmental DNA. Overall, the developed genomic resources can be used to support conservation planning and fisheries regulation in *M. spinosissimus* , the largest crab in the Caribbean basin with potential for aquaculture to support coral reef restoration.

## Graphical abstract

Genomic resources for the Caribbean King Crab.

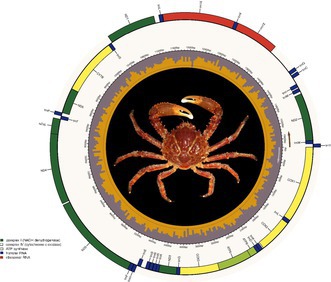

## 1 Introduction

**FIGURE 1:**
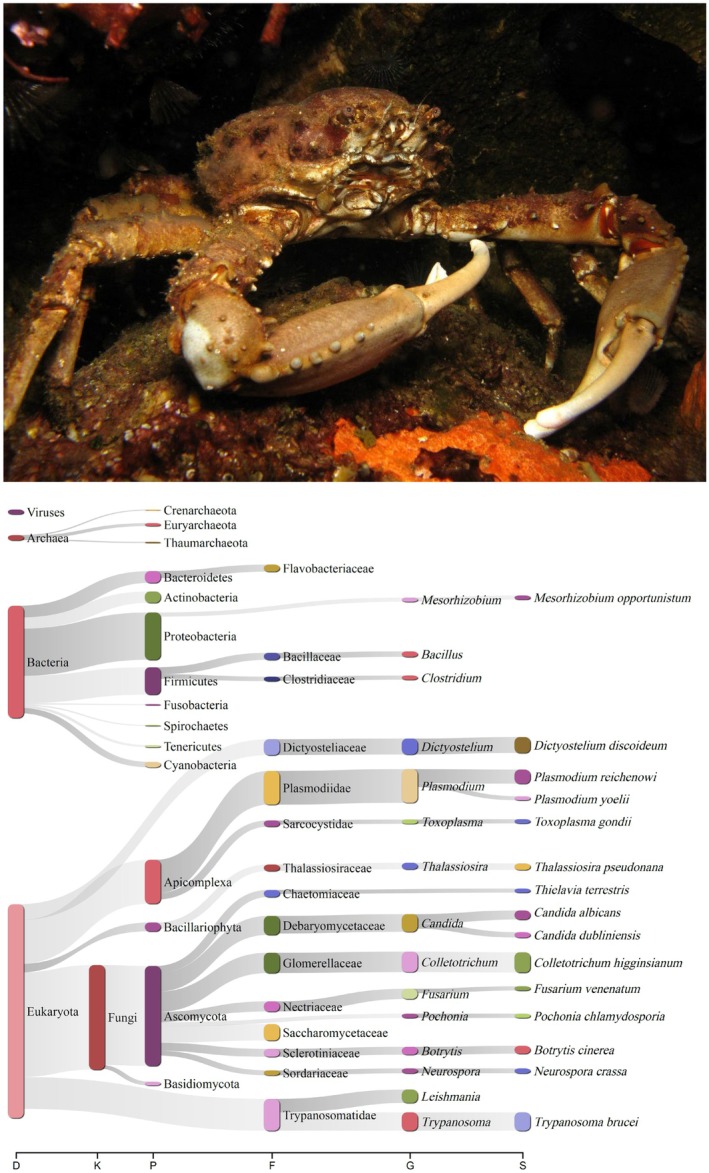
The Caribbean King Crab *Maguimithrax spinosissimus* (top) and sankey diagram generated from the Kraken2 results obtained for *Maguimithrax spinosissimus* (bottom). Photograph credit: J. Antonio Baeza (published with permission).

Among spider crabs belonging to the superfamily Majoidea (*sensu* Ng et al. 2008), one of the most species‐rich clades of brachyurans known for their anatomical, ecological, and behavioral disparity (Martin and Davis 2001; Ng et al. 2008), the Caribbean King Crab, West Indian Spider Crab, or Channel Clinging Crab *Maguimithrax spinosissimus* is of particular interest (Figure 1). The Caribbean King Crab is restricted to the Western Atlantic basin, from South Florida, USA across the Caribbean Islands, to Venezuela (Provenzano and Brownell 1977; Williams 1984). *Maguimithrax spinosissimus* dwells under rocks or in crevices in shallow subtropical and tropical rocky and coral reefs and is often found solitarily within shelters (Williams 1984). The Caribbean King Crab is the largest brachyuran crab in the western Atlantic basin, growing up to ~20 cm carapace length (Rathbun 1925; Williams 1984) and, subsequently, this phyletic giant is the target of subsistence, recreational, and/or artisanal fisheries across its entire range of distribution (Guzman and Tewfik 2004). Also, its abbreviated and relatively short larval period makes it a candidate for semi‐wild mariculture to support coral reef restoration efforts (Glover and Butler IV 2025).

Given its social, economic, and ecological importance, the life history of the Caribbean King crab is relatively well known (Provenzano and Brownell 1977; Baeza et al. 2012; Simpson et al. 2015; Turini et al. 2021 and references therein). A few genomic and genetic resources exist for this species (Baeza et al. 2010, 2019; Márquez et al. 2016; Hurtado‐Alarcón et al. 2018). Among them, the mitochondrial genome was assembled by Márquez et al. (2016) and a preliminary phylogeographic study of *Maguimithrax spinosissimus* suggested the existence of two genetic groups in the southern Caribbean Sea (Hurtado‐Alarcón et al. 2018). Most recently, though, its population genetics was reexamined using populations sampled in the south (Belize), central (Costa Rica), and northern Caribbean (Florida) basin. Population genetic analyses based on partial sequences of the mtDNA 12S, 16S, and COI genes indicated significant genetic differentiation among the studied populations, somewhat in line with connectivity matrices predicted by biophysical modeling (Baeza et al. 2019).

As part of a broad program to develop genomic resources for invertebrates of economic and ecological importance in the Caribbean basin, the aim of this study was to conduct a genomic survey of *M. spinosissimus* relaying on a set of bioinformatics tools recently developed to obtain biological insight from low sequencing‐depth next generation sequencing (NGS) data. Specifically, I (i) estimated nuclear genome size in *M. spinosissimus* using k‐mer profiling, (ii) explored transposable elements in the studied nuclear genome, (iii) assembled for a second time and described in detail the mitochondrial genome, and (iv) determined the phylogenetic position of *M. spinosissimus* utilizing the signal recovered from mitochondrial protein‐coding genes. The aforementioned genomic resources will support conservation initiatives in the Caribbean King Crab.

## 2 Methods

### 2.1 Specimen Collection, DNA Extraction, Library Preparation, and Sequencing

A specimen of *Maguimithrax spinosissimus* was collected during June, 2023, while SCUBA diving near Tennessee reef (24°44.7′ N, 80°46.9′ W), Florida Keys, USA. The specimen was transported to a temporary laboratory in Long Key, Florida Keys, USA, fixed in 95% ethyl alcohol, shipped to Clemson University, Clemson, South Carolina, USA, and deposited at the Clemson University Crustacean Collection. Next, a small muscle sample (approx. 2 mm^3^) was dissected from the first clawed pereiopod and inserted into a sterile 1.5 cm^3^ sterile centrifuge tube for genomic DNA (gDNA) extraction using the DNeasy Blood and Tissue Kit (Qiagen, Germany) following the manufacturer's protocol. gDNA was shipped to Novogene (Davis, California) for next generation library preparation and sequencing (NGS). As detailed in Baeza and Pirro (2024), library preparation was conducted using the Illumina TruSeq kit following the manufacturer's instructions and NGS was performed in an Illumina HiSeq X Ten system (Illumina, San Diego, CA, USA) using a 2 × 150 cycle.

### 2.2 Raw Reads Cleaning and Decontamination

Low quality sequences (Phred scores < 20) and adapters were removed from the raw reads generated by the sequencing facility with the pipeline fastp v.0.20.1 using default parameters (Chen et al. 2018) and the clean set of reads was ‘decontaminated’ from human, fungal, protozoan, bacterial, archaeal, and viral reads using the pipeline Kraken2 v2.1.2 (Wood and Salzberg 2014) and the kraken2‐microbial‐fatfree (https://lomanlab.github.io/mockcommunity/mc_databases.html) database.

### 2.3 Genome Size of *Damithrax spinosissimus*

Using the clean and decontaminated set of reads, the nuclear genome size of *M. spinosissimus* was estimated with the software KMC 3 v. 3.2.1 (Kokot et al. 2017) for 13 different k‐mer sizes (lengths); 18, 21, 24, 27, 30, 33, 36, 39, 42, 45, 48, 51, and 54 bp. After calculating k‐mer frequency distribution with KMC, I used the program REPeat SPECTra Estimation (RESPECT) v.1.0.0 (Sarmashghi et al. 2021) to estimate the nuclear genome size in *M. spinosissimus* .

### 2.4 The Nuclear ‘Repeatome’ of *Maguimithrax spinosissimus*

To examine transposable elements in the nuclear genome of *M. spinosissimus* , first, the clean and decontaminated set reads was mapped to the newly assembled mitochondrial genome of the same species (see below) with the pipeline HISAT2 v2.2.1 (Kim et al. 2019). I used the set of reads that didn't map to the mitochondrial genome (*n* = 72,688,900 PE reads [99.81%]) to discover, quantify, and annotate the transposable elements in the nuclear genome of *M. spinosissimus* using the program dnaPipeTE v1.4c (Goubert et al. 2015, Goubert et al. 2022). DnaPipeTE annotated transposable elements based on homology with the pipeline program RepeatModeler2 (Flynn et al. 2020) after assembling these elements with the program Trinity (Grabherr et al. 2011). As a last step, DnaPipeTE quantified transposable elements by mapping a small random sample of the used reads onto the assembled transposable elements. I ran DnaPipeTE using two iterations of the program Trinity with independent sets of read sampled each time at 0.15× (Goubert et al. 2022). I used the Protostomia‐specific database of transposable elements developed by the Dfam consortium (Hubley et al. 2016) in the analysis. Lastly, I calculated the transposable elements landscape in *M. spinosissimus* by calculating the divergence (blastn) between detected transposable element copies and their respective assembled consensus sequences (Goubert et al. 2022).

### 2.5 Mitochondrial Genome of *Maguimithrax spinosissimus*

The program GetOrganelle v1.6.4 (Jin et al. 2020) was employed to ‘de novo’ assemble the mitochondrial genome of the studied crab twice, once using as a ‘seed’ the complete mitochondrial genome of the conspecific *M. spinosssimus* from Colombia (GenBank's accession number NC025518—Márquez et al. 2016) and a second time using the cofamilial crab *Leptomithrax* sp. (MG571272—no companion paper). The two assembly runs used k‐mer sizes of 21, 55, 85, and 115 bp. Then, I used the pipeline MITOS2 (Donath et al. 2019), as implemented in the platform Galaxy (The Galaxy Community 2022), to annotate the newly assembled mitochondrial genome and a circular mitochondrial genome map was depicted using the web platform Chloroplot (https://irscope.shinyapps.io/Chloroplot/—Zheng et al. 2020). The software MEGA X (Kumar et al. 2018) was used to estimate nucleotide composition of the complete mitochondrial genome. In turn, to calculate codon usage and Relative Synonymous Codon Usage (RSCU) of all PCGs I used the web platforms Sequence Manipulation Suite (https://www.bioinformatics.org/sms2/codon_usage.html—Stothard 2000) and EZmito (http://ezmito.unisi.it—Cucini et al. 2021).

In the newly assembled mitochondrial genome, selective pressures affecting each PCG were analyzed with the software KaKs_calculator 2.0 (Wang et al. 2010). The number of nonsynonymous substitutions per nonsynonymous site (dN), the number of synonymous substitutions per synonymous site (dS), and the ratio dN/dS (= ω) were estimated using the cofamilial crab *Leptomithrax* sp. as the outgroup and the γ‐MYN model to reflect mutation rate variability along each of the aligned PCG orthologous sequences. The observed ω ratio is expected to be equal to 1, < 1, or > 1 if a particular PCG is exposed to neutral selection, purifying (negative), or diversifying (positive) selection, respectively (Wang et al. 2010).

The secondary structure of each tRNA gene present in the mitochondrial genome of *M. spinosssimus* was predicted using the bioinformatics tool MITFI (Jühling et al. 2012) and then visualized using the online platform Forna (http://rna.tbi.univie.ac.at/forna/—Kerpedjiev et al. 2015).

Finally, I explored for the existence of microsatellites (= Simple Sequence Repeats, SSRs) and/or short tandem repeats in the Control Region (CR) of the studied mitochondrial genome using, respectively, the web servers Microsatellites Repeats Finder (http://insilico.ehu.es/mini_tools/microsatellites—Bikandi et al. 2004) and Tandem Repeats Finder (https://tandem.bu.edu/trf/trf.html—Benson 1999). I also detected the presence of ‘stem and loops’ (= ‘hairpins’) in the same region using the online platform Forna.

### 2.6 Phylogenetic Position of *Maguimithrax spinosssimus*

A maximum likelihood (ML) phylogenomic analysis was conducted to explore the position of *M. spinosissimus* in the family Mithracidae using the signal recovered from translated mitochondrial PCGs. The ML phylogenomic analysis was executed with the newly assembled mitochondrial genome of *M. spinosissimus* , that of a second specimen from Colombia (Márquez et al. 2016), and other 11 other species belonging to the superfamily Majoidea with annotated mitochondrial genomes available in NCBI's GenBank. As outgroups, four species belonging to the superfamily Portunoidea, two species belonging to the superfamily Calappoidea, one species belonging to the superfamily Eriphoidea, and one species belonging to the superfamily Xanthoidea, were included. Before conducting the ML phylogenetic analysis, I aligned each set of PCG nucleotide sequences with the software MUSCLE (Edgar 2004) as implemented in the software MEGA X. Then, the program GBlocks (Castresana 2000) was employed to trim poorly aligned regions, if any, in each set of PCG alignments. Next, I concatenated each trimmed alignment and submitted the partitioned (per PCG) dataset to the web platform IQ‐TREE version 1.6.10 for ML analysis (Nguyen et al. 2015) that used the software ProtTest (Darriba et al. 2011) to select the best fitting models of sequence evolution for each partition. The robustness of the inferred ML tree was determined using the Shimodaira–Hasegawa approximate likelihood ratio test ([SH]‐aLRT) and 1000 (ultra‐fast) bootstrap iterations as in Baeza and Pirro (2024).

### 2.7 Microsatellite Discovery in *Maguimithrax spinosissimus*

Simple Sequence Repeats (SSRs) were discovered in the genome of *M. spinosssimus* using the program Krait2 (Du et al. 2025). Krait2 first used the program pytrf (https://github.com/lmdu/pytrf) to identify perfect and imperfect microsatellites in an assembly of the clean *M. spinosissimus* reads using the program SPAdes 4.0 (https://github.com/ablab/spades). After identifying perfect and imperfect SSRs (mono‐, di‐, tri‐, tetra‐, penta‐, and hexanucleotide motif repeats), Krait2 used the program primer3‐py (https://github.com/libnano/primer3‐py) for designing sets of primer pairs.

## 3 Results and Discussion

A total of 33,871,376 pairs (PE) reads were produced by the sequencing facility and deposited in the short read archive (SRA) repository (Bioproject: PRJNA780953, BioSample: SAMN46303996; SRA accession number: SRR32044727) at NCBI's GenBank. Out of the totality of these PE raw reads, 98.29% (*n* = 33,291,291) high quality PE reads remained after sequencing adapters and low‐quality sequences were removed with the software fastp and 2.38% (*n* = 792,332) of these high‐quality PE reads were classified as contaminants by the program Kraken2 (Figure 1). No reads were classified as of chordate, including human origin, while a total of 0.0321%, 0.756%, 0.705%, and 0.402% of the clean reads were classified as viral, bacterial, fungal, and protozoan, respectively. Overall, contamination was minimal in the studied dataset (Figure 1).

I note that an examination of the Kraken results and a blast of an assembly of the clean *M. spinosissimus* reads using the program Spades indicated a moderate prevalence of reads classified as belonging to the family Nimaviridae among viruses, and blastx‐matches to two viral species, *Armadillidium vulgare* clopovirus TUMSAT20210906 (LC738883) and *Hemigrapsus takanoi* Nimavirus TUMSAT‐1 (LC738882), known to infect crustaceans (Rampaul et al. 2024). Thus, I mapped the entire set of assembled contigs to the two aforementioned viral genomes using the platform ViralWasm (Ji et al. 2023). I obtained an incomplete and highly fragmented viral consensus assembly from the high‐quality *M. spinosissimus* reads in each case (total mapped length of the assembly contigs = 7.66% [31,883 bp of reads mapped out of 416,069 bp in the reference viral genome LC738883]) and 10.19% (25,670 bp of reads mapped out of 251,731 bp in the reference viral genome LC738882). I suggest that a novel undescribed viral species belonging to the family Nimaviridae infects *M. spinosissimus* and recommend that future metagenomics studies using gDNA extracted from the hepatopancreas dissected from visibly infected crabs (e.g., as in Bojko et al. 2023) might be successful in assembling a full viral genome of this putative new species predicted based on this analysis.

### 3.1 Nuclear Genome Size in *Maguimithrax spinosissimus*

In the Caribbean King Crab, the haploid nuclear genome size (GS) estimated with a k‐mer count strategy ranged between 2,164,858,053 bp (2.16 Gbp) using a k‐mer word size = 51 and 2,633,952,218 (2.63 Gbp) using a k‐mer word size = 21. Increases in k‐mer word size resulted in slight decreases in estimated genome size. Genome size estimates are uncommon in spider crabs, and genome size is available for one species of crab belonging to the family Mithracidae; in the cofamilial emerald crab *Mithraculus sculptus* , genome size, estimated using flow cytometry, is 3.29 Gbp (Hultgren et al. 2018). In majoid crabs (Superfamily Majoidea), genome size, estimated using either biochemical analysis, flow cytometry, static cell fluorometry, Feulgen densitometry, or Feulgen Image Analysis densitometry, varies between 2.16 Gbp in the common spider crab *Libinia emarginata* (Fam. Majidae) and 4.45 Gbp in *Libinia* sp. (Animal Genome Size Database [https://www.genomesize.com/]—Gregory 2021 [consulted on 01 08 2025]). Overall, the current estimation of genome size in *M. spinosissimus* is similar to that reported for a cofamilial species and within the range previously reported for other spider crabs.

### 3.2 Repetitive Elements in the Nuclear Genome of *Maguimithrax spinosissimus*

**FIGURE 2:**
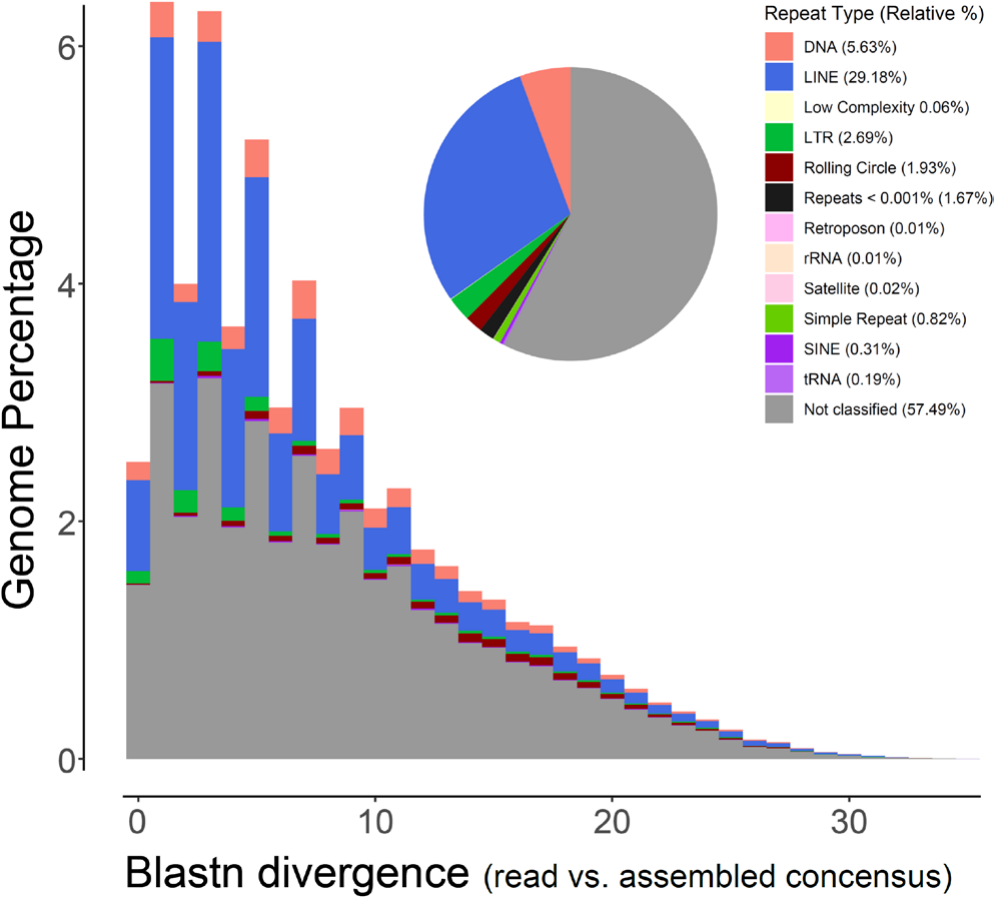
Repetitive elements genome composition and landscape in the nuclear genome of the Caribbean King Crab *Maguimithrax spinosissimus*.

In the Caribbean King Crab, the software REPEAT estimated nuclear repetitive genome content to vary between 49% (using k‐mer word size = 54) and 74% (with k‐mer word size = 18). The estimated repetitive genome content decreased with increases in k‐mer word size. The program dnaPipeTE estimated repetitive element content to be 53.53% in the Caribbean King Crab, a value that is within the range calculated by the pipeline REPEAT (Figure 2). Altogether, the results from the programs REPEAT and dnaPipeTE indicate that the ‘repeatome’ in the Caribbean King crab comprises between a half and three fourths of the nuclear genome. Repetitive content has not been estimated for any other majoid crab. However, among brachyuran crabs with assembled nuclear genomes, repetitive genome content varies between 36% in the blue crab *Callinectes sapidus* (Bachvaroff et al. 2021) and 58.97% in the mud crab *Scylla paramamosain* (Zhang et al. 2024).

Just over one half (57.49%) of the repetitive elements in the nuclear genome of the Caribbean King Crab were not annotated by the software DnaPipeTE (using the Protostomia specific transposable elements database—Dfam consortium) (Figure 2). If only annotated repetitive elements are taken into account, the most common were classified as Long Interspersed Nuclear Elements (LINEs, 29.18%). Much less common transposable elements included, among others, DNA transposons (5.63%), Long Terminal Repeats (LTRs, 2.69%), and Rolling Circles (RCs, 1.93%) (Figure 2). Repetitive element composition in the nuclear genome of the Caribbean King crab is similar to that reported for most of the few other brachyuran crabs with assembled nuclear genomes and whose ‘repeatome’ has been examined. For instance, LINEs, DNA transposons, and/or LTRs were the most common annotated repetitive elements in the nuclear genome of *Portunus trituberculatus* (Tang et al. 2020), *Eriocheir sinensis* (Cui et al. 2021) and *Cancer borealis* (Polinski et al. 2025). On the other hand, LINEs were commonly annotated repetitive elements in the nuclear genome of the blue crab *Callinectes sapidus* (Bachvaroff et al. 2021). However, in disagreement with our observations and previous reports in other brachyuran crabs, simple repeats were the second largest class of nuclear repeats in *C. sapidus* (Bachvaroff et al. 2021).

Lastly, the ‘landscape’ of transposable elements in the Caribbean King Crab exhibited a right‐skewed distribution with multiple peaks indicating consecutive ‘burst’ (= expansions) of repetitive elements in the recent past (Figure 2). The only other species of brachyuran crab in which the repetitive element landscape has been examined is *E. sinensis* with repetitive elements that have experienced expansions both in the recent and distant past in this species (Cui et al. 2021).

Transposable elements in the nuclear genome of decapod crustaceans, including brachyuran crabs, have rarely been characterized (Baeza and Pirro 2024 and references therein). Future studies analyzing repetitive elements composition in detail are expected to provide insight regarding their role as drivers of genome size and architecture (Shapiro 2005; Helmkampf et al. 2019) and evolutionary novelties (e.g., Werren 2011), among others, not only in crabs but also in decapod crustaceans. Most recently, repetitive elements have been suggested to impact the ability of their host individuals to react to environmental affronts (Casacuberta and González 2013). The role that transposable elements might have to affect the ability of *M. spinosissimus* and other crabs to respond to global change needs to be examined.

### 3.3 Mitochondrial Genome of *Maguimithrax spinosissimus*

**FIGURE 3:**
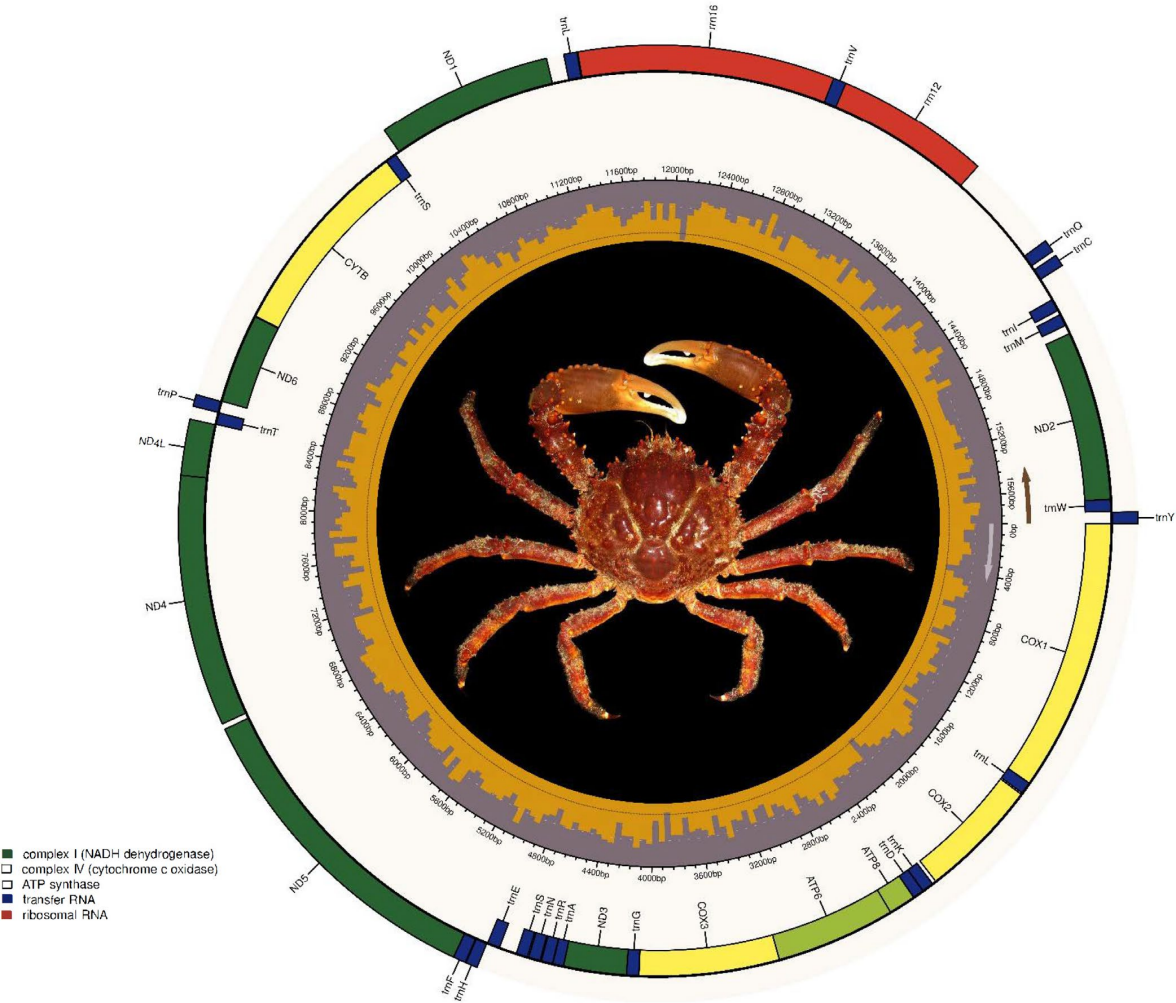
Circular depiction of the mitochondrial genome of the Caribbean King Crab *Maguimithrax spinosissimus* and differences between the newly assembled mitochondrial genome and a previously assembled mitochondrial genome from a specimen captured in Colombia (bottom) Photograph credit: J. Antonio Baeza (published with permission).

The software GetOrganelle assembled a circularized (= complete) mitochondrial genome for *M. spinosissimus* with an average coverage (per bp) of 786× and 470× when using *Leptomithrax* sp. (MG571272) and *M. spinosissimus* (NC025518) as a seed, respectively. The two assemblies were identical (GeneBank accession number OR612316), had a length of 15,714 bp, were AT‐rich (A + T content = 71.06%), and coded for two ribosomal RNA genes (12S and 16S ribosomal RNA), 22 transfer RNA (tRNA) genes, and 13 protein‐coding genes (PCGs) (Table 1). Also, the newly assembled mitochondrial genome included a D‐loop or Control Region (CR) 528 bp long (Figure 3, Table 1). The newly assembled mitochondrial genome was a close match to the previously assembled mitochondrial genome from a specimen collected in Colombia (Hurtado‐Alarcón et al. 2018) with patristic [p]—distance = 0.0028004073 and a few single nucleotide polymorphisms (*n* = 44) and indels (*n* = 5) differentiating the assemblies other than a 99 bp non‐coding fragment only present in the previously assembled mitogenome (positions: 4746–4844, motif: 5′‐TTA CTC CGT TAT TAC TCC GTT ATT ACT CCG TTA TTA CTC CGT TAT TAC TCC GTT TTT ACT CCG TTT TTA CTC CGT TTT TAC TCC GTT TTT ACT CCG TTT—3′) (Figure 3, Figure S1).

**TABLE 1:** Mitochondrial genome of the Caribbean King Crab *Maguimithrax spinosissimus*. Arrangement and annotation.

Name	Type	Start	Stop	Strand	Length (bp)	Start	Stop	Continuity
*cox1*	PCG	1	1539	(+)	1539	ATG	TAA	−5
trnL2(taa)	tRNA	1535	1602	(+)	68			8
*cox2*	PCG	1611	2291	(+)	681	ATG	TAA	−22
trnK(ttt)	tRNA	2313	2379	(+)	67			0
trnD(gtc)	tRNA	2380	2442	(+)	63			0
*atp8*	PCG	2443	2601	(+)	159	ATT	TAA	−5
*atp6*	PCG	2595	3269	(+)	675	ATT	TAA	−1
*cox3*	PCG	3269	4078	(+)	810	ATG	TAA	−19
trnG(tcc)	tRNA	4059	4127	(+)	69			0
*nad3*	PCG	4128	4481	(+)	354	ATT	TAA	−1
trnA(tgc)	tRNA	4481	4542	(+)	62			1
trnR(tcg)	tRNA	4544	4608	(+)	65			6
trnN(gtt)	tRNA	4615	4681	(+)	67			7
trnS1(tct)	tRNA	4689	4753	(+)	65			−1
trnE(ttc)	tRNA	4753	4823	(+)	71			3
trnH(gtg)	tRNA	4827	4890	(−)	64			6
trnF(gaa)	tRNA	4893	4960	(−)	68			61
*nad5*	PCG	4897	6612	(−)	1716	ATT	TAA	8
*nad4*	PCG	6721	8040	(−)	1320	ATG	TAA	20
*nad4l*	PCG	8061	8360	(−)	300	ATG	TAA	2
trnT(tgt)	tRNA	8363	8429	(+)	67			0
trnP(tgg)	tRNA	8430	8493	(−)	64			4
*nad6*	PCG	8496	9002	(+)	507	ATT	TAA	3
*cob*	PCG	9006	10142	(+)	1137	ATG	TAA	−1
trnS2(tga)	tRNA	10141	10205	(+)	65			26
*nad1*	PCG	10231	11169	(−)	939	ATG	TAA	92
trnL1(tag)	tRNA	11262	11332	(−)	71			−31
rrnL	rRNA	11363	12711	(−)	1349			9
trnV(tac)	tRNA	12702	12773	(−)	72			−2
rrnS	rRNA	12771	13595	(−)	825			
OL		13931	13954	(−)	24			
OH		14053	14102	(+)	50			
trnQ(ttg)	tRNA	14124	14191	(−)	68			27
trnC(gca)	tRNA	14218	14281	(−)	64			127
trnI(gat)	tRNA	14408	14476	(+)	69			26
trnM(cat)	tRNA	14502	14569	(+)	68			3
*nad2*	PCG	14573	15577	(+)	1005	ATT	TAG	−2
trnW(tca)	tRNA	15576	15643	(+)	68			4
trnY(gta)	tRNA	15648	15714	(−)	67			0

The length and AT‐content of the mitochondrial genome herein assembled for *M. spinosissimus* are similar to those reported for the conspecific from Colombia (15,817 bp and AT‐content = 70.1%—Márquez et al. 2016) and are within the range reported before for cofamilial species; in the fam. Mithracidae; mitochondrial genome length ranges between 15,551 bp in *Chionoecetes bairdi* (OP429109) and 16,608 bp in *Leptomithrax* sp. (MG571272). Also, mitochondrial gene order in *M. spinosissimus* is identical to that reported before for the conspecific from Colombia (Márquez et al. 2016) but different from that reported for all other representatives of the superfamily Majoidea (Basso et al. 2017). Indeed, gene order is variable in spider crabs and whether mitochondrial synteny is useful to reveal phylogenetic relationships in this clade remains to be addressed (Basso et al. 2017).

**FIGURE 4:**
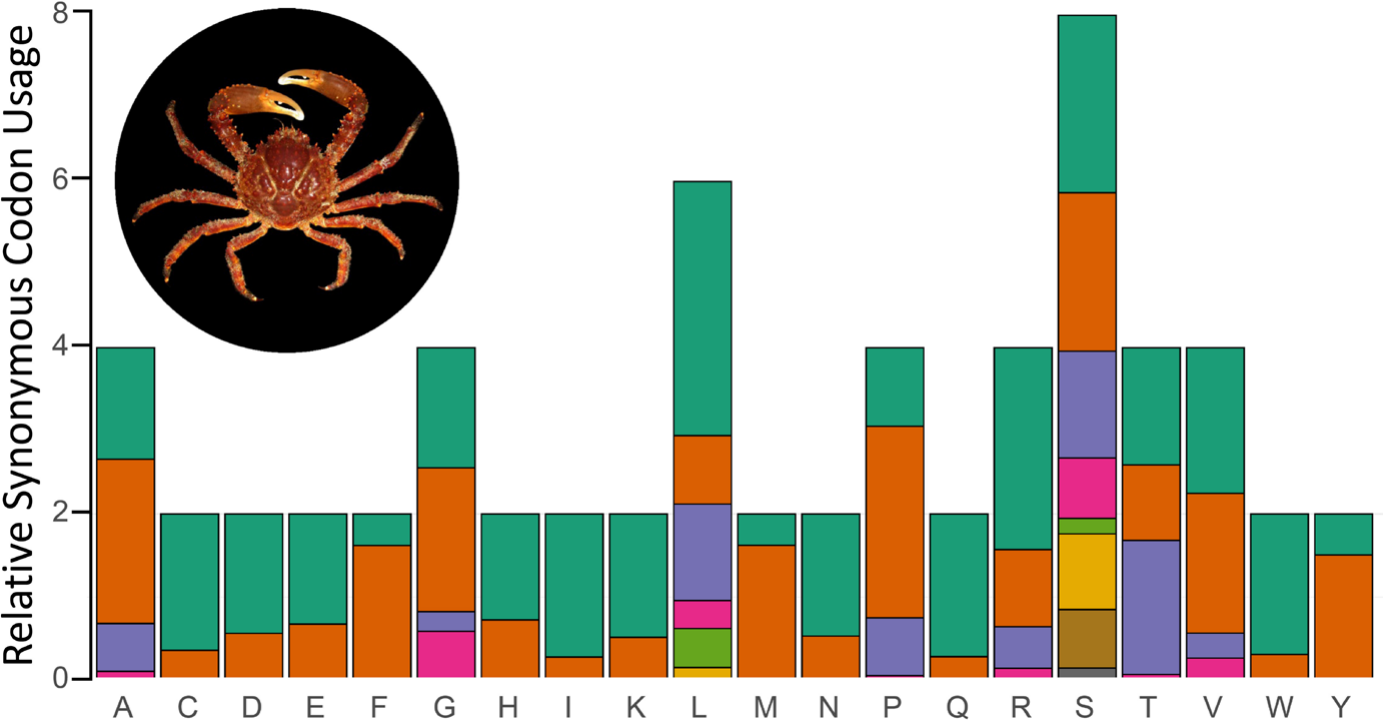
Relative synonymous usage in the 13 protein‐coding genes encoded in the mitochondrial genome of the Caribbean King Crab *Maguimithrax spinosissimus*. Amino Acid (1‐letter), Amino Acid (Name), and synonymous codons (from top to bottom color in the graph) are: A = Alanine: GCU, GCC, GCA, GCG. C = Cysteine: UGU, UGC. D = Aspartic Acid: GAU, GAC. E = Glutamic Acid: GAA, GAG. F = Phenylalanine: UUU, UUC. G = Glycine: GGU, GGC, GGA, GGG. H = Histidine: CAU, CAC. I = Isoleucine: AUU, AUC. K = Lysine: AAA, AAG. L = Leucine: UUA, UUG, CUU, CUC, CUA, CUG. M = Methionine: AUA, AUG. *N* = Asparagine: AAU, AAC. *P* = Proline: CCU, CCC, CCA, CCG. Q = Glutamine: CAA, CAG. *R* = Arginine: CGU, CGC, CGA, CGG. S = Serine: UCU, UCC, UCA, UCG, AGU, AGC. T = Threonine: ACU, ACC, ACA, ACG. V = Valine: GUU, GUC, GUA, GUG. W = Tryptophan: UGA, UGG. Y = Tyrosine: UAU, UAC.

In the mitochondrial genome of the Caribbean King Crab, all 13 PCGs started and ended with canonical codons (Table 1) and codons in each PCG (and overall) were not evenly used. Codons most often used were AT‐rich, including TTT (Phe, *n* = 304 times used), TTA (Leu, *n* = 300), and ATT (Ile, *n* = 279), while least often used codons (other than stop codons) were GC‐rich; CCG (Pro, *n* = 2), CGC (Arg, *n* = 2), ACG (Thr, *n* = 3), and GCG (Ala, *n* = 5), among a few others (Table S1). Also, among synonymous codons, most frequently used codons were AT‐rich as indicated by the RSCU analysis (Figure 4). No study describing the mitochondrial genome of majoid crabs has examined RSCU. However, our results are similar to those reported for other brachyuran crabs, in which AT‐rich codons are most frequently used than CG‐codons (Baeza and Pirro 2024). Codon usage likely explains the AT rich nature of the mitochondrial genome of the studied species (Bernt et al. 2013).

In the mitochondrial genome of *M. spinosissimus* , all PCGs experience purifying selection considering that all calculated KA/KS ratios exhibited values < 1 in our analysis of selective pressures (Table 2). The KA/KS ratios calculated for *atp6*, *cob*, *cox1*, *cox2*, *cox3*, *nad1*, and *nad3* were one order of magnitude lower than those estimated for *atp8*, *nad2*, *nad4*, *nad5*, *nad6*, and *nad4l*, suggesting stronger evolutionary constraints in the former seven than in the latter five genes (Table 2). In brachyuran crabs, selective pressure analyses are rarely conducted (Baeza 2021; O'Brien et al. 2022) and no selective pressure analyses have been conducted before in spider crabs (Majoidea). Still, our results are in agreement with previous studies in other decapod crustaceans that have revealed widespread purifying selection in mitochondrial PCGs (Baeza 2021, O'Brien et al. 2022, and references therein).

**TABLE 2:** Selective pressure analysis of the protein‐coding genes in the mitochondrial genome of the Caribbean King Crab *Maguimithrax spinosissimus*.

PCG	KA	KS	KA/KS	*p*
*atp6*	0.061697	3.48444	0.017706	NA
*atp8*	0.32888	2.41971	0.135918	5.41E‐08
*cob*	0.03739	4.03779	0.00926	4.04E‐155
*cox1*	0.016359	4.25557	0.003844	2.55E‐205
*cox2*	0.039626	3.58027	0.011068	4.26E‐103
*cox3*	0.042756	2.17458	0.019662	4.90E‐72
*nad1*	0.096854	2.61704	0.037009	3.41E‐67
*nad2*	0.198108	3.80937	0.052005	1.69E‐59
*nad3*	0.099574	2.97487	0.033472	4.58E‐41
*nad4*	0.135369	2.06572	0.065531	5.28E‐62
*nad4l*	0.111365	1.41492	0.078708	1.13E‐14
*nad5*	0.158766	2.92671	0.054247	2.53E‐73
*nad6*	0.238102	3.58394	0.066436	9.47E‐32

**FIGURE 5:**
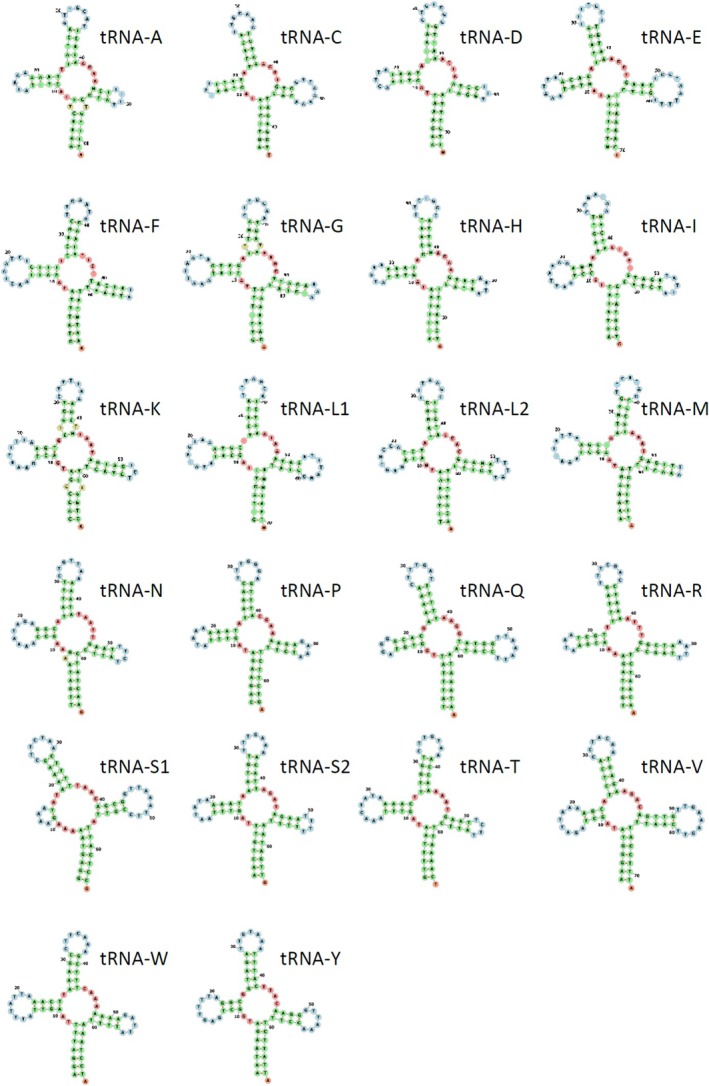
Secondary structure prediction of tRNA genes encoded in the mitochondrial genome of the Caribbean King Crab *Maguimithrax spinosissimus*.

In the mitochondrial genome of the Caribbean King Crab, the length of the tRNA genes varied between 62 pb (tRNA‐A) and 72 bp (tRNA‐V). All the tRNA genes exhibited a canonical ‘cloverleaf’ secondary structure with the exception of 3 genes; tRNA‐S1 that bear a short loop compared to other tRNA genes and tRNA‐F and tRNA‐M, each missing the T‐loop (Figure 5). The genes tRNA‐S1 and/or tRNA‐S2 but not tRNA‐F and tRNA‐M are commonly reported as truncated in eumetazoans, including brachyuran crabs (Bernt et al. 2013). Importantly, it is not clear if truncated tRNA genes are functional (Bernt et al. 2013). Limited studies suggest that truncated tRNAs can be functionals if they coevolve with interacting factors (seryl‐tRNA synthetase) that allows them to recognize non‐canonical tRNAs (Palopoli et al. 2014).

In *M. spinosissimus* , the AT‐rich (69.51%) mitochondrial CR is located between the 12 S ribosomal RNA and the tRNA‐Q genes (Figure 3). A total of nine microsatellites were found in the CR of the studied mitochondrial genome. All of them were dinucleotide SRRs repeated between 3 and 7 times, and 8 out of the 9 SRRs were AT‐rich (Table S2). In turn, no tandem repeats were detected in the studied CR by the program Tandem Repeat Finder. Finally, prediction of the secondary structure in the platform forna denoted the presence of several stem‐loop structures distributed across the entirety of the CR (Figure S2). The CR also contained the origin of replication of the heavy and light strands (Table 1). No detailed studies of the CR has been conducted in other spider crabs. Nonetheless, our observations are in agreement with previous analyses focusing on the CR of other brachyuran crabs and decapod crustaceans; microsatellites and/or short tandem repeats and stem‐and‐loops are common (Baeza 2018 and references therein). Studies analyzing in detail the structure of the CR are necessary in other spider crabs, decapod crustaceans, and invertebrates, to improve our understanding of its function during mitochondrial transcription and replication in non‐model organisms.

### 3.4 Microsatellite Discovery in *Maguimithrax spinosissimus*

A total of 578,279 and 485,200 perfect and imperfect SSRs, respectively, were detected by the program Krait2 and most of these SSRs were di‐nucleotides (27.91% and 24.42%, respectively) (Table 3). A total of 810 SSR primer pairs were identified using the software Krait2 (File S5). Studies using the SSRs identified here (after further development) can be used to examine (i) multiple paternity, (ii) meta‐population genetic structure, and (ii) cryptic species in *Maguimithrax spinosissimus* (see Baeza et al. 2019 and references therein).

**TABLE 3:** Summary statistics for perfect and imperfect microsatellites in the nuclear genome of the Caribbean King Crab *Maguimithrax spinosissimus*.

Perfect microsatellite						
Summary statistics						
Total counts	Total length (bp)	Average length (bp)	Sequence coverage (%)	Relative abundance (loci/Mb)	Relative density (bp/Mb)	
578,279	14,153,726	24.48	1.74	711.71	17419.54	
**Motif type statistics**						
**Motif type**	**Total count**	**Total length (bp)**	**Percentage (%)**	**Average length (bp)**	**Frequency (loci/Mb)**	**Density (bp/Mb)**
Mono	126,086	1,940,056	21.8	15.39	155.18	2387.7
Di	161,413	5,502,386	27.91	34.09	198.66	6772
Tri	153,997	3,705,378	26.63	24.06	189.53	4560.35
Tetra	111,759	2,355,476	19.33	21.08	137.55	2898.98
Penta	23,311	604,140	4.03	25.92	28.69	743.54
Hexa	1713	46,290	0.3	27.02	2.11	56.97

Imperfect microsatellite						
Summary statistics						
Total counts	Total length (bp)	Average length (bp)	Sequence coverage (%)	Relative abundance (loci/Mb)	Relative density (bp/Mb)	
485,200	1.15E + 08	236.49	14.12	597.15	141223.4	
**Motif type statistics**						
**Motif type**	**Total count**	**Total length (bp)**	**Percentage (%)**	**Average length (bp)**	**Frequency (loci/Mb)**	**Density (bp/Mb)**
Mono	190,153	48,488,610	39.19	255	234.03	59676.81
Di	118,476	29,166,063	24.42	246.18	145.81	35895.8
Tri	74,783	15,578,037	15.41	208.31	92.04	19172.49
Tetra	72,152	15,068,119	14.87	208.84	88.8	18544.92
Penta	24,478	5,299,271	5.04	216.49	30.13	6522.02
Hexa	5158	1,146,711	1.06	222.32	6.35	1411.3

### 3.5 Phylogenetic Position of *Maguimithrax spinosissimus*

**FIGURE 6:**
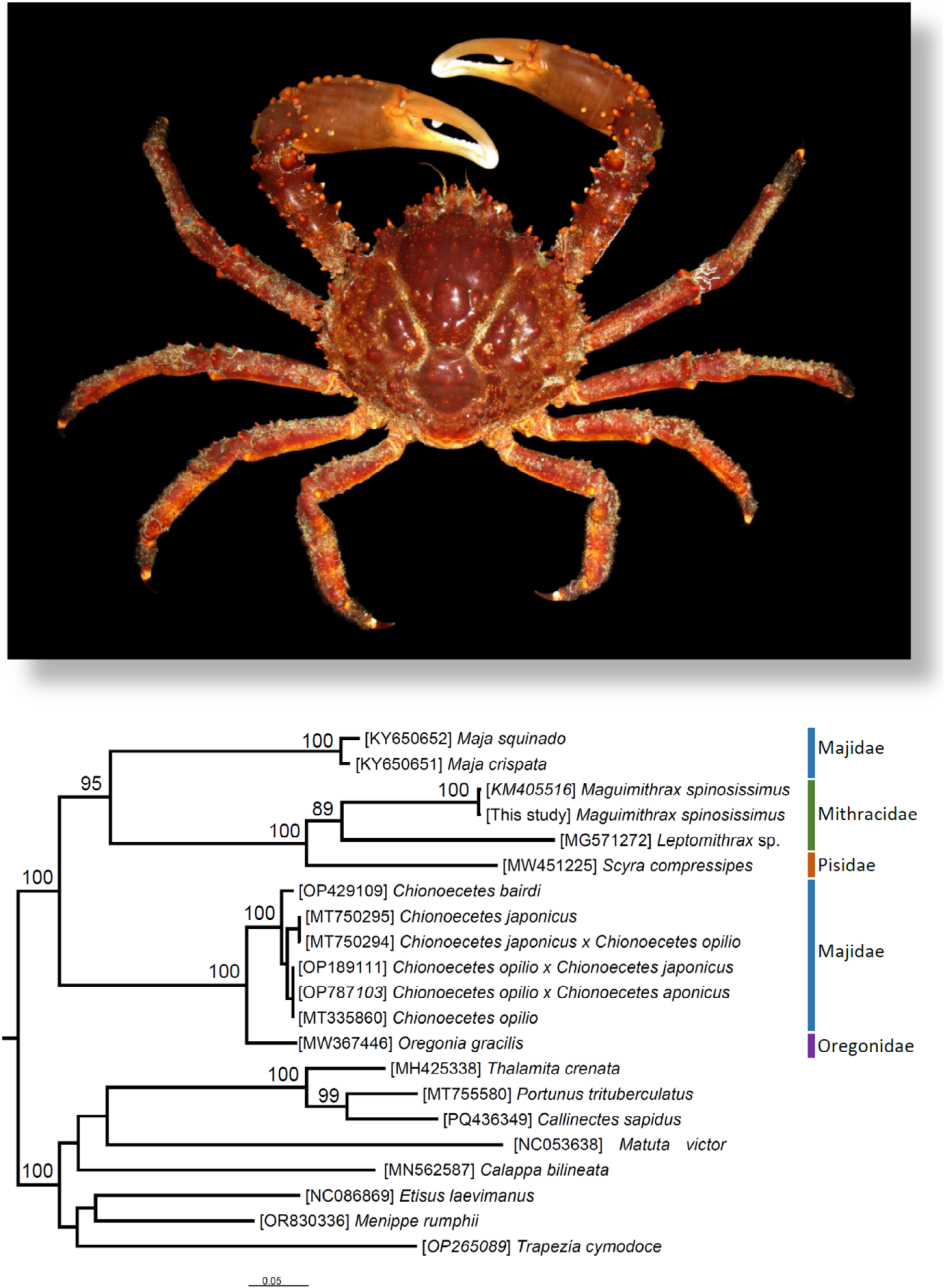
Maximum likelihood phylogenetic analysis of the superfamily Majoidea and phylomitogenomic position of the Caribbean King Crab *Maguimithrax spinosissimus*. Numbers above branches near nodes represent bootstrap pseudoreplicates of the tree search. Photograph credit: J. Antonio Baeza (published with permission).

The ML phylogenetic analysis included a total of 21 terminal nodes and a matrix of 3660 amino acids of which 1968 were parsimony‐informative. The superorder Majoidea was fully supported as monophyletic (boostrap value [bv] = 100) in our analysis (Figure 6). On the other hand, in the superorder Majoidea, the analysis did not support the monophyletic status of the family Majidae considering that representatives of the genera *Chionoecetes* (belonging to the family Majidae) and *Oregonia gracilis* (belonging to the family Oregonidae) clustered into a fully supported clade that was sister to a second moderately to well supported clade (bv = 95) that comprised representatives of the genera *Maja* (fam. Majidae), *Scyra* (fam. Pisidae), and *Maguimithrax spinosissimus* (two specimens) plus *Leptomithrax*, the latter two genera belonging to the family Mithracidae. In the latter clade, the genus *Maja* was sister to a fully supported clade that included *Scyra* + *Maguimithrax* + *Leptomithrax*. Overall, the recovered phylogenetic relationships within the superfamily Majoidea are in line with the most recent phylomitogenomic analysis conducted by Wang et al. (2024). Additional studies focusing on the assembly of mitochondrial genomes belonging to the superfamily Majoidea are needed to understand the evolutionary history of this remarkable clade of crabs.

## 4 Conclusion

In this study, a detailed analysis of nuclear and mitochondrial genomic features in *M. spinosissimus* was conducted, the largest crab in the Caribbean basin that is the target of subsistence, recreational, and artisanal fisheries across most of its distribution and that likely plays an important ecological role. A set of bioinformatics tools tailored for low‐sequencing‐depth (= low pass) short‐read sequencing enabled the estimation of nuclear genome size, classification and quantification of transposable elements in the nuclear genome, assembly and detailed analysis of the mitochondrial genome, and exploration of phylogenetic relations in the superfamily Majoidea based on the information provided by translated mitochondrial PCGs. The data also suggest the presence of an undescribed viral species infecting the Caribbean King Crab. I expect that the results from this study will help optimize resources to assemble a chromosome‐level nuclear genome in the Caribbean King Crab. The assembled mitogenome is expected to support biomonitoring of this species in coral reefs using environmental DNA (eDNA). Specifically, full mitochondrial genomes are useful to develop new and better markers that can facilitate estimating the abundance of coral‐reef dwelling species, currently a main goal in eDNA research (Ai et al. 2024 and references therein). Overall, the developed genomic resources can be used to support conservation and fisheries management initiatives in *M. spinosissimus* , the largest crab in the Caribbean basin with potential for aquaculture to support coral reef restoration efforts.

## Author Contributions

**Juan Antonio Baeza:** conceptualization (equal), data curation (equal), formal analysis (equal), investigation (equal), methodology (equal), project administration (equal), resources (equal), supervision (equal), validation (equal), visualization (equal), writing – original draft (equal), writing – review and editing (equal).

## Conflicts of Interest

The author declares no conflicts of interest.

## Supporting information


**Table S1.** Codon usage analysis in the mitochondrial protein‐coding genes of the Caribbean King Crab *Maguimithrax spinosissimus*.
**Table S2.** Microsatellites in the Control Region of the Caribbean King Crab *Maguimithrax spinosissimus* detected by the online tool Microsatellite repeats finder.
**Figure S1.** Comparison of SNP differences between the newly assembled mitochondrial genome in this study and a previously published mitochondrial genome of the Caribbean King Crab *Maguimithrax spinosissimus*.
**Figure S2.** Secondary structure prediction of the control region in the mitochondrial genome of the Caribbean King Crab *Maguimithrax spinosissimus*.

## Data Availability

Sequences described here are accessible via GenBank: BioSample: SAMN46303996; SRA accession number: SRR32044727l; BioProject ID: PRJNA780953.

## References

[ece371619-bib-0001] Ai, Q. , H. Yuan , Y. Wang , and C. Li . 2024. “Estimation of Species Abundance Based on the Number of Segregating Sites Using Environmental DNA (eDNA).” Molecular Ecology Resources: e14076. 10.1111/1755-0998.14076. 39912119

[ece371619-bib-0002] Bachvaroff, T. R. , R. C. McDonald , L. V. Plough , and J. S. Chung . 2021. “Chromosome‐Level Genome Assembly of the Blue Crab, *Callinectes sapidus* .” G3: Genes—Genomes—Genetics 11, no. 9: jkab212. 10.1093/g3journal/jkab212. 34544121 PMC8496215

[ece371619-bib-0003] Baeza, J. A. 2018. “The Complete Mitochondrial Genome of the Caribbean Spiny Lobster *Panulirus argus* .” Scientific Reports 8, no. 1: 17690. 10.1038/s41598-018-36132-6. 30523272 PMC6283867

[ece371619-bib-0004] Baeza, J. A. 2021. “A First Genomic Portrait of the Florida Stone Crab *Menippe mercenaria* : Genome Size, Mitochondrial Chromosome, and Repetitive Elements.” Marine Genomics 57: 100821. 10.1016/j.margen.2020.100821. 33867116

[ece371619-bib-0005] Baeza, J. A. , J. R. Anderson , A. J. Spadaro , and D. C. Behringer . 2012. “Sexual Dimorphism, Allometry, and Size at First Maturity of the Caribbean King Crab, *Mithrax spinosissimus* , in the Florida Keys.” Journal of Shellfish Research 31, no. 4: 909–916.

[ece371619-bib-0006] Baeza, J. A. , J. A. Bolaños , S. Fuentes , J. E. Hernandez , C. Lira , and R. López . 2010. “Molecular Phylogeny of Enigmatic Caribbean Spider Crabs From the *Mithrax*–*Mithraculus* Species Complex (Brachyura: Majidae: Mithracinae): Ecological Diversity and a Formal Test of Genera Monophyly.” Journal of the Marine Biological Association of the United Kingdom 90, no. 4: 851–858.

[ece371619-bib-0007] Baeza, J. A. , D. Holstein , R. Umaña‐Castro , and L. M. Mejía‐Ortíz . 2019. “Population Genetics and Biophysical Modeling Inform Metapopulation Connectivity of the Caribbean King Crab *Maguimithrax spinosissimus* .” Marine Ecology Progress Series 610: 83–97.

[ece371619-bib-0008] Baeza, J. A. , and S. Pirro . 2024. “Genomics Resources for the Rapa Nui (Eastern Island) Spiny Lobster *Panulirus pascuensis* (Crustacea: Decapoda: Achelata).” Revista Chilena de Historia Natural 97, no. 1: 9. 10.1186/s40693-024-00132-w.

[ece371619-bib-0009] Basso, A. , M. Babbucci , M. Pauletto , E. Riginella , T. Patarnello , and E. Negrisolo . 2017. “The Highly Rearranged Mitochondrial Genomes of the Crabs *Maja crispata* and *Maja squinado* (Majidae) and Gene Order Evolution in Brachyura.” Scientific Reports 7, no. 1: 4096. 10.1038/s41598-017-04168-9. 28642542 PMC5481413

[ece371619-bib-0010] Benson, G. 1999. “Tandem Repeats Finder: A Program to Analyze DNA Sequences.” Nucleic Acids Research 27: 573–580. 10.1093/nar/27.2.573. 9862982 PMC148217

[ece371619-bib-0011] Bernt, M. , A. Donath , F. Jühling , et al. 2013. “MITOS: Improved De Novo Metazoan Mitochondrial Genome Annotation.” Molecular Phylogenetics and Evolution 69: 313–319. 10.1016/j.ympev.2012.08.023. 22982435

[ece371619-bib-0012] Bikandi, J. , R. San Millán , A. Rementeria , and J. Garaizar . 2004. “In Silico Analysis of Complete Bacterial Genomes: PCR, AFLP‐PCR, and Endonuclease Restriction.” Bioinformatics 20: 798–799. 10.1093/bioinformatics/btg491. 14752001

[ece371619-bib-0013] Bojko, J. , E. Duermit‐Moreau , R. Gandy , and D. C. Behringer . 2023. “A New Member of the Nudiviridae From the Florida Stone Crab ( *Menippe mercenaria* ).” Virology 588: 109910. 10.1016/j.virol.2023.109910. 37844408

[ece371619-bib-0015] Casacuberta, E. , and J. González . 2013. “The Impact of Transposable Elements in Environmental Adaptation.” Molecular Ecology 22, no. 6: 1503–1517. 10.1111/mec.12170. 23293987

[ece371619-bib-0016] Castresana, J. 2000. “Selection of Conserved Blocks From Multiple Alignments for Their Use in Phylogenetic Analysis.” Molecular Biology and Evolution 17: 540–552. 10742046 10.1093/oxfordjournals.molbev.a026334

[ece371619-bib-0017] Chen, S. , Y. Zhou , Y. Chen , and J. Gu . 2018. “Fastp: An Ultra‐Fast All‐In‐One FASTQ Preprocessor.” Bioinformatics 34, no. 17: i884–i890. 10.1093/bioinformatics/bty560. 30423086 PMC6129281

[ece371619-bib-0018] Cucini, C. , C. Leo , N. Iannotti , et al. 2021. “EZmito: A Simple and Fast Tool for Multiple Mitogenome Analyses.” Mitochondrial DNA Part B Resources 6: 1101–1109. 10.1080/23802359.2021.1899865. 33796755 PMC7995877

[ece371619-bib-0019] Cui, Z. , Y. Liu , J. Yuan , et al. 2021. “The Chinese Mitten Crab Genome Provides Insights Into Adaptive Plasticity and Developmental Regulation.” Nature Communications 12, no. 1: 2395. 10.1038/s41467-021-22604-3. PMC8062507 33888695

[ece371619-bib-0020] Darriba, D. , G. L. Taboada , R. Doallo , and D. Posada . 2011. “ProtTest 3: Fast Selection of Best‐Fit Models of Protein Evolution.” Bioinformatics 27: 1164–1165. 10.1093/bioinformatics/btr088. 21335321 PMC5215816

[ece371619-bib-0021] Donath, A. , F. Jühling , M. Al‐Arab , et al. 2019. “Improved Annotation of Protein‐Coding Genes Boundaries in Metazoan Mitochondrial Genomes.” Nucleic Acids Research 47: 10543–10552. 10.1093/nar/gkz833. 31584075 PMC6847864

[ece371619-bib-0022] Du, L. , J. Chen , D. Sun , K. Zhao , Q. Zeng , and N. Yang . 2025. “Krait2: A Versatile Software for Microsatellite Investigation, Visualization and Marker Development.” BMC Genomics 26, no. 1: 72. 10.1186/s12864-025-11252-2. 39863857 PMC11762079

[ece371619-bib-0023] Edgar, R. C. 2004. “MUSCLE: Multiple Sequence Alignment With High Accuracy and High Throughput.” Nucleic Acids Research 32, no. 5: 1792–1797. 10.1093/nar/gkh340. 15034147 PMC390337

[ece371619-bib-0024] Flynn, J. M. , R. Hubley , C. Goubert , et al. 2020. “RepeatModeler2 for Automated Genomic Discovery of Transposable Element Families.” Proceedings of the National Academy of Sciences 117, no. 17: 9451–9457. 10.1073/pnas.1921046117. PMC7196820 32300014

[ece371619-bib-0025] Glover, S. , and M. J. Butler IV . 2025. “The Semi‐Wild Mariculture of Caribbean King Crab (*Maguimithrax spinosissimus*) for Coral Reef Restoration.” Aquaculture Reports 40: 102612. 10.1016/j.aqrep.2024.102612.

[ece371619-bib-0026] Goubert, C. , R. J. Craig , A. F. Bilat , V. Peona , A. A. Vogan , and A. V. Protasio . 2022. “A Beginner's Guide to Manual Curation of Transposable Elements.” Mobile DNA 13: 7. 10.1186/s13100-021-00259-7. 35354491 PMC8969392

[ece371619-bib-0027] Goubert, C. , L. Modolo , C. Vieira , C. ValienteMoro , P. Mavingui , and M. Boulesteix . 2015. “De Novo Assembly and Annotation of the Asian Tiger Mosquito (*Aedes albopictus*) Repeatome With DNAPipeTE From Raw Genomic Reads and Comparative Analysis With the Yellow Fever Mosquito (*Aedes aegypti*).” Genome Biology and Evolution 7: 1192–1205. 10.1093/gbe/evv050. 25767248 PMC4419797

[ece371619-bib-0028] Grabherr, M. G. , B. J. Haas , M. Yassour , et al. 2011. “Trinity: Reconstructing a Full‐Length Transcriptome Without a Genome From RNA‐Seq Data.” Nature Biotechnology 29, no. 7: 644. 10.1038/nbt.1883. PMC3571712 21572440

[ece371619-bib-0029] Gregory, T. R. 2021. “Animal Genome Size Database.” http://www.genomesize.com.

[ece371619-bib-0030] Guzman, H. M. , and A. Tewfik . 2004. “Population Characteristics and Co‐Occurrence of Three Exploited Decapods (*Panulirus argus*, *P. guttatus*, *Mithrax spinosissimus*) in Bocas del Toro, Panama.” Journal of Shellfish Research 23, no. 2: 575–580.

[ece371619-bib-0031] Helmkampf, M. , M. R. Bellinger , S. M. Geib , S. B. Sim , and M. Takabayashi . 2019. “Draft Genome of the Rice Coral *Montipora capitata* Obtained From Linked‐Read Sequencing.” Genome Biology and Evolution 11: 2045–2054. 10.1093/gbe/evz135. 31243452 PMC6668484

[ece371619-bib-0032] Hubley, R. , R. D. Finn , J. Clements , et al. 2016. “The Dfam Database of Repetitive DNA Families.” Nucleic Acids Research 44, no. D1: D81–D89. 10.1093/nar/gkv1272. 26612867 PMC4702899

[ece371619-bib-0033] Hultgren, K. M. , N. W. Jeffery , A. Moran , and T. R. Gregory . 2018. “Latitudinal Variation in Genome Size in Crustaceans.” Biological Journal of the Linnean Society 123: 348–359.

[ece371619-bib-0034] Hurtado‐Alarcón, J. C. , N. H. Campos Campos , A. Bermudez Tobon , and E. J. Márquez . 2018. “Phylogeographic Patterns in *Maguimithrax spinosissimus* (Decapoda: Mithracidae) From Colombian Caribbean.” New Zealand Journal of Marine and Freshwater Research 52: 118–137.

[ece371619-bib-0035] Ji, D. , R. Aboukhalil , and N. Moshiri . 2023. “ViralWasm: A Client‐Side User‐Friendly Web Application Suite for Viral Genomics.” Bioinformatics 40: btae018. 10.1093/bioinformatics/btae018. PMC10809900 38200583

[ece371619-bib-0036] Jin, J. J. , W. B. Yu , J. B. Yang , et al. 2020. “GetOrganelle: A Fast and Versatile Toolkit for Accurate De Novo Assembly of Organelle Genomes.” Genome Biology 21: 1–31. 10.1186/s13059-020-02154-5. PMC7488116 32912315

[ece371619-bib-0037] Jühling, F. , J. Pütz , M. Bernt , et al. 2012. “Improved Systematic tRNA Gene Annotation Allows New Insights Into the Evolution of Mitochondrial tRNA Structures and Into the Mechanisms of Mitochondrial Genome Rearrangements.” Nucleic Acids Research 40, no. 7: 2833–2845. 22139921 10.1093/nar/gkr1131 PMC3326299

[ece371619-bib-0038] Kerpedjiev, P. , S. Hammer , and I. L. Hofacker . 2015. “Forna (Force‐Directed RNA): Simple and Effective Online RNA Secondary Structure Diagrams.” Bioinformatics 31, no. 20: 3377–3379. 26099263 10.1093/bioinformatics/btv372 PMC4595900

[ece371619-bib-0039] Kim, D. , J. M. Paggi , C. Park , C. Bennett , and S. L. Salzberg . 2019. “Graph‐Based Genome Alignment and Genotyping With HISAT2 and HISAT‐Genotype.” Nature Biotechnology 37: 907–915. 10.1038/s41587-019-0201-4. PMC7605509 31375807

[ece371619-bib-0040] Kokot, M. , M. Długosz , and S. Deorowicz . 2017. “KMC 3: Counting and Manipulating k‐Mer Statistics.” Bioinformatics 33: 2759–2761. 10.1093/bioinformatics/btx304. 28472236

[ece371619-bib-0041] Kumar, S. , G. Stecher , M. Li , C. Knyaz , and K. Tamura . 2018. “MEGA X: Molecular Evolutionary Genetics Analysis Across Computing Platforms.” Molecular Biology and Evolution 35: 1547–1549. 10.1093/molbev/msy096. 29722887 PMC5967553

[ece371619-bib-0042] Márquez, E. J. , J. C. Hurtado‐Alarcon , J. P. Isaza , J. F. Alzate , and N. H. Campos . 2016. “Mitochondrial Genome of the Caribbean King Crab *Damithrax spinosissimus* (Lamarck, 1818) (Decapoda: Majidae).” Mitochondrial DNA Part A DNA Mapping, Sequencing, and Analysis 273: 1724–1725. 10.3109/19401736.2014.961140 25242176

[ece371619-bib-0043] Martin, J. W. , and G. E. Davis . 2001. An Updated Classification of the Recent Crustacea. Vol. 39, 1–124. Natural History Museum of L.A. County, Science Series.

[ece371619-bib-0044] Ng, P. K. L. , D. Guinot , and P. J. F. Davie . 2008. “Systema Brachyurorum: Part I. An Annotated Checklist of Extant Brachyuran Crabs of the World.” Raffles Bulletin of Zoology 17: 1–286.

[ece371619-bib-0045] Nguyen, L. T. , H. A. Schmidt , A. Von Haeseler , and B. Q. Minh . 2015. “IQ‐TREE: A Fast and Effective Stochastic Algorithm for Estimating Maximum‐Likelihood Phylogenies.” Molecular Biology and Evolution 32, no. 1: 268–274. 10.1093/molbev/msu300. 25371430 PMC4271533

[ece371619-bib-0046] O'Brien, C. , H. D. Bracken‐Grissom , and J. A. Baeza . 2022. “The Complete Mitochondrial Genome of the Atlantic Ghost Crab *Ocypode quadrata* (Fabricius, 1787) (Brachyura: Ocypodidae: Ocypodinae).” Journal of Crustacean Biology 42, no. 1: p.ruac005. 10.1093/jcbiol/ruac005.

[ece371619-bib-0047] Palopoli, M. F. , S. Minot , D. Pei , A. Satterly , and J. Endrizzi . 2014. “Complete Mitochondrial Genomes of the Human Follicle Mites *Demodex brevis* and *D*. *folliculorum*: Novel Gene Arrangement, Truncated tRNA Genes, and Ancient Divergence Between Species.” BMC Genomics 15: 1–15. 10.1186/1471-2164-15-1124. 25515815 PMC4320518

[ece371619-bib-0048] Polinski, J. M. , T. P. O'Donnell , and A. G. Bodnar . 2025. “Chromosome‐Level Reference Genome for the Jonah Crab, *Cancer borealis* .” G3 15, no. 1: jkae254. 39501747 10.1093/g3journal/jkae254 PMC11708212

[ece371619-bib-0049] Provenzano, A. J. , and W. N. Brownell . 1977. “Larval and Early Post‐Larval Stages of the West Indian Spider Crab, *Mithrax spinosissimus* (Lamarck) (Decapoda: Majidae).” Proceedings of the Biological Society of Washington 90: 735–752.

[ece371619-bib-0050] Rampaul, M. , N. Argenta , J. Bojko , Z. Fazelan , and K. F. Clark . 2024. “Chapter 31. Nimaviruses of Crustaceans.” In Aquaculture Virology, edited by F. S. B. Kibenge and M. G. Godoy , Second ed., 547–575. Academic Press. 10.1016/B978-0-323-91169-6.00016-9.

[ece371619-bib-0051] Rathbun, M. J. 1925. “The Spider Crabs of America.” Bulletin. United States National Museum 129: 1–613.

[ece371619-bib-0052] Sarmashghi, S. , M. Balaban , E. Rachtman , B. Touri , S. Mirarab , and V. Bafna . 2021. “Estimating Repeat Spectra and Genome Length From Low‐Coverage Genome Skims With RESPECT.” PLoS Computational Biology 17: e1009449. 10.1371/journal.pcbi.1009449. 34780468 PMC8629397

[ece371619-bib-0053] Shapiro, J. A. 2005. “A 21st Century View of Evolution: Genome System Architecture, Repetitive DNA, and Natural Genetic Engineering.” Gene 345: 91–100. 15716117 10.1016/j.gene.2004.11.020

[ece371619-bib-0055] Simpson, L. , L. J. Ambrosio , R. Guéron , N. Mora , and D. Owen . 2015. “Reproductive Investment in a Phyletic Giant, the Caribbean King Crab *Damithrax spinosissimus*: Exploring Egg Production Costs in Large Brooding Marine Inertebrates.” Journal of Shellfish Research 34: 1049–1056.

[ece371619-bib-0056] Stothard, P. 2000. “The Sequence Manipulation Suite: JavaScript Programs for Analyzing and Formatting Protein and DNA Sequences.” BioTechniques 28: 1102–1104. 10.2144/00286ir01. 10868275

[ece371619-bib-0057] Tang, B. , D. Zhang , H. Li , et al. 2020. “Chromosome‐Level Genome Assembly Reveals the Unique Genome Evolution of the Swimming Crab (*Portunus trituberculatus*).” GigaScience 9, no. 1: giz161. 10.1093/gigascience/giz161. 31904811 PMC6944217

[ece371619-bib-0058] The Galaxy Community . 2022. “The Galaxy Platform for Accessible, Reproducible and Collaborative Biomedical Analyses: 2022 Update.” Nucleic Acids Research 50, no. W1: W345–W351. 10.1093/nar/gkac247. 35446428 PMC9252830

[ece371619-bib-0059] Turini, T. , J. Colavite , J. A. Bolaños , J. E. Hernández , J. A. Baeza , and W. Santana . 2021. “Larval Development of the Caribbean King Crab *Maguimithrax spinosissimus* (Lamarck, 1818), the Largest Brachyuran in the Western Atlantic (Crustacea: Decapoda: Majoidea).” Journal of the Marine Biological Association of the United Kingdom 101: 577–589.

[ece371619-bib-0060] Wang, D. , Y. Zhang , Z. Zhang , J. Zhu , and J. Yu . 2010. “KaKs_Calculator 2.0: A Toolkit Incorporating Gamma‐Series Methods and Sliding Window Strategies.” Genomics, Proteomics & Bioinformatics 8: 77–80. 10.1016/S1672-0229(10)60008-3. PMC5054116 20451164

[ece371619-bib-0061] Wang, Z. , Z. Xu , H. Chen , Y. Zheng , Z. Wang , and X. Chen . 2024. “Mitogenome Selection Shaped the Terrestrial Adaptation of Grapsidae (Decapoda: Brachyura).” Gene 924: 148594. 38782222 10.1016/j.gene.2024.148594

[ece371619-bib-0062] Werren, J. H. 2011. “Selfish Genetic Elements, Genetic Conflict, and Evolutionary Innovation.” Proceedings of the National Academy of Sciences 108: 10863–10870. 10.1073/pnas.1102343108 PMC3131821 21690392

[ece371619-bib-0063] Williams, A. B. 1984. Shrimps, Lobsters and Crabs of the Atlantic Coast of the Eastern United States, Maine to Florida. Smithsonian Institution Press.

[ece371619-bib-0064] Wood, D. E. , and S. L. Salzberg . 2014. “Kraken: Ultrafast Metagenomic Sequence Classification Using Exact Alignments.” Genome Biology 15: R46. 10.1186/gb-2014-15-3-r46. 24580807 PMC4053813

[ece371619-bib-0065] Zhang, Y. , Y. Yuan , M. Zhang , et al. 2024. “High‐Resolution Chromosome‐Level Genome of *Scylla paramamosain* Provides Molecular Insights Into Adaptive Evolution in Crabs.” BMC Biology 22, no. 1: 255. 39511558 10.1186/s12915-024-02054-1 PMC11545969

[ece371619-bib-0066] Zheng, S. , P. Poczai , J. Hyvönen , J. Tang , and A. Amiryousefi . 2020. “Chloroplot: An Online Program for the Versatile Plotting of Organelle Genomes.” Frontiers in Genetics 11: 576124. 33101394 10.3389/fgene.2020.576124 PMC7545089

