# Supplementary material for: Insight Into the Nuclear and Mitochondrial Genome of the Caribbean King Crab Maguimithrax spinosissimus (Crustacea: Brachyura: Mithracidae) to Support Fisheries Management and Conservation Initiatives
Source: Ecol Evol. 2025 Jun 29;15(7):e71619. doi: 10.1002/ece3.71619 (PMC12206562; doi:10.1002/ece3.71619)
Supplement: Supplementary file 1 — Table S1. Codon usage analysis in the mitochondrial protein‐coding genes of the Caribbean King Crab Maguimithrax spinosissimus. Table S2. Microsatellites in the Control Region of the Caribbean King Crab Maguimithrax spinosissimus detected by the online tool Microsatellite repeats finder. Figure S1. Comparison of SNP differences between the newly assembled mitochondrial genome in this study and a previously published mitochondrial genome of the Caribbean King Crab Maguimithrax spinosissimus. Figure S2. Secondary structure prediction of the control region in the mitochondrial genome of the Caribbean King Crab Maguimithrax spinosissimus. [file ECE3-15-e71619-s001.docx]

**Insight into the nuclear and mitochondrial genome of the Caribbean King Crab *Maguimithrax spinosissimus* (Crustacea: Brachyura: Mithracidae)**

**to support fisheries management and conservation initiatives**

J. Antonio Baeza^1,2,3,*^

1 Department of Biological Sciences, Clemson University, Clemson, SC, USA

2 Departamento de Biología Marina, Universidad Catolica del Norte, Coquimbo, Chile

3 Smithsonian Marine Station at Fort Pierce, Smithsonian Institution, Fort Pierce, FL, USA

*corresponding author. Department of Biological Sciences, Clemson University, Clemson, SC, USA. email: baeza.antonio@gmail.com

**Supplemental Information**

Supplemental Information 1

Table S1. Codon usage analysis in the mitochondrial protein coding genes of the Caribbean King Crab *Maguimithrax spinosissimus*.

Amino Acid Codon Number /1000 Fraction

Ala GCG 5.00 1.35 0.03

Ala GCA 63.00 16.96 0.34

Ala GCT 93.00 25.04 0.49

Ala GCC 27.00 7.27 0.14

Cys TGT 32.00 8.62 0.82

Cys TGC 7.00 1.88 0.18

Asp GAT 51.00 13.73 0.72

Asp GAC 20.00 5.39 0.28

Glu GAG 29.00 7.81 0.34

Glu GAA 57.00 15.35 0.66

Phe TTT 304.00 81.85 0.81

Phe TTC 71.00 19.12 0.19

Gly GGG 35.00 9.42 0.15

Gly GGA 103.00 27.73 0.43

Gly GGT 86.00 23.16 0.36

Gly GGC 14.00 3.77 0.06

His CAT 51.00 13.73 0.64

His CAC 29.00 7.81 0.36

Ile ATT 279.00 75.12 0.86

Ile ATC 45.00 12.12 0.14

Lys AAG 24.00 6.46 0.26

Lys AAA 69.00 18.58 0.74

Leu TTG 46.00 12.39 0.08

Leu TTA 300.00 80.78 0.51

Leu CTG 15.00 4.04 0.03

Leu CTA 81.00 21.81 0.14

Leu CTT 114.00 30.69 0.19

Leu CTC 33.00 8.89 0.06

Met ATG 37.00 9.96 0.19

Met ATA 159.00 42.81 0.81

Asn AAT 97.00 26.12 0.73

Asn AAC 35.00 9.42 0.27

Pro CCG 2.00 0.54 0.01

Pro CCA 34.00 9.15 0.24

Pro CCT 83.00 22.35 0.58

Pro CCC 25.00 6.73 0.17

Gln CAG 10.00 2.69 0.14

Gln CAA 61.00 16.42 0.86

Arg CGG 7.00 1.88 0.13

Arg CGA 34.00 9.15 0.61

Arg CGT 13.00 3.50 0.23

Arg CGC 2.00 0.54 0.04

Ser AGG 34.00 9.15 0.09

Ser AGA 62.00 16.69 0.16

Ser AGT 35.00 9.42 0.09

Ser AGC 7.00 1.88 0.02

Ser TCG 9.00 2.42 0.02

Ser TCA 92.00 24.77 0.24

Ser TCT 103.00 27.73 0.27

Ser TCC 44.00 11.85 0.11

Thr ACG 3.00 0.81 0.02

Thr ACA 62.00 16.69 0.35

Thr ACT 71.00 19.12 0.40

Thr ACC 40.00 10.77 0.23

Val GTG 15.00 4.04 0.07

Val GTA 99.00 26.66 0.44

Val GTT 95.00 25.58 0.42

Val GTC 17.00 4.58 0.08

Trp TGG 15.00 4.04 0.15

Trp TGA 82.00 22.08 0.85

Tyr TAT 101.00 27.19 0.75

Tyr TAC 33.00 8.89 0.25

End TAG 1.00 0.27 0.08

End TAA 12.00 3.23 0.92

Supplemental Information 2

**Table S2.** Microsatellites in the Control Region of the Caribbean King Crab *Maguimithrax spinosissimus*. detected by the online tool Microsatellite repeats finder.

Supplemental Information 3

Figure S1. Comparison of SNP differences between the newly assembled mitochondrial genome in this study and a previously published mitochondrial genome of the Caribbean King Crab *Maguimithrax spinosissimus*.

Supplemental Information 4


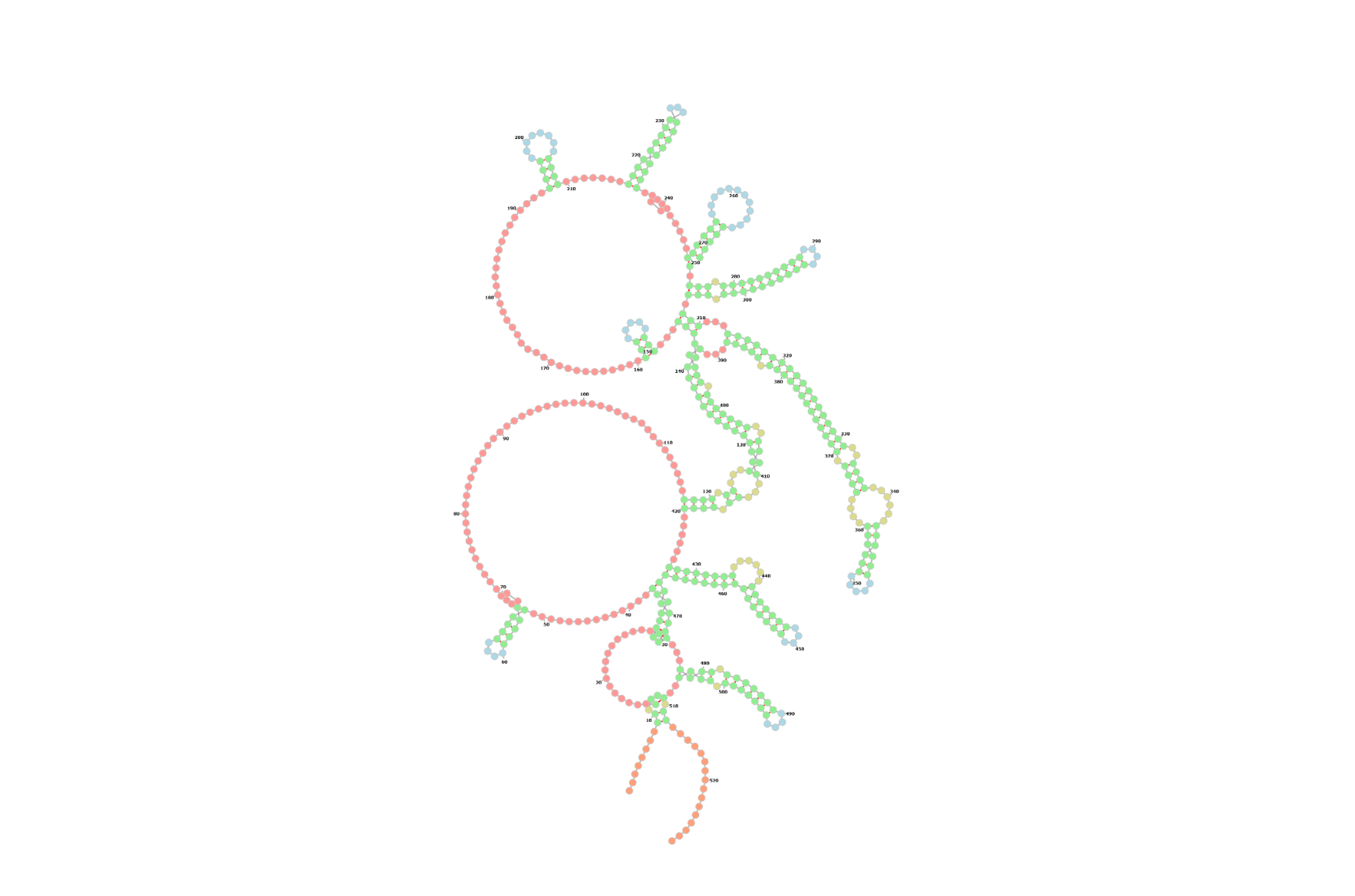


Figure S2. Secondary structure prediction of the control region in the mitochondrial genome of the Caribbean King Crab *Maguimithrax spinosissimus*.

Supplemental Information 5

Primers SSRs

1 ssr-1-1 1 108 58.36 41.67 2.57 ACTGTACTGCTATGATAGGCTACT 57.99 41.67 2.73 TGTAAGTTAGGGGTCTATCGTACT 59 24 166 24

2 ssr-1-1 2 157 58.36 41.67 2.57 ACTGTACTGCTATGATAGGCTACT 59.96 38.46 4.41 ACAGGAGCAGAAAATACATATTGCTG 59 24 215 26

3 ssr-1-1 3 109 58.36 41.67 2.57 ACTGTACTGCTATGATAGGCTACT 58.94 44.0 2.73 CTGTAAGTTAGGGGTCTATCGTACT 59 24 167 25

4 ssr-1-1 4 157 58.36 41.67 2.57 ACTGTACTGCTATGATAGGCTACT 58.87 36.0 3.91 ACAGGAGCAGAAAATACATATTGCT 59 24 215 25

5 ssr-1-1 5 100 58.36 41.67 2.57 ACTGTACTGCTATGATAGGCTACT 57.86 41.67 2.29 AGGGGTCTATCGTACTATGTATGT 59 24 158 24

6 ssr-1-2 1 194 60.04 55.0 2.01 GCACCTGCAGCACACTTTAC 59.86 52.38 3.62 ACAGACCAAGTGACCTGTTCC 17 20 210 21

7 ssr-1-2 2 195 60.18 50.0 1.85 AGCACCTGCAGCACACTTTA 59.86 52.38 3.62 ACAGACCAAGTGACCTGTTCC 16 20 210 21

8 ssr-1-2 3 192 59.82 50.0 3.25 ACCTGCAGCACACTTTACCA 59.86 52.38 3.62 ACAGACCAAGTGACCTGTTCC 19 20 210 21

9 ssr-1-2 4 193 60.04 55.0 2.01 GCACCTGCAGCACACTTTAC 59.58 52.38 3.36 CAGACCAAGTGACCTGTTCCT 17 20 209 21

10 ssr-1-2 5 194 60.18 50.0 1.85 AGCACCTGCAGCACACTTTA 59.58 52.38 3.36 CAGACCAAGTGACCTGTTCCT 16 20 209 21

11 ssr-1-3 1 170 58.48 50.0 2.32 GGTGGACGAGCTCAACAAAT 59.9 60.0 5.14 CCTCCGTATGAAGTGTCGGG 3 20 172 20

12 ssr-1-3 2 167 58.48 50.0 2.32 GGTGGACGAGCTCAACAAAT 60.18 55.0 4.4 CCGTATGAAGTGTCGGGCAT 3 20 169 20

13 ssr-1-3 3 169 58.48 50.0 2.32 GGTGGACGAGCTCAACAAAT 60.53 60.0 6.13 CTCCGTATGAAGTGTCGGGC 3 20 171 20

14 ssr-1-3 4 171 58.48 50.0 2.32 GGTGGACGAGCTCAACAAAT 59.18 55.0 4.79 ACCTCCGTATGAAGTGTCGG 3 20 173 20

15 ssr-1-3 5 167 58.48 50.0 2.32 GGTGGACGAGCTCAACAAAT 59.93 52.38 3.14 CCGTATGAAGTGTCGGGCATA 3 20 169 21

16 ssr-1-4 1 137 58.52 42.86 3.41 TGCTTCGCTCGTAAAAACTGA 60.04 55.0 4.85 AACCGACAGAGTTACGCAGG 26 21 162 20

17 ssr-1-4 2 134 58.52 42.86 3.41 TGCTTCGCTCGTAAAAACTGA 59.76 55.0 4.02 CGACAGAGTTACGCAGGTGA 26 21 159 20

18 ssr-1-4 3 137 59.13 40.91 3.02 TGCTTCGCTCGTAAAAACTGAA 60.04 55.0 4.85 AACCGACAGAGTTACGCAGG 26 22 162 20

19 ssr-1-4 4 138 59.13 40.91 3.41 TTGCTTCGCTCGTAAAAACTGA 60.04 55.0 4.85 AACCGACAGAGTTACGCAGG 25 22 162 20

20 ssr-1-4 5 131 59.09 45.45 5.69 GCTCGTAAAAACTGAATTGGCG 60.04 55.0 4.85 AACCGACAGAGTTACGCAGG 32 22 162 20

21 ssr-1-5 1 121 58.54 50.0 3.51 CTGAAATCTCAGCGGGCTTT 59.18 39.13 5.1 AGGACAACAAATCAGAATTGCGA 23 20 143 23

22 ssr-1-5 2 120 58.54 50.0 3.51 CTGAAATCTCAGCGGGCTTT 57.84 40.91 5.1 GGACAACAAATCAGAATTGCGA 23 20 142 22

23 ssr-1-5 3 121 58.54 50.0 3.51 CTGAAATCTCAGCGGGCTTT 57.84 40.91 4.85 AGGACAACAAATCAGAATTGCG 23 20 143 22

24 ssr-1-5 4 121 59.18 47.62 2.27 CTGAAATCTCAGCGGGCTTTT 59.18 39.13 5.1 AGGACAACAAATCAGAATTGCGA 23 21 143 23

25 ssr-1-5 5 120 58.09 45.0 2.27 TGAAATCTCAGCGGGCTTTT 59.18 39.13 5.1 AGGACAACAAATCAGAATTGCGA 24 20 143 23

26 ssr-1-6 1 139 59.18 47.62 4.4 CCAAATGGTGTATGCTGCTGT 57.76 45.0 3.33 TTGTAGTAAGCACGGCAACA 76 21 214 20

27 ssr-1-6 2 138 59.18 47.62 4.4 CCAAATGGTGTATGCTGCTGT 57.53 45.0 2.71 TGTAGTAAGCACGGCAACAT 76 21 213 20

28 ssr-1-6 3 139 59.77 45.45 3.16 CCAAATGGTGTATGCTGCTGTT 57.76 45.0 3.33 TTGTAGTAAGCACGGCAACA 76 22 214 20

29 ssr-1-6 4 139 59.18 47.62 4.4 CCAAATGGTGTATGCTGCTGT 58.23 42.86 2.71 TTGTAGTAAGCACGGCAACAT 76 21 214 21

30 ssr-1-6 5 138 59.77 45.45 3.16 CCAAATGGTGTATGCTGCTGTT 57.53 45.0 2.71 TGTAGTAAGCACGGCAACAT 76 22 213 20

31 ssr-1-8 1 132 60.11 55.0 3.32 CCTCGGGCAGAATACGTGTT 59.17 55.0 3.51 TGCCTCAGAATAGCCCTGTC 29 20 160 20

32 ssr-1-8 2 134 59.83 55.0 4.49 TTCCTCGGGCAGAATACGTG 59.17 55.0 3.51 TGCCTCAGAATAGCCCTGTC 27 20 160 20

33 ssr-1-8 3 135 59.82 55.0 3.57 GTTCCTCGGGCAGAATACGT 59.17 55.0 3.51 TGCCTCAGAATAGCCCTGTC 26 20 160 20

34 ssr-1-8 4 131 60.11 55.0 3.32 CCTCGGGCAGAATACGTGTT 58.87 55.0 3.41 GCCTCAGAATAGCCCTGTCT 29 20 159 20

35 ssr-1-8 5 133 59.83 55.0 4.49 TTCCTCGGGCAGAATACGTG 58.87 55.0 3.41 GCCTCAGAATAGCCCTGTCT 27 20 159 20

36 ssr-1-10 1 125 60.03 55.0 3.25 GGAAAGGGGGCGAATTGGTA 60.18 55.0 4.35 TCTCCTTGACCCAGACGTGA 22 20 146 20

37 ssr-1-10 2 129 59.89 50.0 2.17 ACAAGGAAAGGGGGCGAATT 60.18 55.0 4.35 TCTCCTTGACCCAGACGTGA 18 20 146 20

38 ssr-1-10 3 130 59.89 50.0 3.34 AACAAGGAAAGGGGGCGAAT 60.18 55.0 4.35 TCTCCTTGACCCAGACGTGA 17 20 146 20

39 ssr-1-10 4 134 60.18 55.0 5.8 AAGGAACAAGGAAAGGGGGC 60.18 55.0 4.35 TCTCCTTGACCCAGACGTGA 13 20 146 20

40 ssr-1-10 5 117 60.18 60.0 4.46 GGCGAATTGGTAGAGGGGAC 60.18 55.0 4.35 TCTCCTTGACCCAGACGTGA 30 20 146 20

41 ssr-1-11 1 135 60.11 50.0 3.53 AGCATCGATTCGGCTTTCCA 58.45 47.83 3.02 AGGTTAGGTTAGGTTAGGCTAGG 13 20 147 23

42 ssr-1-11 2 139 60.11 50.0 3.53 AGCATCGATTCGGCTTTCCA 59.28 45.83 4.58 GGTTAGGTTAGGTTAGGTTAGGCT 13 20 151 24

43 ssr-1-11 3 140 60.11 50.0 3.53 AGCATCGATTCGGCTTTCCA 59.28 45.83 3.93 AGGTTAGGTTAGGTTAGGTTAGGC 13 20 152 24

44 ssr-1-11 4 134 59.69 55.0 3.86 GCATCGATTCGGCTTTCCAG 58.45 47.83 3.02 AGGTTAGGTTAGGTTAGGCTAGG 14 20 147 23

45 ssr-1-11 5 148 59.54 55.0 3.84 TGGTAGACTGCTGAGCATCG 58.45 47.83 3.02 AGGTTAGGTTAGGTTAGGCTAGG 0 20 147 23

46 ssr-1-12 1 180 60.03 45.45 4.18 TCGTCCCATTGTCAAATCGTCT 60.18 55.0 3.67 CGTCCCCTTGTTCCTTGGTT 25 22 204 20

47 ssr-1-12 2 195 60.16 50.0 4.46 CGTCCCATTTTCAAATCGTCCC 60.18 55.0 3.67 CGTCCCCTTGTTCCTTGGTT 10 22 204 20

48 ssr-1-12 3 182 60.03 45.45 4.18 TCGTCCCATTGTCAAATCGTCT 59.67 55.0 3.61 ATCGTCCCCTTGTTCCTTGG 25 22 206 20

49 ssr-1-12 4 197 60.16 50.0 4.46 CGTCCCATTTTCAAATCGTCCC 59.67 55.0 3.61 ATCGTCCCCTTGTTCCTTGG 10 22 206 20

50 ssr-1-12 5 180 58.66 47.62 4.2 TCGTCCCATTGTCAAATCGTC 60.18 55.0 3.67 CGTCCCCTTGTTCCTTGGTT 25 21 204 20

51 ssr-1-13 1 148 58.74 39.13 3.41 TGTTTTCAGTTTCGGTCTTGTCT 60.39 55.0 5.28 TTCCGACTGCATGTTACCGG 57 23 204 20

52 ssr-1-13 2 148 59.67 41.67 3.36 TGTTTTCAGTTTCGGTCTTGTCTC 60.39 55.0 5.28 TTCCGACTGCATGTTACCGG 57 24 204 20

53 ssr-1-13 3 145 57.6 40.91 3.1 TTTCAGTTTCGGTCTTGTCTCT 60.39 55.0 5.28 TTCCGACTGCATGTTACCGG 60 22 204 20

54 ssr-1-13 4 143 58.74 39.13 3.41 TGTTTTCAGTTTCGGTCTTGTCT 60.61 55.0 4.17 ACTGCATGTTACCGGTGTGG 57 23 199 20

55 ssr-1-13 5 145 58.74 39.13 3.41 TGTTTTCAGTTTCGGTCTTGTCT 60.67 55.0 4.16 CGACTGCATGTTACCGGTGT 57 23 201 20

56 ssr-1-14 1 137 59.35 50.0 2.73 TGTCCAGTCAGAGTAGGGTAGT 60.18 50.0 3.32 TGGTGTTGGTATGGCGTTGT 59 22 195 20

57 ssr-1-14 2 138 57.3 50.0 4.34 TTGTCCAGTCAGAGTAGGGT 60.18 50.0 3.32 TGGTGTTGGTATGGCGTTGT 58 20 195 20

58 ssr-1-14 3 151 59.35 50.0 2.73 TGTCCAGTCAGAGTAGGGTAGT 59.46 50.0 4.16 TGCTGCTGATACGATGGTGT 59 22 209 20

59 ssr-1-14 4 152 57.3 50.0 4.34 TTGTCCAGTCAGAGTAGGGT 59.46 50.0 4.16 TGCTGCTGATACGATGGTGT 58 20 209 20

60 ssr-1-14 5 100 57.3 50.0 4.34 TTGTCCAGTCAGAGTAGGGT 59.45 50.0 4.16 ACTGGTGTGGTCATTGTGGT 58 20 157 20

61 ssr-1-15 1 186 60.25 55.0 3.16 TGGCACTCCCGTGTTACTTG 60.0 52.38 2.34 GGCGGACATGCGATTTCTTAC 27 20 212 21

62 ssr-1-15 2 187 59.53 50.0 2.24 TTGGCACTCCCGTGTTACTT 60.0 52.38 2.34 GGCGGACATGCGATTTCTTAC 26 20 212 21

63 ssr-1-15 3 186 60.25 55.0 3.16 TGGCACTCCCGTGTTACTTG 58.7 50.0 2.1 GGCGGACATGCGATTTCTTA 27 20 212 20

64 ssr-1-15 4 184 60.6 55.0 3.91 GCACTCCCGTGTTACTTGCT 60.0 52.38 2.34 GGCGGACATGCGATTTCTTAC 29 20 212 21

65 ssr-1-15 5 188 59.31 50.0 2.24 ATTGGCACTCCCGTGTTACT 60.0 52.38 2.34 GGCGGACATGCGATTTCTTAC 25 20 212 21

66 ssr-1-16 1 111 59.31 45.45 4.24 ACCGCAATATTGGGTTTAGCAG 60.04 50.0 4.84 TGGCTTATCACTGTCGCGTT 56 22 166 20

67 ssr-1-16 2 111 58.27 42.86 3.49 ACCGCAATATTGGGTTTAGCA 60.04 50.0 4.84 TGGCTTATCACTGTCGCGTT 56 21 166 20

68 ssr-1-16 3 107 59.31 45.45 4.24 ACCGCAATATTGGGTTTAGCAG 60.11 50.0 4.93 TTATCACTGTCGCGTTCGCT 56 22 162 20

69 ssr-1-16 4 107 58.27 42.86 3.49 ACCGCAATATTGGGTTTAGCA 60.11 50.0 4.93 TTATCACTGTCGCGTTCGCT 56 21 162 20

70 ssr-1-16 5 108 59.31 45.45 4.24 ACCGCAATATTGGGTTTAGCAG 59.71 55.0 4.7 CTTATCACTGTCGCGTTCGC 56 22 163 20

71 ssr-1-17 1 143 60.18 55.0 4.49 AGGTTTGAAGTCCCCACACG 59.83 55.0 3.58 CTCTATGCACGACCGTGTGA 38 20 180 20

72 ssr-1-17 2 142 60.18 55.0 4.35 GGTTTGAAGTCCCCACACGA 59.83 55.0 3.58 CTCTATGCACGACCGTGTGA 39 20 180 20

73 ssr-1-17 3 138 60.18 55.0 4.49 AGGTTTGAAGTCCCCACACG 59.75 50.0 2.29 TGCACGACCGTGTGATACAT 38 20 175 20

74 ssr-1-17 4 139 60.18 55.0 4.49 AGGTTTGAAGTCCCCACACG 59.75 50.0 2.29 ATGCACGACCGTGTGATACA 38 20 176 20

75 ssr-1-17 5 137 60.18 55.0 4.35 GGTTTGAAGTCCCCACACGA 59.75 50.0 2.29 TGCACGACCGTGTGATACAT 39 20 175 20

76 ssr-1-18 1 189 60.46 60.0 2.43 GCTCTCCGAGTGCTCCTCTA 59.97 50.0 4.11 CCGCCAAAATTCAACGGGTT 28 20 216 20

77 ssr-1-18 2 214 59.87 47.62 3.69 TGCACACTGTTTTGCAAGGAG 59.97 50.0 4.11 CCGCCAAAATTCAACGGGTT 3 21 216 20

78 ssr-1-18 3 139 60.13 47.62 3.72 TCTGCGAGTGTTTGTCTGTGT 59.97 50.0 4.11 CCGCCAAAATTCAACGGGTT 78 21 216 20

79 ssr-1-18 4 213 59.87 47.62 3.85 GCACACTGTTTTGCAAGGAGT 59.97 50.0 4.11 CCGCCAAAATTCAACGGGTT 4 21 216 20

80 ssr-1-18 5 214 58.82 45.0 3.36 TGCACACTGTTTTGCAAGGA 59.97 50.0 4.11 CCGCCAAAATTCAACGGGTT 3 20 216 20

81 ssr-1-19 1 154 59.9 55.0 4.4 GATTTGCGTTCTCCGAGTGC 57.57 37.04 2.73 ACTGATAGTAGTAGTGGTAGTAGTAGT 6 20 159 27

82 ssr-1-19 2 158 60.11 55.0 4.55 GGAGGATTTGCGTTCTCCGA 57.57 37.04 2.73 ACTGATAGTAGTAGTGGTAGTAGTAGT 2 20 159 27

83 ssr-1-19 3 153 60.32 50.0 4.4 ATTTGCGTTCTCCGAGTGCT 57.57 37.04 2.73 ACTGATAGTAGTAGTGGTAGTAGTAGT 7 20 159 27

84 ssr-1-19 4 151 60.53 50.0 3.51 TTGCGTTCTCCGAGTGCTTT 57.57 37.04 2.73 ACTGATAGTAGTAGTGGTAGTAGTAGT 9 20 159 27

85 ssr-1-19 5 152 60.53 50.0 3.91 TTTGCGTTCTCCGAGTGCTT 57.57 37.04 2.73 ACTGATAGTAGTAGTGGTAGTAGTAGT 8 20 159 27

86 ssr-1-20 1 137 57.57 37.04 3.41 ACTACTACTACCACTACTACTATCAGT 59.97 60.0 4.35 GAATCCTAGGAGCGGTCGTG 17 27 153 20

87 ssr-1-20 2 149 57.57 37.04 3.41 ACTACTACTACCACTACTACTATCAGT 59.66 55.0 2.94 TGGACCGACAGGGAATCCTA 17 27 165 20

88 ssr-1-20 3 140 57.57 37.04 3.41 ACTACTACTACCACTACTACTATCAGT 60.47 60.0 4.79 AGGGAATCCTAGGAGCGGTC 17 27 156 20

89 ssr-1-20 4 151 57.57 37.04 3.41 ACTACTACTACCACTACTACTATCAGT 59.45 55.0 3.01 ATTGGACCGACAGGGAATCC 17 27 167 20

90 ssr-1-20 5 150 57.57 37.04 3.41 ACTACTACTACCACTACTACTATCAGT 60.55 55.0 3.24 TTGGACCGACAGGGAATCCT 17 27 166 20

91 ssr-1-21 1 143 59.6 55.0 3.13 GAACGGAAGGTGGGAGGAAA 57.64 42.86 4.49 TCTGTCGTTGTTATGTCGTGT 10 20 152 21

92 ssr-1-21 2 144 60.47 55.0 3.36 AGAACGGAAGGTGGGAGGAA 57.64 42.86 4.49 TCTGTCGTTGTTATGTCGTGT 9 20 152 21

93 ssr-1-21 3 148 59.6 55.0 3.13 GAACGGAAGGTGGGAGGAAA 59.45 47.83 4.35 CCTCATCTGTCGTTGTTATGTCG 10 20 157 23

94 ssr-1-21 4 149 60.47 55.0 3.36 AGAACGGAAGGTGGGAGGAA 59.45 47.83 4.35 CCTCATCTGTCGTTGTTATGTCG 9 20 157 23

95 ssr-1-21 5 145 59.6 55.0 3.13 GAACGGAAGGTGGGAGGAAA 59.33 43.48 4.49 CATCTGTCGTTGTTATGTCGTGT 10 20 154 23

96 ssr-1-24 1 126 58.85 45.45 4.08 GTTTGAGAGACATCCCTTTGCA 59.96 41.67 3.58 CACCTTTACCACGTTGAAATCACA 12 22 137 24

97 ssr-1-24 2 127 58.85 45.45 4.08 GTTTGAGAGACATCCCTTTGCA 59.96 41.67 3.06 ACACCTTTACCACGTTGAAATCAC 12 22 138 24

98 ssr-1-24 3 127 60.18 43.48 4.08 AGTTTGAGAGACATCCCTTTGCA 59.96 41.67 3.58 CACCTTTACCACGTTGAAATCACA 11 23 137 24

99 ssr-1-24 4 128 60.18 43.48 4.08 AGTTTGAGAGACATCCCTTTGCA 59.96 41.67 3.06 ACACCTTTACCACGTTGAAATCAC 11 23 138 24

100 ssr-1-24 5 132 58.85 45.45 4.08 GTTTGAGAGACATCCCTTTGCA 58.88 43.48 3.18 CACTTACACCTTTACCACGTTGA 12 22 143 23

101 ssr-1-25 1 151 58.31 47.62 3.99 CACTTCAGTGCTGGATAACGT 59.08 45.45 3.91 GCAGATTGTTGCTGAAAGATGC 5 21 155 22

102 ssr-1-25 2 151 58.31 47.62 3.99 CACTTCAGTGCTGGATAACGT 60.37 43.48 3.79 GCAGATTGTTGCTGAAAGATGCT 5 21 155 23

103 ssr-1-25 3 110 58.85 45.45 4.52 TGTGACAAGTACCATTCTTGCC 59.08 45.45 3.91 GCAGATTGTTGCTGAAAGATGC 46 22 155 22

104 ssr-1-25 4 110 60.18 43.48 4.75 TGTGACAAGTACCATTCTTGCCT 59.08 45.45 3.91 GCAGATTGTTGCTGAAAGATGC 46 23 155 22

105 ssr-1-25 5 152 58.31 47.62 3.99 CACTTCAGTGCTGGATAACGT 60.61 43.48 3.91 TGCAGATTGTTGCTGAAAGATGC 5 21 156 23

106 ssr-1-26 1 172 59.9 60.0 3.35 CGCTAAGCAGGAGGTCAGAG 60.11 60.0 3.67 CCACTCATCCGACCAGTGTC 31 20 202 20

107 ssr-1-26 2 167 59.89 55.0 3.55 AGCAGGAGGTCAGAGACAGT 60.11 60.0 3.67 CCACTCATCCGACCAGTGTC 36 20 202 20

108 ssr-1-26 3 128 59.9 60.0 3.35 CGCTAAGCAGGAGGTCAGAG 60.25 50.0 3.18 TGCCATTCAGCCCAACGTTA 31 20 158 20

109 ssr-1-26 4 174 59.75 55.0 3.51 AACGCTAAGCAGGAGGTCAG 60.11 60.0 3.67 CCACTCATCCGACCAGTGTC 29 20 202 20

110 ssr-1-26 5 123 59.89 55.0 3.55 AGCAGGAGGTCAGAGACAGT 60.25 50.0 3.18 TGCCATTCAGCCCAACGTTA 36 20 158 20

111 ssr-1-27 1 113 59.17 50.0 4.26 TCAGTGTGGTGAGTTGAGCA 59.69 50.0 3.16 ATGTGTCGTGCAGCAACAAG 77 20 189 20

112 ssr-1-27 2 111 59.17 50.0 4.4 AGTGTGGTGAGTTGAGCACT 59.69 50.0 3.16 ATGTGTCGTGCAGCAACAAG 79 20 189 20

113 ssr-1-27 3 112 59.06 55.0 4.4 CAGTGTGGTGAGTTGAGCAC 59.69 50.0 3.16 ATGTGTCGTGCAGCAACAAG 78 20 189 20

114 ssr-1-27 4 179 58.94 50.0 3.1 AGCAGCAAAGGACTGTCTCT 59.69 50.0 3.16 ATGTGTCGTGCAGCAACAAG 11 20 189 20

115 ssr-1-27 5 184 60.13 47.62 3.55 AGTGAAGCAGCAAAGGACTGT 59.69 50.0 3.16 ATGTGTCGTGCAGCAACAAG 6 21 189 20

116 ssr-1-28 1 140 60.1 55.0 4.37 TTCCTCTTCCCAGGTTCCCA 60.1 55.0 4.0 AACCTCACCTCACCTCACCT 42 20 181 20

117 ssr-1-28 2 141 59.67 60.0 3.97 GTTCCTCTTCCCAGGTTCCC 60.1 55.0 4.0 AACCTCACCTCACCTCACCT 41 20 181 20

118 ssr-1-28 3 136 60.4 55.0 3.5 TCTTCCCAGGTTCCCAGGTT 60.1 55.0 4.0 AACCTCACCTCACCTCACCT 46 20 181 20

119 ssr-1-28 4 132 59.59 55.0 2.57 CCCAGGTTCCCAGGTTTCAT 60.1 55.0 4.0 AACCTCACCTCACCTCACCT 50 20 181 20

120 ssr-1-28 5 134 59.52 55.0 2.78 TTCCCAGGTTCCCAGGTTTC 60.1 55.0 4.0 AACCTCACCTCACCTCACCT 48 20 181 20

121 ssr-1-29 1 191 60.1 55.0 4.37 TTCCTCTTCCCAGGTTCCCA 59.28 40.0 3.5 ACCTAATCTCACGTAAACCTAACCT 22 20 212 25

122 ssr-1-29 2 196 60.1 55.0 4.37 TTCCTCTTCCCAGGTTCCCA 59.28 40.0 3.5 ACCTAACCTAATCTCACGTAAACCT 22 20 217 25

123 ssr-1-29 3 192 59.67 60.0 3.97 GTTCCTCTTCCCAGGTTCCC 59.28 40.0 3.5 ACCTAATCTCACGTAAACCTAACCT 21 20 212 25

124 ssr-1-29 4 197 59.67 60.0 3.97 GTTCCTCTTCCCAGGTTCCC 59.28 40.0 3.5 ACCTAACCTAATCTCACGTAAACCT 21 20 217 25

125 ssr-1-29 5 191 60.1 55.0 4.37 TTCCTCTTCCCAGGTTCCCA 58.03 41.67 2.85 ACCTAATCTCACGTAAACCTAACC 22 20 212 24

126 ssr-1-30 1 159 60.1 55.0 3.5 AGGTGAGGTGAGGTGAGGTT 60.03 60.0 4.85 TACTCTCCTCCTTCCCAGGC 19 20 177 20

127 ssr-1-30 2 147 60.1 55.0 3.5 AGGTGAGGTGAGGTGAGGTT 59.96 55.0 2.85 TCCCAGGCCAGATCAGGTTA 19 20 165 20

128 ssr-1-30 3 209 60.1 55.0 3.5 AGGTGAGGTGAGGTGAGGTT 59.61 55.0 2.32 GGCTTGACCCGCCATATTTG 19 20 227 20

129 ssr-1-30 4 161 60.54 60.0 3.86 TGAGGTGAGGTGAGGTGAGG 60.03 60.0 4.85 TACTCTCCTCCTTCCCAGGC 17 20 177 20

130 ssr-1-30 5 166 60.54 60.0 3.86 TGAGGTGAGGTGAGGTGAGG 60.03 60.0 4.85 TACTCTCCTCCTTCCCAGGC 12 20 177 20

131 ssr-1-32 1 114 57.37 42.86 4.3 AAGAAGCATCCATGTACCGAA 59.7 45.45 3.41 TCGAAGAGCATGAACCTTCACT 33 21 146 22

132 ssr-1-32 2 117 59.3 43.48 4.69 ACTAAGAAGCATCCATGTACCGA 59.7 45.45 3.41 TCGAAGAGCATGAACCTTCACT 30 23 146 22

133 ssr-1-32 3 113 57.15 42.86 3.34 AGAAGCATCCATGTACCGAAT 59.7 45.45 3.41 TCGAAGAGCATGAACCTTCACT 34 21 146 22

134 ssr-1-32 4 110 58.12 40.91 2.69 AGCATCCATGTACCGAATTTCA 59.7 45.45 3.41 TCGAAGAGCATGAACCTTCACT 37 22 146 22

135 ssr-1-32 5 113 57.37 42.86 4.3 AAGAAGCATCCATGTACCGAA 59.33 50.0 3.51 CGAAGAGCATGAACCTTCACTC 33 21 145 22

136 ssr-1-33 1 135 57.76 40.91 3.02 TGGGTTCTTTCTTCAACCAAGA 59.79 47.62 3.96 TCCGCCAGGAAGAATAATGCA 18 22 152 21

137 ssr-1-33 2 118 57.76 40.91 3.02 TGGGTTCTTTCTTCAACCAAGA 58.71 50.0 2.59 TGCAATGATCGCCGTTACTC 18 22 135 20

138 ssr-1-33 3 195 57.76 40.91 3.02 TGGGTTCTTTCTTCAACCAAGA 59.58 47.62 5.68 TCCAGTTTCTCTCTTTGCCGT 18 22 212 21

139 ssr-1-33 4 134 57.76 40.91 3.02 TGGGTTCTTTCTTCAACCAAGA 58.32 50.0 3.96 CCGCCAGGAAGAATAATGCA 18 22 151 20

140 ssr-1-33 5 136 58.2 39.13 3.02 ATGGGTTCTTTCTTCAACCAAGA 59.79 47.62 3.96 TCCGCCAGGAAGAATAATGCA 17 23 152 21

141 ssr-1-34 1 126 59.3 50.0 2.69 AGGGGCTACATTGGCTTCAA 60.1 60.0 4.4 TAGGGAAGGAGAGGGATGGC 77 20 202 20

142 ssr-1-34 2 119 59.3 50.0 2.69 AGGGGCTACATTGGCTTCAA 59.89 60.0 2.9 GGAGAGGGATGGCCAATCAG 77 20 195 20

143 ssr-1-34 3 120 59.3 50.0 2.69 AGGGGCTACATTGGCTTCAA 60.33 55.0 2.57 AGGAGAGGGATGGCCAATCA 77 20 196 20

144 ssr-1-34 4 203 59.03 50.0 2.32 TGCTTGGAACCCGGAAATTG 60.1 60.0 4.4 TAGGGAAGGAGAGGGATGGC 0 20 202 20

145 ssr-1-34 5 196 59.03 50.0 2.32 TGCTTGGAACCCGGAAATTG 59.89 60.0 2.9 GGAGAGGGATGGCCAATCAG 0 20 195 20

146 ssr-1-35 1 188 59.96 55.0 5.14 TTGAAGGGTGATGGTTCGGG 60.11 60.0 4.45 CACTTGGGGCTATGTCTGGG 13 20 200 20

147 ssr-1-35 2 172 59.89 55.0 2.45 CGGGAGCTCTGCAAGATGAT 60.11 60.0 4.45 CACTTGGGGCTATGTCTGGG 29 20 200 20

148 ssr-1-35 3 186 60.11 60.0 4.3 GAAGGGTGATGGTTCGGGAG 60.11 60.0 4.45 CACTTGGGGCTATGTCTGGG 15 20 200 20

149 ssr-1-35 4 175 60.11 55.0 2.4 GTTCGGGAGCTCTGCAAGAT 60.11 60.0 4.45 CACTTGGGGCTATGTCTGGG 26 20 200 20

150 ssr-1-35 5 174 60.11 55.0 2.9 TTCGGGAGCTCTGCAAGATG 60.11 60.0 4.45 CACTTGGGGCTATGTCTGGG 27 20 200 20

151 ssr-1-37 1 107 59.3 50.0 3.41 TTACTTGGCCTGGCATCAGT 59.36 52.38 4.0 GGGGAAATTTAGGGGACTGGT 44 20 150 21

152 ssr-1-37 2 112 59.3 50.0 4.92 AGGATTTACTTGGCCTGGCA 59.36 52.38 4.0 GGGGAAATTTAGGGGACTGGT 39 20 150 21

153 ssr-1-37 3 105 59.08 50.0 2.12 ACTTGGCCTGGCATCAGTAT 59.36 52.38 4.0 GGGGAAATTTAGGGGACTGGT 46 20 150 21

154 ssr-1-37 4 108 59.3 50.0 3.41 TTACTTGGCCTGGCATCAGT 59.07 52.38 4.0 AGGGGAAATTTAGGGGACTGG 44 20 151 21

155 ssr-1-37 5 113 59.3 50.0 4.92 AGGATTTACTTGGCCTGGCA 59.07 52.38 4.0 AGGGGAAATTTAGGGGACTGG 39 20 151 21

156 ssr-1-38 1 135 59.38 47.62 4.58 AGTGAAACGATGAGTCAGGCT 57.62 40.91 3.58 TTTTCTAAGCCTTCCCATGACA 23 21 157 22

157 ssr-1-38 2 133 59.38 47.62 4.58 AGTGAAACGATGAGTCAGGCT 57.41 40.91 2.71 TTCTAAGCCTTCCCATGACATT 23 21 155 22

158 ssr-1-38 3 134 59.38 47.62 4.58 AGTGAAACGATGAGTCAGGCT 57.41 40.91 3.06 TTTCTAAGCCTTCCCATGACAT 23 21 156 22

159 ssr-1-38 4 123 59.09 47.62 4.75 AGTCAGGCTTTATCACTGGCT 57.62 40.91 3.58 TTTTCTAAGCCTTCCCATGACA 35 21 157 22

160 ssr-1-38 5 128 59.38 47.62 4.58 AGTGAAACGATGAGTCAGGCT 58.32 39.13 2.66 AGCCTTCCCATGACATTAAAACT 23 21 150 23

161 ssr-1-39 1 182 57.03 26.92 3.16 TTCGAAAACTAATCACAAAAACTTGT 57.61 45.45 3.71 AGGAGTACTGAGACAAAGGAGA 20 26 201 22

162 ssr-1-39 2 182 57.03 26.92 3.16 TTCGAAAACTAATCACAAAAACTTGT 58.26 43.48 2.87 AGGAGTACTGAGACAAAGGAGAA 20 26 201 23

163 ssr-1-39 3 183 57.03 26.92 3.16 TTCGAAAACTAATCACAAAAACTTGT 58.26 43.48 3.71 AAGGAGTACTGAGACAAAGGAGA 20 26 202 23

164 ssr-1-39 4 183 57.59 25.93 3.16 TTTCGAAAACTAATCACAAAAACTTGT 57.61 45.45 3.71 AGGAGTACTGAGACAAAGGAGA 19 27 201 22

165 ssr-1-39 5 181 57.03 26.92 3.16 TTCGAAAACTAATCACAAAAACTTGT 59.99 44.0 2.66 GGAGTACTGAGACAAAGGAGAAACT 20 26 200 25

166 ssr-1-40 1 181 59.49 55.0 3.67 GGCGTGATGTGTCTTGTCAC 60.25 55.0 3.58 CCAACATTGCCCCTCTGTGA 12 20 192 20

167 ssr-1-40 2 182 60.6 55.0 3.58 GGGCGTGATGTGTCTTGTCA 60.25 55.0 3.58 CCAACATTGCCCCTCTGTGA 11 20 192 20

168 ssr-1-40 3 175 59.49 55.0 3.67 GGCGTGATGTGTCTTGTCAC 59.23 50.0 3.58 TTGCCCCTCTGTGAATCACA 12 20 186 20

169 ssr-1-40 4 176 60.6 55.0 3.58 GGGCGTGATGTGTCTTGTCA 59.23 50.0 3.58 TTGCCCCTCTGTGAATCACA 11 20 186 20

170 ssr-1-40 5 112 60.15 57.89 4.02 GCTAGCCAATCAGCGTCCA 60.25 55.0 3.58 CCAACATTGCCCCTCTGTGA 81 19 192 20

171 ssr-1-41 1 137 59.93 47.83 3.67 GGTTAGGTCAGGTTAGGTTTGGT 57.65 40.91 3.74 TGCACTCTACTAAACCAAGCTT 75 23 211 22

172 ssr-1-41 2 138 59.93 47.83 3.67 GGTTAGGTCAGGTTAGGTTTGGT 57.44 40.91 3.74 ATGCACTCTACTAAACCAAGCT 75 23 212 22

173 ssr-1-41 3 138 59.67 47.83 3.28 AGGTTAGGTCAGGTTAGGTTTGG 57.65 40.91 3.74 TGCACTCTACTAAACCAAGCTT 74 23 211 22

174 ssr-1-41 4 139 59.67 47.83 3.28 AGGTTAGGTCAGGTTAGGTTTGG 57.44 40.91 3.74 ATGCACTCTACTAAACCAAGCT 74 23 212 22

175 ssr-1-41 5 138 59.93 47.83 3.67 GGTTAGGTCAGGTTAGGTTTGGT 58.09 39.13 3.74 ATGCACTCTACTAAACCAAGCTT 75 23 212 23

176 ssr-1-43 1 207 59.96 55.0 2.4 CCAGTCCCACCTTGCATCTT 60.01 57.89 5.01 CACAAGCACACCATGGCAC 5 20 211 19

177 ssr-1-43 2 209 60.25 55.0 3.91 AACCAGTCCCACCTTGCATC 60.01 57.89 5.01 CACAAGCACACCATGGCAC 3 20 211 19

178 ssr-1-43 3 210 60.25 55.0 3.96 GAACCAGTCCCACCTTGCAT 60.01 57.89 5.01 CACAAGCACACCATGGCAC 2 20 211 19

179 ssr-1-43 4 212 60.61 60.0 4.01 GAGAACCAGTCCCACCTTGC 60.01 57.89 5.01 CACAAGCACACCATGGCAC 0 20 211 19

180 ssr-1-43 5 205 58.93 50.0 3.02 AGTCCCACCTTGCATCTTCA 60.01 57.89 5.01 CACAAGCACACCATGGCAC 7 20 211 19

181 ssr-1-44 1 190 58.82 43.48 2.15 GTCACACACGATCACCATTATCA 58.51 36.0 3.71 ACGGTTATGGGAAAAATAAAGAGGA 14 23 203 25

182 ssr-1-44 2 191 58.82 43.48 2.15 GTCACACACGATCACCATTATCA 59.4 38.46 3.71 GACGGTTATGGGAAAAATAAAGAGGA 14 23 204 26

183 ssr-1-44 3 192 58.82 43.48 2.15 GTCACACACGATCACCATTATCA 59.4 38.46 3.69 AGACGGTTATGGGAAAAATAAAGAGG 14 23 205 26

184 ssr-1-44 4 190 58.82 43.48 2.15 GTCACACACGATCACCATTATCA 57.23 37.5 3.69 ACGGTTATGGGAAAAATAAAGAGG 14 23 203 24

185 ssr-1-44 5 189 57.54 40.91 2.15 TCACACACGATCACCATTATCA 58.51 36.0 3.71 ACGGTTATGGGAAAAATAAAGAGGA 15 22 203 25

186 ssr-1-46 1 190 60.03 55.0 2.94 TCCGCGTCTCCTTTCTCCTA 59.76 55.0 6.13 CTTGCTGTTCTCTTTGCGGG 4 20 193 20

187 ssr-1-46 2 163 60.03 55.0 2.94 TCCGCGTCTCCTTTCTCCTA 59.75 55.0 2.24 GTCTCGGCGGACCTTTTACT 4 20 166 20

188 ssr-1-46 3 222 60.03 55.0 2.94 TCCGCGTCTCCTTTCTCCTA 60.25 55.0 3.16 TTGGCCCTCCCGTTTAGTTG 4 20 225 20

189 ssr-1-46 4 220 60.03 55.0 2.94 TCCGCGTCTCCTTTCTCCTA 60.25 55.0 2.83 GGCCCTCCCGTTTAGTTGTT 4 20 223 20

190 ssr-1-46 5 230 60.03 55.0 2.94 TCCGCGTCTCCTTTCTCCTA 60.32 60.0 5.14 GCACTATATTGGCCCTCCCG 4 20 233 20

191 ssr-1-47 1 135 57.84 34.78 4.2 TGTTTCTTGTTTTGCTTTTCCCT 58.87 60.0 4.3 GGTGGTAGGAAGATGGGAGG 5 23 139 20

192 ssr-1-47 2 136 57.84 34.78 4.2 TGTTTCTTGTTTTGCTTTTCCCT 60.34 57.14 4.3 AGGTGGTAGGAAGATGGGAGG 5 23 140 21

193 ssr-1-47 3 138 57.84 34.78 4.2 TGTTTCTTGTTTTGCTTTTCCCT 59.64 52.38 4.37 TGAGGTGGTAGGAAGATGGGA 5 23 142 21

194 ssr-1-47 4 132 57.84 34.78 4.2 TGTTTCTTGTTTTGCTTTTCCCT 59.59 57.14 3.95 GGTAGGAAGATGGGAGGTTCG 5 23 136 21

195 ssr-1-47 5 139 57.84 34.78 4.2 TGTTTCTTGTTTTGCTTTTCCCT 59.51 57.14 4.0 GTGAGGTGGTAGGAAGATGGG 5 23 143 21

196 ssr-1-48 1 145 59.11 50.0 4.26 AGGAAAAGCATTGCCTCAGC 57.84 45.0 3.16 AACCTCCACCAAAAGCTGTT 47 20 191 20

197 ssr-1-48 2 146 59.11 50.0 4.26 AGGAAAAGCATTGCCTCAGC 57.84 45.0 4.4 AAACCTCCACCAAAAGCTGT 47 20 192 20

198 ssr-1-48 3 146 59.11 50.0 4.26 AGGAAAAGCATTGCCTCAGC 58.53 42.86 3.16 AAACCTCCACCAAAAGCTGTT 47 20 192 21

199 ssr-1-48 4 147 59.11 50.0 4.26 AGGAAAAGCATTGCCTCAGC 58.53 42.86 4.4 AAAACCTCCACCAAAAGCTGT 47 20 193 21

200 ssr-1-48 5 146 59.73 47.62 4.26 AAGGAAAAGCATTGCCTCAGC 57.84 45.0 3.16 AACCTCCACCAAAAGCTGTT 46 21 191 20

201 ssr-1-49 1 140 59.97 60.0 4.63 GGAACGTGAGATAGGCGGAG 59.96 55.0 4.17 CATCATCACCACCACCACCA 57 20 196 20

202 ssr-1-49 2 142 59.97 60.0 6.13 GAGGAACGTGAGATAGGCGG 59.96 55.0 4.17 CATCATCACCACCACCACCA 55 20 196 20

203 ssr-1-49 3 139 59.97 60.0 4.63 GGAACGTGAGATAGGCGGAG 59.96 55.0 4.16 ATCATCACCACCACCACCAC 57 20 195 20

204 ssr-1-49 4 141 59.97 60.0 6.13 GAGGAACGTGAGATAGGCGG 59.96 55.0 4.16 ATCATCACCACCACCACCAC 55 20 195 20

205 ssr-1-49 5 141 59.97 60.0 4.63 GGAACGTGAGATAGGCGGAG 59.67 55.0 4.61 TCATCATCACCACCACCACC 57 20 197 20

206 ssr-1-50 1 227 59.97 60.0 4.63 GGAACGTGAGATAGGCGGAG 59.84 41.67 3.69 TGTTGTTGTCCTCTTTCTTCCTCT 42 20 268 24

207 ssr-1-50 2 229 59.97 60.0 6.13 GAGGAACGTGAGATAGGCGG 59.84 41.67 3.69 TGTTGTTGTCCTCTTTCTTCCTCT 40 20 268 24

208 ssr-1-50 3 227 59.97 60.0 4.63 GGAACGTGAGATAGGCGGAG 58.55 43.48 3.71 TGTTGTTGTCCTCTTTCTTCCTC 42 20 268 23

209 ssr-1-50 4 229 59.97 60.0 6.13 GAGGAACGTGAGATAGGCGG 58.55 43.48 3.71 TGTTGTTGTCCTCTTTCTTCCTC 40 20 268 23

210 ssr-1-50 5 227 59.97 60.0 4.63 GGAACGTGAGATAGGCGGAG 57.5 40.91 3.36 TGTTGTTGTCCTCTTTCTTCCT 42 20 268 22

211 ssr-1-51 1 226 60.0 57.14 4.17 GTAGTAGTGGTGGTGGTGGTG 60.11 60.0 3.36 CAAAGAGGACGGACGGAGAC 17 21 242 20

212 ssr-1-51 2 223 59.93 63.16 4.61 GTAGTGGTGGTGGTGGTGG 60.11 60.0 3.36 CAAAGAGGACGGACGGAGAC 20 19 242 20

213 ssr-1-51 3 227 60.13 52.38 4.16 AGTAGTAGTGGTGGTGGTGGT 60.11 60.0 3.36 CAAAGAGGACGGACGGAGAC 16 21 242 20

214 ssr-1-51 4 225 58.66 55.0 4.17 TAGTAGTGGTGGTGGTGGTG 60.11 60.0 3.36 CAAAGAGGACGGACGGAGAC 18 20 242 20

215 ssr-1-51 5 226 58.65 55.0 4.16 GTAGTAGTGGTGGTGGTGGT 60.11 60.0 3.36 CAAAGAGGACGGACGGAGAC 17 20 242 20

216 ssr-1-52 1 153 58.75 47.62 3.58 AGGTGAGAGAACGGAATCACA 59.64 50.0 3.51 TGTCTGTCTGTCTGTGTCTGTC 4 21 156 22

217 ssr-1-52 2 157 58.75 47.62 3.58 AGGTGAGAGAACGGAATCACA 59.64 50.0 3.67 TGTCTGTCTGTCTGTCTGTGTC 4 21 160 22

218 ssr-1-52 3 154 58.75 47.62 3.58 AGGTGAGAGAACGGAATCACA 59.64 50.0 3.41 CTGTCTGTCTGTCTGTGTCTGT 4 21 157 22

219 ssr-1-52 4 158 58.75 47.62 3.58 AGGTGAGAGAACGGAATCACA 59.64 50.0 3.72 CTGTCTGTCTGTCTGTCTGTGT 4 21 161 22

220 ssr-1-52 5 153 58.75 47.62 3.58 AGGTGAGAGAACGGAATCACA 58.62 47.62 3.41 TGTCTGTCTGTCTGTGTCTGT 4 21 156 21

221 ssr-1-53 1 221 58.85 45.45 4.17 GTCCTGTAAGCACATTTCACCA 59.2 55.0 3.36 TCCCTCCTTCCTTCCTTCCT 5 22 225 20

222 ssr-1-53 2 222 60.18 43.48 4.17 AGTCCTGTAAGCACATTTCACCA 59.2 55.0 3.36 TCCCTCCTTCCTTCCTTCCT 4 23 225 20

223 ssr-1-53 3 222 58.59 45.45 4.02 AGTCCTGTAAGCACATTTCACC 59.2 55.0 3.36 TCCCTCCTTCCTTCCTTCCT 4 22 225 20

224 ssr-1-53 4 221 58.85 45.45 4.17 GTCCTGTAAGCACATTTCACCA 59.85 52.38 3.36 TCCCTCCTTCCTTCCTTCCTT 5 22 225 21

225 ssr-1-53 5 222 58.85 45.45 4.17 GTCCTGTAAGCACATTTCACCA 59.85 52.38 3.36 TTCCCTCCTTCCTTCCTTCCT 5 22 226 21

226 ssr-1-54 1 137 59.38 50.0 3.41 CAGACAGACAGACAGACAGACA 60.25 60.0 3.97 TCCTTCCCTCCTTCCTTCCC 1 22 137 20

227 ssr-1-54 2 136 59.38 50.0 3.41 CAGACAGACAGACAGACAGACA 60.25 60.0 4.2 CCTTCCCTCCTTCCTTCCCT 1 22 136 20

228 ssr-1-54 3 138 59.38 50.0 3.51 ACAGACAGACAGACAGACAGAC 60.25 60.0 3.97 TCCTTCCCTCCTTCCTTCCC 0 22 137 20

229 ssr-1-54 4 137 59.38 50.0 3.51 ACAGACAGACAGACAGACAGAC 60.25 60.0 4.2 CCTTCCCTCCTTCCTTCCCT 0 22 136 20

230 ssr-1-54 5 135 59.12 50.0 3.41 GACAGACAGACAGACAGACAGA 60.25 60.0 3.97 TCCTTCCCTCCTTCCTTCCC 3 22 137 20

231 ssr-1-55 1 149 60.03 50.0 3.41 CACATCAGGTGGATCACAGTCA 60.11 55.0 2.59 TCGCCCGGACACTTTTATCC 77 22 225 20

232 ssr-1-55 2 152 60.03 50.0 3.41 CACATCAGGTGGATCACAGTCA 60.11 55.0 1.52 GGATCGCCCGGACACTTTTA 77 22 228 20

233 ssr-1-55 3 148 58.74 47.62 3.41 ACATCAGGTGGATCACAGTCA 60.11 55.0 2.59 TCGCCCGGACACTTTTATCC 78 21 225 20

234 ssr-1-55 4 151 58.74 47.62 3.41 ACATCAGGTGGATCACAGTCA 60.11 55.0 1.52 GGATCGCCCGGACACTTTTA 78 21 228 20

235 ssr-1-55 5 148 60.03 50.0 3.41 CACATCAGGTGGATCACAGTCA 60.39 55.0 3.41 CGCCCGGACACTTTTATCCA 77 22 224 20

236 ssr-1-56 1 146 60.11 65.0 4.3 CCTCCTCCTCCTCTTCCTCC 59.83 55.0 4.17 GTGTGTGTGTGTGTGTGTGG 28 20 173 20

237 ssr-1-56 2 157 60.11 65.0 4.3 CCTCCTCCTCCTCTTCCTCC 59.83 55.0 3.82 GGTGTGTGTGTGTGTGTGTG 28 20 184 20

238 ssr-1-56 3 164 60.11 65.0 4.3 CCTCCTCCTCCTCTTCCTCC 60.25 50.0 3.72 TTGTGTGGGTGTGTGTGTGT 28 20 191 20

239 ssr-1-56 4 135 60.11 65.0 4.3 CCTCCTCCTCCTCTTCCTCC 59.61 50.0 1.89 TGTGTGTGGGCGACAGTTAT 28 20 162 20

240 ssr-1-56 5 156 59.66 60.0 3.71 TCTCTTCCCACCTCCTCCTC 59.83 55.0 4.17 GTGTGTGTGTGTGTGTGTGG 18 20 173 20

241 ssr-1-57 1 147 59.66 60.0 3.71 TGTTCCTCCTCCTCCTCCTC 60.03 60.0 3.46 TGAGGATGGAGGAGGGAAGG 42 20 188 20

242 ssr-1-57 2 155 59.66 60.0 3.71 TGTTCCTCCTCCTCCTCCTC 60.03 60.0 4.3 GGGGTGTTTGAGGATGGAGG 42 20 196 20

243 ssr-1-57 3 156 59.66 60.0 3.71 TGTTCCTCCTCCTCCTCCTC 60.03 60.0 3.86 GGGGGTGTTTGAGGATGGAG 42 20 197 20

244 ssr-1-57 4 146 60.4 65.0 4.3 GTTCCTCCTCCTCCTCCTCC 60.03 60.0 3.46 TGAGGATGGAGGAGGGAAGG 43 20 188 20

245 ssr-1-57 5 154 60.4 65.0 4.3 GTTCCTCCTCCTCCTCCTCC 60.03 60.0 4.3 GGGGTGTTTGAGGATGGAGG 43 20 196 20

246 ssr-1-59 1 117 60.81 55.0 5.69 TGCGTCAGATATTGGTGGCG 60.11 60.0 4.63 TCCTCCTCCTGATTCCTCGG 78 20 194 20

247 ssr-1-59 2 124 60.81 55.0 5.69 TGCGTCAGATATTGGTGGCG 59.74 60.0 2.9 CACCTCCTCCTCCTCCTGAT 78 20 201 20

248 ssr-1-59 3 116 59.79 52.38 2.69 AGCCACCACTAAGCCATTAGG 60.03 60.0 4.04 TTACCTCCCCTTCCCTCTCG 39 21 154 20

249 ssr-1-59 4 134 59.79 52.38 2.69 AGCCACCACTAAGCCATTAGG 59.97 55.0 2.85 CGGTGACACTCCTTGCTCTT 39 21 172 20

250 ssr-1-59 5 156 59.79 52.38 2.69 AGCCACCACTAAGCCATTAGG 60.11 60.0 4.63 TCCTCCTCCTGATTCCTCGG 39 21 194 20

251 ssr-1-60 1 147 59.97 60.0 4.85 GTAGCGTATCTCACCCTGGC 60.03 60.0 3.66 TCTCCTCCTCACCCTCCATG 31 20 177 20

252 ssr-1-60 2 128 60.03 60.0 2.85 CGAGAGGGAAGGGGAGGTAA 60.03 60.0 3.66 TCTCCTCCTCACCCTCCATG 50 20 177 20

253 ssr-1-60 3 110 59.97 55.0 4.94 AAGAGCAAGGAGTGTCACCG 60.03 60.0 3.66 TCTCCTCCTCACCCTCCATG 68 20 177 20

254 ssr-1-60 4 162 60.04 60.0 3.58 GTGGTGGTGGTGGTAGTAGC 60.03 60.0 3.66 TCTCCTCCTCACCCTCCATG 16 20 177 20

255 ssr-1-60 5 143 59.97 60.0 4.85 GTAGCGTATCTCACCCTGGC 60.11 60.0 4.01 CTCCTCACCCTCCATGCTTG 31 20 173 20

256 ssr-1-61 1 103 60.04 55.0 2.73 GGTCGCTCAGCAACAGTAGT 59.54 50.0 4.17 AGCATGGCATTAGCAGTTGC 48 20 150 20

257 ssr-1-61 2 104 59.76 55.0 2.57 AGGTCGCTCAGCAACAGTAG 59.54 50.0 4.17 AGCATGGCATTAGCAGTTGC 47 20 150 20

258 ssr-1-61 3 105 59.32 50.0 2.74 AAGGTCGCTCAGCAACAGTA 59.54 50.0 4.17 AGCATGGCATTAGCAGTTGC 46 20 150 20

259 ssr-1-61 4 118 60.04 55.0 2.73 GGTCGCTCAGCAACAGTAGT 60.13 52.38 4.06 TCATAGCACTCACCCAGCATG 48 20 165 21

260 ssr-1-61 5 120 60.04 55.0 2.73 GGTCGCTCAGCAACAGTAGT 60.13 52.38 4.41 CATCATAGCACTCACCCAGCA 48 20 167 21

261 ssr-1-62 1 154 59.59 47.62 3.86 GGCGAATAAATTGCAGCAGGA 60.04 50.0 5.92 CTTTTTGTGCTGCAAAGCGC 48 21 201 20

262 ssr-1-62 2 155 59.59 47.62 4.85 AGGCGAATAAATTGCAGCAGG 60.04 50.0 5.92 CTTTTTGTGCTGCAAAGCGC 47 21 201 20

263 ssr-1-62 3 218 59.59 47.62 3.86 GGCGAATAAATTGCAGCAGGA 59.9 55.0 2.62 GCGGCACACATGATCCTTTC 48 21 265 20

264 ssr-1-62 4 219 59.59 47.62 4.85 AGGCGAATAAATTGCAGCAGG 59.9 55.0 2.62 GCGGCACACATGATCCTTTC 47 21 265 20

265 ssr-1-62 5 189 59.59 47.62 3.86 GGCGAATAAATTGCAGCAGGA 59.68 50.0 5.54 ACACAATAACTCACCGCGGA 48 21 236 20

266 ssr-1-63 1 103 59.96 55.0 4.57 GTTCAGATGACCCTGGTGCA 59.83 50.0 4.51 GCAAATCATAGTCCTCCTGGGT 35 20 137 22

267 ssr-1-63 2 104 59.96 55.0 5.01 TGTTCAGATGACCCTGGTGC 59.83 50.0 4.51 GCAAATCATAGTCCTCCTGGGT 34 20 137 22

268 ssr-1-63 3 135 60.18 55.0 3.06 ACTTCCCTCCCCACTGTCAT 59.83 50.0 4.51 GCAAATCATAGTCCTCCTGGGT 3 20 137 22

269 ssr-1-63 4 104 59.96 55.0 4.57 GTTCAGATGACCCTGGTGCA 59.56 50.0 4.45 AGCAAATCATAGTCCTCCTGGG 35 20 138 22

270 ssr-1-63 5 105 59.96 55.0 5.01 TGTTCAGATGACCCTGGTGC 59.56 50.0 4.45 AGCAAATCATAGTCCTCCTGGG 34 20 138 22

271 ssr-1-65 1 110 60.18 50.0 3.67 TCTCGCACACGTTTCTTGGT 59.96 50.0 3.73 ACGTTGCCTACCCAAACGAT 63 20 172 20

272 ssr-1-65 2 117 60.18 50.0 3.67 TCTCGCACACGTTTCTTGGT 60.04 55.0 3.69 ACTTGCTACGTTGCCTACCC 63 20 179 20

273 ssr-1-65 3 116 60.18 50.0 3.67 TCTCGCACACGTTTCTTGGT 60.04 55.0 4.51 CTTGCTACGTTGCCTACCCA 63 20 178 20

274 ssr-1-65 4 114 60.18 50.0 3.67 TCTCGCACACGTTTCTTGGT 59.6 50.0 3.28 TGCTACGTTGCCTACCCAAA 63 20 176 20

275 ssr-1-65 5 115 60.18 50.0 3.67 TCTCGCACACGTTTCTTGGT 59.6 50.0 4.12 TTGCTACGTTGCCTACCCAA 63 20 177 20

276 ssr-1-66 1 181 57.98 36.0 3.41 TCTTTGTGGGATATTTCTCTTCACT 58.74 47.62 4.26 TGGTCATCCTTACTGTGCTGA 16 25 196 21

277 ssr-1-66 2 181 57.98 36.0 3.41 TCTTTGTGGGATATTTCTCTTCACT 59.36 45.45 3.02 TGGTCATCCTTACTGTGCTGAA 16 25 196 22

278 ssr-1-66 3 181 58.54 34.62 3.16 TCTTTGTGGGATATTTCTCTTCACTT 58.74 47.62 4.26 TGGTCATCCTTACTGTGCTGA 16 26 196 21

279 ssr-1-66 4 182 58.54 34.62 3.41 TTCTTTGTGGGATATTTCTCTTCACT 58.74 47.62 4.26 TGGTCATCCTTACTGTGCTGA 15 26 196 21

280 ssr-1-66 5 135 57.53 36.0 3.16 TCCTCTTTATGAGAAAACCAAATGG 58.74 47.62 4.26 TGGTCATCCTTACTGTGCTGA 62 25 196 21

281 ssr-1-67 1 180 60.32 55.0 2.17 ACTCGGGTGCCAGTCAAATC 59.9 50.0 4.79 TCGTCCCATTTCCTAATCGTCC 26 20 205 22

282 ssr-1-67 2 178 60.32 55.0 2.17 ACTCGGGTGCCAGTCAAATC 59.83 50.0 4.37 GTCCCATTTCCTAATCGTCCCA 26 20 203 22

283 ssr-1-67 3 173 59.6 50.0 4.2 TGCCAGTCAAATCGTTCCCT 59.9 50.0 4.79 TCGTCCCATTTCCTAATCGTCC 33 20 205 22

284 ssr-1-67 4 171 59.6 50.0 4.2 TGCCAGTCAAATCGTTCCCT 59.83 50.0 4.37 GTCCCATTTCCTAATCGTCCCA 33 20 203 22

285 ssr-1-67 5 174 59.48 55.0 3.97 GTGCCAGTCAAATCGTTCCC 59.9 50.0 4.79 TCGTCCCATTTCCTAATCGTCC 32 20 205 22

286 ssr-1-68 1 120 57.84 50.0 3.99 AGGTTAGGCTAGGCTACGTT 59.6 50.0 4.75 AGCAAGTTGAGTGGATGGCT 77 20 196 20

287 ssr-1-68 2 121 57.84 50.0 3.99 AGGTTAGGCTAGGCTACGTT 59.48 55.0 4.4 CAGCAAGTTGAGTGGATGGC 77 20 197 20

288 ssr-1-68 3 119 59.7 54.55 2.69 GGTTAGGCTAGGCTACGTTAGG 59.6 50.0 4.75 AGCAAGTTGAGTGGATGGCT 78 22 196 20

289 ssr-1-68 4 124 59.7 54.55 3.51 GGTTAGGTTAGGCTAGGCTACG 59.6 50.0 4.75 AGCAAGTTGAGTGGATGGCT 73 22 196 20

290 ssr-1-68 5 120 59.7 54.55 2.69 GGTTAGGCTAGGCTACGTTAGG 59.48 55.0 4.4 CAGCAAGTTGAGTGGATGGC 78 22 197 20

291 ssr-1-70 1 147 59.96 55.0 4.94 TTCCCATTCAGACACCGTGG 59.79 47.62 4.34 ACATAGCAATGCTCTGGACGT 76 20 222 21

292 ssr-1-70 2 205 60.25 55.0 4.17 AAAGTCCCCGGTGAATGGTG 59.79 47.62 4.34 ACATAGCAATGCTCTGGACGT 18 20 222 21

293 ssr-1-70 3 196 59.67 55.0 3.53 GGTGAATGGTGGGGATCCAA 59.79 47.62 4.34 ACATAGCAATGCTCTGGACGT 27 20 222 21

294 ssr-1-70 4 183 60.4 60.0 2.94 GATCCAACCCGGGTCTCCTA 59.79 47.62 4.34 ACATAGCAATGCTCTGGACGT 40 20 222 21

295 ssr-1-70 5 182 60.4 60.0 3.02 ATCCAACCCGGGTCTCCTAG 59.79 47.62 4.34 ACATAGCAATGCTCTGGACGT 41 20 222 21

296 ssr-1-71 1 159 57.23 45.0 1.9 TCACAAGCGCCCTTACAATA 59.81 55.0 4.51 ACCTAACCTCACCTCACCCA 0 20 158 20

297 ssr-1-71 2 159 57.72 42.86 1.28 TCACAAGCGCCCTTACAATAT 59.81 55.0 4.51 ACCTAACCTCACCTCACCCA 0 21 158 20

298 ssr-1-71 3 154 57.23 45.0 1.9 TCACAAGCGCCCTTACAATA 59.08 50.0 2.83 ACCTCACCTCACCCAAAACA 0 20 153 20

299 ssr-1-71 4 161 57.23 45.0 1.9 TCACAAGCGCCCTTACAATA 60.0 57.14 4.61 CAACCTAACCTCACCTCACCC 0 20 160 21

300 ssr-1-71 5 162 57.23 45.0 1.9 TCACAAGCGCCCTTACAATA 60.0 57.14 4.02 CCAACCTAACCTCACCTCACC 0 20 161 21

301 ssr-1-72 1 174 60.11 60.0 4.0 CAGAGTGGCTACGTGACTGG 59.9 55.0 2.92 CGACCACTGACGCTTCGATA 27 20 200 20

302 ssr-1-72 2 172 60.11 60.0 4.02 GAGTGGCTACGTGACTGGAC 59.9 55.0 2.92 CGACCACTGACGCTTCGATA 29 20 200 20

303 ssr-1-72 3 181 60.11 60.0 4.0 CAGAGTGGCTACGTGACTGG 59.83 55.0 5.19 ATTAGACCGACCACTGACGC 27 20 207 20

304 ssr-1-72 4 179 60.11 60.0 4.02 GAGTGGCTACGTGACTGGAC 59.83 55.0 5.19 ATTAGACCGACCACTGACGC 29 20 207 20

305 ssr-1-72 5 173 60.25 55.0 3.86 AGAGTGGCTACGTGACTGGA 59.9 55.0 2.92 CGACCACTGACGCTTCGATA 28 20 200 20

306 ssr-1-73 1 100 58.86 47.62 3.58 GTTATCGTTGCTGTGGTGACA 57.07 45.0 3.55 AAGTGACGGAATTCCAGTGT 53 21 152 20

307 ssr-1-73 2 101 58.86 47.62 3.67 TGTTATCGTTGCTGTGGTGAC 57.07 45.0 3.55 AAGTGACGGAATTCCAGTGT 52 21 152 20

308 ssr-1-73 3 101 58.86 47.62 3.58 GTTATCGTTGCTGTGGTGACA 57.79 42.86 3.55 AAAGTGACGGAATTCCAGTGT 53 21 153 21

309 ssr-1-73 4 102 58.86 47.62 3.67 TGTTATCGTTGCTGTGGTGAC 57.79 42.86 3.55 AAAGTGACGGAATTCCAGTGT 52 21 153 21

310 ssr-1-73 5 101 60.48 45.45 3.58 TGTTATCGTTGCTGTGGTGACA 57.07 45.0 3.55 AAGTGACGGAATTCCAGTGT 52 22 152 20

311 ssr-1-74 1 104 57.1 45.0 4.0 TCGTTACAGCAGTTTCCTGT 57.67 30.77 3.07 TGCTAAAGTCATTGTTGTTATCATCA 52 20 155 26

312 ssr-1-74 2 104 57.81 42.86 3.16 TCGTTACAGCAGTTTCCTGTT 57.67 30.77 3.07 TGCTAAAGTCATTGTTGTTATCATCA 52 21 155 26

313 ssr-1-74 3 105 57.1 45.0 4.0 TCGTTACAGCAGTTTCCTGT 58.21 29.63 3.07 TTGCTAAAGTCATTGTTGTTATCATCA 52 20 156 27

314 ssr-1-74 4 105 57.6 42.86 4.0 ATCGTTACAGCAGTTTCCTGT 57.67 30.77 3.07 TGCTAAAGTCATTGTTGTTATCATCA 51 21 155 26

315 ssr-1-74 5 107 59.5 43.48 4.0 CAATCGTTACAGCAGTTTCCTGT 57.67 30.77 3.07 TGCTAAAGTCATTGTTGTTATCATCA 49 23 155 26

316 ssr-1-78 1 155 59.96 50.0 2.27 TGACATCTCAGCGGGCTTTT 59.66 47.62 4.18 TGTCTTGAGTGACATGCGAGT 31 20 185 21

317 ssr-1-78 2 157 59.96 50.0 2.27 TGACATCTCAGCGGGCTTTT 59.39 47.62 5.1 TCTGTCTTGAGTGACATGCGA 31 20 187 21

318 ssr-1-78 3 149 59.61 50.0 3.32 CTCAGCGGGCTTTTTGTTGT 59.66 47.62 4.18 TGTCTTGAGTGACATGCGAGT 37 20 185 21

319 ssr-1-78 4 128 59.61 50.0 4.79 TTTGTTTTGCCCATGGTCGG 59.66 47.62 4.18 TGTCTTGAGTGACATGCGAGT 58 20 185 21

320 ssr-1-78 5 156 60.39 55.0 3.51 CTGACATCTCAGCGGGCTTT 59.66 47.62 4.18 TGTCTTGAGTGACATGCGAGT 30 20 185 21

321 ssr-1-79 1 155 59.96 50.0 2.27 TGACATCTCAGCGGGCTTTT 59.66 47.62 4.18 TGTCTTGAGTGACATGCGAGT 11 20 165 21

322 ssr-1-79 2 157 59.96 50.0 2.27 TGACATCTCAGCGGGCTTTT 59.39 47.62 5.1 TCTGTCTTGAGTGACATGCGA 11 20 167 21

323 ssr-1-79 3 149 59.61 50.0 3.32 CTCAGCGGGCTTTTTGTTGT 59.66 47.62 4.18 TGTCTTGAGTGACATGCGAGT 17 20 165 21

324 ssr-1-79 4 128 59.61 50.0 4.79 TTTGTTTTGCCCATGGTCGG 59.66 47.62 4.18 TGTCTTGAGTGACATGCGAGT 38 20 165 21

325 ssr-1-79 5 156 60.39 55.0 3.51 CTGACATCTCAGCGGGCTTT 59.66 47.62 4.18 TGTCTTGAGTGACATGCGAGT 10 20 165 21

326 ssr-1-80 1 120 59.36 43.48 2.87 ACAAGGTCGTTTAGTGAGGAAGA 59.9 55.0 2.92 GCTGCAGAGGACGATGATGA 25 23 144 20

327 ssr-1-80 2 124 59.12 43.48 3.86 ACAAACAAGGTCGTTTAGTGAGG 59.9 55.0 2.92 GCTGCAGAGGACGATGATGA 21 23 144 20

328 ssr-1-80 3 126 59.36 43.48 2.87 ACAAGGTCGTTTAGTGAGGAAGA 60.4 55.0 4.2 TATGAGGCTGCAGAGGACGA 25 23 150 20

329 ssr-1-80 4 120 58.01 45.45 3.46 ACAAGGTCGTTTAGTGAGGAAG 59.9 55.0 2.92 GCTGCAGAGGACGATGATGA 25 22 144 20

330 ssr-1-80 5 117 59.9 41.67 2.83 AGGTCGTTTAGTGAGGAAGAAACA 59.9 55.0 2.92 GCTGCAGAGGACGATGATGA 28 24 144 20

331 ssr-1-81 1 131 59.7 43.48 4.57 CGTGTTTCTCAACCTTTTTGTGC 60.18 60.0 3.79 GGGTGACGAGGATGACGAAG 73 23 203 20

332 ssr-1-81 2 134 59.7 43.48 4.57 CGTGTTTCTCAACCTTTTTGTGC 59.75 55.0 4.35 AAAGGGTGACGAGGATGACG 73 23 206 20

333 ssr-1-81 3 176 59.42 43.48 3.41 TGCCCTCTTTGTCTTCATACTCA 60.18 60.0 3.79 GGGTGACGAGGATGACGAAG 28 23 203 20

334 ssr-1-81 4 179 59.42 43.48 3.41 TGCCCTCTTTGTCTTCATACTCA 59.75 55.0 4.35 AAAGGGTGACGAGGATGACG 28 23 206 20

335 ssr-1-81 5 132 59.7 43.48 4.57 CGTGTTTCTCAACCTTTTTGTGC 60.61 55.0 3.85 AGGGTGACGAGGATGACGAA 73 23 204 20

336 ssr-1-82 1 108 59.41 41.67 3.67 AGTTGAGAAGATTGAGGAGAACCA 60.04 55.0 4.44 CCTGCATCTGACACTCCGTT 64 24 171 20

337 ssr-1-82 2 110 59.41 41.67 3.67 AGTTGAGAAGATTGAGGAGAACCA 59.75 55.0 4.63 TTCCTGCATCTGACACTCCG 64 24 173 20

338 ssr-1-82 3 145 59.41 41.67 3.67 AGTTGAGAAGATTGAGGAGAACCA 60.25 50.0 5.28 TCATCGACAACTTTGCCGGT 64 24 208 20

339 ssr-1-82 4 107 58.1 43.48 3.67 GTTGAGAAGATTGAGGAGAACCA 60.04 55.0 4.44 CCTGCATCTGACACTCCGTT 65 23 171 20

340 ssr-1-82 5 113 58.02 39.13 3.71 AGCAAAGTTGAGAAGATTGAGGA 60.04 55.0 4.44 CCTGCATCTGACACTCCGTT 59 23 171 20

341 ssr-1-83 1 144 60.46 55.0 5.52 GACAAGCAATGCTTCAGCGG 59.89 55.0 3.69 CCCCACACAGAAAACGGGTA 9 20 152 20

342 ssr-1-83 2 145 60.46 55.0 5.18 GGACAAGCAATGCTTCAGCG 59.89 55.0 3.69 CCCCACACAGAAAACGGGTA 8 20 152 20

343 ssr-1-83 3 101 59.59 55.0 4.26 ACCACTAGACCCAGAGCAGA 60.46 55.0 4.44 TGACCCCACACAGAAAACGG 55 20 155 20

344 ssr-1-83 4 142 59.2 50.0 4.02 CAAGCAATGCTTCAGCGGTA 59.89 55.0 3.69 CCCCACACAGAAAACGGGTA 11 20 152 20

345 ssr-1-83 5 147 60.46 55.0 5.52 GACAAGCAATGCTTCAGCGG 60.46 55.0 4.44 TGACCCCACACAGAAAACGG 9 20 155 20

346 ssr-1-84 1 183 59.89 55.0 4.96 TACCCGTTTTCTGTGTGGGG 60.1 50.0 4.61 CCACACATTTCTTAAGCGTGGG 7 20 189 22

347 ssr-1-84 2 115 60.32 55.0 4.61 AAACGTCGTATGTGGGGTGG 60.1 50.0 4.61 CCACACATTTCTTAAGCGTGGG 75 20 189 22

348 ssr-1-84 3 117 59.61 50.0 4.95 ACAAACGTCGTATGTGGGGT 60.1 50.0 4.61 CCACACATTTCTTAAGCGTGGG 73 20 189 22

349 ssr-1-84 4 180 60.46 55.0 4.02 CCGTTTTCTGTGTGGGGTCA 60.1 50.0 4.61 CCACACATTTCTTAAGCGTGGG 10 20 189 22

350 ssr-1-84 5 184 59.89 55.0 4.96 TACCCGTTTTCTGTGTGGGG 59.45 45.45 4.94 ACCACACATTTCTTAAGCGTGG 7 20 190 22

351 ssr-1-85 1 172 59.75 55.0 3.01 AATGGACGAGGCAGGAAGAC 60.11 55.0 2.57 CGCGTTCAGACTCCCTTCAT 3 20 174 20

352 ssr-1-85 2 162 60.32 55.0 3.02 GCAGGAAGACACGAGCTTCA 60.11 55.0 2.57 CGCGTTCAGACTCCCTTCAT 13 20 174 20

353 ssr-1-85 3 200 59.75 55.0 3.01 AATGGACGAGGCAGGAAGAC 60.39 55.0 5.14 TGACTCAAATCCGCTTCCCG 3 20 202 20

354 ssr-1-85 4 190 60.32 55.0 3.02 GCAGGAAGACACGAGCTTCA 60.39 55.0 5.14 TGACTCAAATCCGCTTCCCG 13 20 202 20

355 ssr-1-85 5 174 59.75 55.0 3.01 AATGGACGAGGCAGGAAGAC 60.73 60.0 3.46 GTCGCGTTCAGACTCCCTTC 3 20 176 20

356 ssr-1-86 1 145 60.25 55.0 4.2 TGCCATCTCTTCCCTTCCCT 59.8 55.0 4.2 AGGGAAGGAAAGGGAAGGGA 26 20 170 20

357 ssr-1-86 2 150 60.25 55.0 4.2 TGCCATCTCTTCCCTTCCCT 59.8 55.0 4.2 AGGGAAGGGAAGGAAAGGGA 26 20 175 20

358 ssr-1-86 3 146 59.37 55.0 3.97 TTGCCATCTCTTCCCTTCCC 59.8 55.0 4.2 AGGGAAGGAAAGGGAAGGGA 25 20 170 20

359 ssr-1-86 4 151 59.37 55.0 3.97 TTGCCATCTCTTCCCTTCCC 59.8 55.0 4.2 AGGGAAGGGAAGGAAAGGGA 25 20 175 20

360 ssr-1-86 5 160 59.15 50.0 4.92 TCAACTCCCCTTTCTTGCCA 59.8 55.0 4.2 AGGGAAGGAAAGGGAAGGGA 11 20 170 20

361 ssr-1-87 1 145 60.25 55.0 4.2 TGCCATCTCTTCCCTTCCCT 59.8 55.0 4.2 AGGGAAGGAAAGGGAAGGGA 6 20 150 20

362 ssr-1-87 2 150 60.25 55.0 4.2 TGCCATCTCTTCCCTTCCCT 59.8 55.0 4.2 AGGGAAGGGAAGGAAAGGGA 6 20 155 20

363 ssr-1-87 3 149 59.8 55.0 4.2 TCCTTTCCCTTCCCTTCCCT 59.6 50.0 2.01 AGGAAGGGAAACGCGGTAAA 76 20 224 20

364 ssr-1-87 4 219 60.25 55.0 4.2 TGCCATCTCTTCCCTTCCCT 59.6 50.0 2.01 AGGAAGGGAAACGCGGTAAA 6 20 224 20

365 ssr-1-87 5 146 59.37 55.0 3.97 TTGCCATCTCTTCCCTTCCC 59.8 55.0 4.2 AGGGAAGGAAAGGGAAGGGA 5 20 150 20

366 ssr-1-88 1 171 60.04 55.0 3.46 TACCGCGTTTCCCTTCCTTC 57.08 33.33 2.01 GTGTAGTTTGTTCCTACATAGAGTAAA 44 20 214 27

367 ssr-1-88 2 163 59.8 55.0 4.2 TTCCCTTCCTTCCCTTCCCT 57.08 33.33 2.01 GTGTAGTTTGTTCCTACATAGAGTAAA 52 20 214 27

368 ssr-1-88 3 167 60.25 55.0 3.95 GCGTTTCCCTTCCTTCCCTT 57.08 33.33 2.01 GTGTAGTTTGTTCCTACATAGAGTAAA 48 20 214 27

369 ssr-1-88 4 159 60.32 60.0 4.57 CTTCCTTCCCTTCCCTGCAC 57.08 33.33 2.01 GTGTAGTTTGTTCCTACATAGAGTAAA 56 20 214 27

370 ssr-1-88 5 172 59.6 50.0 3.36 TTACCGCGTTTCCCTTCCTT 57.08 33.33 2.01 GTGTAGTTTGTTCCTACATAGAGTAAA 43 20 214 27

371 ssr-1-89 1 148 60.0 52.38 3.2 TGCCAACCACAGAATCCTCTC 57.41 50.0 4.34 AGGTTGGGATAGCTTAGGGT 19 21 166 20

372 ssr-1-89 2 178 60.0 52.38 3.2 TGCCAACCACAGAATCCTCTC 57.41 50.0 4.34 AGGTTGGGATAGCTTAGGGT 19 21 196 20

373 ssr-1-89 3 147 60.0 52.38 3.2 TGCCAACCACAGAATCCTCTC 59.36 54.55 3.18 GGTTGGGATAGCTTAGGGTAGG 19 21 165 22

374 ssr-1-89 4 177 60.0 52.38 3.2 TGCCAACCACAGAATCCTCTC 59.36 54.55 3.18 GGTTGGGATAGCTTAGGGTAGG 19 21 195 22

375 ssr-1-89 5 192 60.0 52.38 3.2 TGCCAACCACAGAATCCTCTC 59.36 54.55 4.12 GGCTAGGATAGGTTAGGTTGGG 19 21 210 22

376 ssr-1-90 1 108 58.91 41.67 2.87 ACCAGGTTAAGTATGGTCAGAAGA 59.6 47.62 4.92 GCATGTATTACAGCGTTGGCA 43 24 150 21

377 ssr-1-90 2 109 58.91 41.67 2.87 ACCAGGTTAAGTATGGTCAGAAGA 59.33 47.62 4.52 AGCATGTATTACAGCGTTGGC 43 24 151 21

378 ssr-1-90 3 108 57.58 43.48 2.85 ACCAGGTTAAGTATGGTCAGAAG 59.6 47.62 4.92 GCATGTATTACAGCGTTGGCA 43 23 150 21

379 ssr-1-90 4 108 59.45 40.0 2.52 ACCAGGTTAAGTATGGTCAGAAGAA 59.6 47.62 4.92 GCATGTATTACAGCGTTGGCA 43 25 150 21

380 ssr-1-90 5 107 57.32 43.48 2.87 CCAGGTTAAGTATGGTCAGAAGA 59.6 47.62 4.92 GCATGTATTACAGCGTTGGCA 44 23 150 21

381 ssr-1-91 1 133 59.96 55.0 2.85 ACCTTCTTGGCGGGAAAGAG 60.32 60.0 2.74 CCAACCGTCCAACCCTAGTG 72 20 204 20

382 ssr-1-91 2 132 59.96 55.0 2.85 ACCTTCTTGGCGGGAAAGAG 59.6 55.0 3.55 CAACCGTCCAACCCTAGTGT 72 20 203 20

383 ssr-1-91 3 135 59.96 55.0 2.85 ACCTTCTTGGCGGGAAAGAG 59.6 55.0 3.02 AACCAACCGTCCAACCCTAG 72 20 206 20

384 ssr-1-91 4 129 59.68 55.0 3.46 TCTTGGCGGGAAAGAGGAAG 60.32 60.0 2.74 CCAACCGTCCAACCCTAGTG 76 20 204 20

385 ssr-1-91 5 131 59.68 55.0 3.71 CTTCTTGGCGGGAAAGAGGA 60.32 60.0 2.74 CCAACCGTCCAACCCTAGTG 74 20 204 20

386 ssr-1-92 1 184 59.92 57.89 4.51 AGGTGATAGCCCAGTGGGT 60.39 55.0 3.02 ATTGGCGCCCTAGTGTGAAG 0 19 183 20

387 ssr-1-92 2 189 59.92 57.89 4.51 AGGTGATAGCCCAGTGGGT 60.46 55.0 2.74 TTGCTATTGGCGCCCTAGTG 0 19 188 20

388 ssr-1-92 3 186 59.92 57.89 4.51 AGGTGATAGCCCAGTGGGT 59.54 55.0 3.58 CTATTGGCGCCCTAGTGTGA 0 19 185 20

389 ssr-1-92 4 190 59.92 57.89 4.51 AGGTGATAGCCCAGTGGGT 59.53 50.0 2.57 ATTGCTATTGGCGCCCTAGT 0 19 189 20

390 ssr-1-92 5 178 58.73 55.0 3.32 TAGCCCAGTGGGTAGAACAC 60.39 55.0 3.02 ATTGGCGCCCTAGTGTGAAG 6 20 183 20

391 ssr-1-93 1 126 60.18 55.0 3.18 GTTTGGTGGACTCGGGTCAA 58.01 42.86 4.08 TCACACACTACCACTTTTGCA 36 20 161 21

392 ssr-1-93 2 128 60.32 60.0 4.46 GAGTTTGGTGGACTCGGGTC 58.01 42.86 4.08 TCACACACTACCACTTTTGCA 34 20 161 21

393 ssr-1-93 3 126 60.18 55.0 3.18 GTTTGGTGGACTCGGGTCAA 58.65 40.91 4.08 TCACACACTACCACTTTTGCAA 36 20 161 22

394 ssr-1-93 4 136 60.54 55.0 4.02 GCCTTTCCGAGTTTGGTGGA 58.01 42.86 4.08 TCACACACTACCACTTTTGCA 26 20 161 21

395 ssr-1-93 5 123 59.45 50.0 1.94 TGGTGGACTCGGGTCAAAAA 58.01 42.86 4.08 TCACACACTACCACTTTTGCA 39 20 161 21

396 ssr-1-94 1 126 60.18 55.0 3.18 GTTTGGTGGACTCGGGTCAA 58.01 42.86 4.08 TCACACACTACCACTTTTGCA 21 20 146 21

397 ssr-1-94 2 128 60.32 60.0 4.46 GAGTTTGGTGGACTCGGGTC 58.01 42.86 4.08 TCACACACTACCACTTTTGCA 19 20 146 21

398 ssr-1-94 3 126 60.18 55.0 3.18 GTTTGGTGGACTCGGGTCAA 58.65 40.91 4.08 TCACACACTACCACTTTTGCAA 21 20 146 22

399 ssr-1-94 4 136 60.54 55.0 4.02 GCCTTTCCGAGTTTGGTGGA 58.01 42.86 4.08 TCACACACTACCACTTTTGCA 11 20 146 21

400 ssr-1-94 5 123 59.45 50.0 1.94 TGGTGGACTCGGGTCAAAAA 58.01 42.86 4.08 TCACACACTACCACTTTTGCA 24 20 146 21

401 ssr-1-95 1 125 58.01 42.86 3.58 TGCAAAAGTGGTAGTGTGTGA 60.18 55.0 3.82 CCAGTTGGCAAAACCCACAC 47 21 171 20

402 ssr-1-95 2 121 58.01 42.86 3.58 TGCAAAAGTGGTAGTGTGTGA 59.75 50.0 3.82 TTGGCAAAACCCACACACAC 47 21 167 20

403 ssr-1-95 3 122 58.01 42.86 3.58 TGCAAAAGTGGTAGTGTGTGA 59.75 50.0 3.72 GTTGGCAAAACCCACACACA 47 21 168 20

404 ssr-1-95 4 171 58.01 42.86 3.58 TGCAAAAGTGGTAGTGTGTGA 60.25 55.0 3.77 TAACCTCGGTTGCAGGGTTG 47 21 217 20

405 ssr-1-95 5 159 58.01 42.86 3.58 TGCAAAAGTGGTAGTGTGTGA 59.6 55.0 3.08 CAGGGTTGCCACTTGAGGTA 47 21 205 20

406 ssr-1-96 1 207 60.18 55.0 2.69 GCTATTGGCGGGCTTCCTTA 59.97 55.0 3.02 CACTCTGCTCAGTGGCTTCA 12 20 218 20

407 ssr-1-96 2 202 60.18 55.0 2.69 GCTATTGGCGGGCTTCCTTA 60.04 55.0 3.86 TGCTCAGTGGCTTCATCAGG 12 20 213 20

408 ssr-1-96 3 196 60.18 55.0 2.69 GCTATTGGCGGGCTTCCTTA 59.96 55.0 3.53 GTGGCTTCATCAGGGTTGGA 12 20 207 20

409 ssr-1-96 4 197 60.18 55.0 2.69 GCTATTGGCGGGCTTCCTTA 59.96 55.0 3.77 AGTGGCTTCATCAGGGTTGG 12 20 208 20

410 ssr-1-96 5 185 60.25 55.0 3.77 TAACCTCGGTTGCAGGGTTG 59.97 55.0 3.02 CACTCTGCTCAGTGGCTTCA 34 20 218 20

411 ssr-1-97 1 170 60.47 55.0 3.77 AAGTCAGGGTTGCCAGTTGG 59.97 50.0 3.82 ACGCTGCACATCAAACACAC 2 20 171 20

412 ssr-1-97 2 153 60.47 55.0 3.77 AAGTCAGGGTTGCCAGTTGG 59.97 55.0 3.02 CACTCTGCTCAGTGGCTTCA 2 20 154 20

413 ssr-1-97 3 148 60.47 55.0 3.77 AAGTCAGGGTTGCCAGTTGG 60.04 55.0 3.86 TGCTCAGTGGCTTCATCAGG 2 20 149 20

414 ssr-1-97 4 142 60.47 55.0 3.77 AAGTCAGGGTTGCCAGTTGG 59.96 55.0 3.53 GTGGCTTCATCAGGGTTGGA 2 20 143 20

415 ssr-1-97 5 143 60.47 55.0 3.77 AAGTCAGGGTTGCCAGTTGG 59.96 55.0 3.77 AGTGGCTTCATCAGGGTTGG 2 20 144 20

416 ssr-1-98 1 172 59.75 55.0 3.66 TACCTCCATCGTGCACAGTG 59.47 50.0 2.83 CAGGTGGTGGTTTGCAACAA 4 20 175 20

417 ssr-1-98 2 170 60.32 55.0 3.18 CCTCCATCGTGCACAGTGAA 59.47 50.0 2.83 CAGGTGGTGGTTTGCAACAA 6 20 175 20

418 ssr-1-98 3 173 59.47 55.0 3.55 CTACCTCCATCGTGCACAGT 59.47 50.0 2.83 CAGGTGGTGGTTTGCAACAA 3 20 175 20

419 ssr-1-98 4 174 59.47 55.0 3.66 ACTACCTCCATCGTGCACAG 59.47 50.0 2.83 CAGGTGGTGGTTTGCAACAA 2 20 175 20

420 ssr-1-98 5 172 59.75 55.0 3.66 TACCTCCATCGTGCACAGTG 59.86 47.62 2.71 CAGGTGGTGGTTTGCAACAAT 4 20 175 21

421 ssr-1-99 1 103 60.23 57.89 4.85 GTTGCAAACCACCACCTGC 58.41 47.62 4.35 TCACTCACTTAGGTTCCACGA 40 19 142 21

422 ssr-1-99 2 103 60.23 57.89 4.85 GTTGCAAACCACCACCTGC 59.04 45.45 3.85 TCACTCACTTAGGTTCCACGAA 40 19 142 22

423 ssr-1-99 3 102 60.68 52.63 4.41 TTGCAAACCACCACCTGCA 58.41 47.62 4.35 TCACTCACTTAGGTTCCACGA 41 19 142 21

424 ssr-1-99 4 104 60.23 57.89 4.85 GTTGCAAACCACCACCTGC 58.84 45.45 4.35 ATCACTCACTTAGGTTCCACGA 40 19 143 22

425 ssr-1-99 5 102 61.95 55.0 4.57 TTGCAAACCACCACCTGCAC 58.41 47.62 4.35 TCACTCACTTAGGTTCCACGA 41 20 142 21

426 ssr-1-101 1 125 60.03 55.0 2.71 GGGTTTGATCCCCGGATTGT 60.13 52.38 4.75 GGGCTTCCTAAATAGCAGGCA 25 20 149 21

427 ssr-1-101 2 126 60.03 55.0 2.71 GGGTTTGATCCCCGGATTGT 60.13 52.38 4.85 TGGGCTTCCTAAATAGCAGGC 25 20 150 21

428 ssr-1-101 3 126 60.03 55.0 2.67 TGGGTTTGATCCCCGGATTG 60.13 52.38 4.75 GGGCTTCCTAAATAGCAGGCA 24 20 149 21

429 ssr-1-101 4 127 60.03 55.0 2.67 TGGGTTTGATCCCCGGATTG 60.13 52.38 4.85 TGGGCTTCCTAAATAGCAGGC 24 20 150 21

430 ssr-1-101 5 137 59.97 55.0 2.69 GCGAGAGGTCTTGGGTTTGA 60.13 52.38 4.75 GGGCTTCCTAAATAGCAGGCA 13 20 149 21

431 ssr-1-102 1 215 59.25 52.38 4.85 GATTCAGGCTAAACCACTGCC 57.53 33.33 1.94 TTCAAGCTTTCAGTCCTGATTTTT 1 21 215 24

432 ssr-1-102 2 214 58.16 50.0 4.85 ATTCAGGCTAAACCACTGCC 57.53 33.33 1.94 TTCAAGCTTTCAGTCCTGATTTTT 2 20 215 24

433 ssr-1-102 3 203 58.9 47.62 4.4 ACCACTGCCTTATCAAATGCC 57.53 33.33 1.94 TTCAAGCTTTCAGTCCTGATTTTT 13 21 215 24

434 ssr-1-102 4 203 60.29 45.45 4.75 ACCACTGCCTTATCAAATGCCT 57.53 33.33 1.94 TTCAAGCTTTCAGTCCTGATTTTT 13 22 215 24

435 ssr-1-102 5 202 58.62 47.62 4.75 CCACTGCCTTATCAAATGCCT 57.53 33.33 1.94 TTCAAGCTTTCAGTCCTGATTTTT 14 21 215 24

436 ssr-1-103 1 135 59.62 55.0 4.18 GCAAAGCTGATCCCAATCCG 57.53 33.33 1.94 TTTGAGCAAGTCTGGAATCTTTTT 33 20 167 24

437 ssr-1-103 2 136 59.62 55.0 4.18 GCAAAGCTGATCCCAATCCG 57.53 33.33 2.27 TTTTGAGCAAGTCTGGAATCTTTT 33 20 168 24

438 ssr-1-103 3 137 59.62 55.0 4.18 GCAAAGCTGATCCCAATCCG 57.53 33.33 2.52 TTTTTGAGCAAGTCTGGAATCTTT 33 20 169 24

439 ssr-1-103 4 130 59.31 55.0 3.24 GCTGATCCCAATCCGATCCT 57.53 33.33 1.94 TTTGAGCAAGTCTGGAATCTTTTT 38 20 167 24

440 ssr-1-103 5 131 59.31 55.0 3.24 GCTGATCCCAATCCGATCCT 57.53 33.33 2.27 TTTTGAGCAAGTCTGGAATCTTTT 38 20 168 24

441 ssr-1-104 1 100 58.16 42.86 3.71 TTGATGCAAAACTGACGGAGA 59.32 50.0 3.41 TCATGCCCGAGTTTCAGTGA 41 21 140 20

442 ssr-1-104 2 100 57.46 45.0 3.71 TGATGCAAAACTGACGGAGA 59.72 47.62 3.41 ATCATGCCCGAGTTTCAGTGA 42 20 141 21

443 ssr-1-104 3 100 58.79 40.91 2.87 TTGATGCAAAACTGACGGAGAA 59.32 50.0 3.41 TCATGCCCGAGTTTCAGTGA 41 22 140 20

444 ssr-1-104 4 100 58.16 42.86 3.71 TTGATGCAAAACTGACGGAGA 59.93 47.62 3.18 TCATGCCCGAGTTTCAGTGAA 41 21 140 21

445 ssr-1-104 5 100 59.75 43.48 2.85 TTGATGCAAAACTGACGGAGAAG 59.32 50.0 3.41 TCATGCCCGAGTTTCAGTGA 41 23 140 20

446 ssr-1-105 1 187 58.86 43.48 3.86 TGGATGTCAGAAACTGTTTGGAG 59.99 43.48 3.86 TGTCATGAACTTCGCTAACAGGA 6 23 192 23

447 ssr-1-105 2 187 60.14 41.67 3.71 TGGATGTCAGAAACTGTTTGGAGA 59.99 43.48 3.86 TGTCATGAACTTCGCTAACAGGA 6 24 192 23

448 ssr-1-105 3 187 57.84 40.91 3.53 TGGATGTCAGAAACTGTTTGGA 59.99 43.48 3.86 TGTCATGAACTTCGCTAACAGGA 6 22 192 23

449 ssr-1-105 4 195 58.86 43.48 3.86 TGGATGTCAGAAACTGTTTGGAG 59.88 43.48 4.7 GGCAAAATTGTCATGAACTTCGC 6 23 200 23

450 ssr-1-105 5 195 60.14 41.67 3.71 TGGATGTCAGAAACTGTTTGGAGA 59.88 43.48 4.7 GGCAAAATTGTCATGAACTTCGC 6 24 200 23

451 ssr-1-106 1 135 59.89 55.0 3.01 GGAAGTGTGTGTCCGGTCTT 59.96 55.0 4.92 GGTACACTGTGGTGATGGCA 59 20 193 20

452 ssr-1-106 2 137 59.89 55.0 4.79 AAGGAAGTGTGTGTCCGGTC 59.96 55.0 4.92 GGTACACTGTGGTGATGGCA 57 20 193 20

453 ssr-1-106 3 124 59.89 55.0 3.01 GGAAGTGTGTGTCCGGTCTT 59.96 55.0 2.9 GTGATGGCAGGGGTTGTGTA 59 20 182 20

454 ssr-1-106 4 126 59.89 55.0 4.79 AAGGAAGTGTGTGTCCGGTC 59.96 55.0 2.9 GTGATGGCAGGGGTTGTGTA 57 20 182 20

455 ssr-1-106 5 193 59.75 55.0 4.16 AATCCAGCACGACCTACACC 59.96 55.0 4.92 GGTACACTGTGGTGATGGCA 1 20 193 20

456 ssr-1-107 1 135 59.89 55.0 3.01 GGAAGTGTGTGTCCGGTCTT 59.96 55.0 4.92 GGTACACTGTGGTGATGGCA 42 20 176 20

457 ssr-1-107 2 137 59.89 55.0 4.79 AAGGAAGTGTGTGTCCGGTC 59.96 55.0 4.92 GGTACACTGTGGTGATGGCA 40 20 176 20

458 ssr-1-107 3 124 59.89 55.0 3.01 GGAAGTGTGTGTCCGGTCTT 59.96 55.0 2.9 GTGATGGCAGGGGTTGTGTA 42 20 165 20

459 ssr-1-107 4 126 59.89 55.0 4.79 AAGGAAGTGTGTGTCCGGTC 59.96 55.0 2.9 GTGATGGCAGGGGTTGTGTA 40 20 165 20

460 ssr-1-107 5 163 59.89 55.0 3.01 GGAAGTGTGTGTCCGGTCTT 59.75 55.0 3.66 TGAGACTGGTGATGGCATGG 42 20 204 20

461 ssr-1-108 1 189 59.18 50.0 3.41 TGAGACACACTGTGCACTGA 60.11 60.0 4.79 CCACATCTCAGGTGTCCGTC 24 20 212 20

462 ssr-1-108 2 190 59.79 47.62 3.41 TTGAGACACACTGTGCACTGA 60.11 60.0 4.79 CCACATCTCAGGTGTCCGTC 23 21 212 20

463 ssr-1-108 3 188 59.18 50.0 3.41 TGAGACACACTGTGCACTGA 59.4 55.0 4.35 CACATCTCAGGTGTCCGTCA 24 20 211 20

464 ssr-1-108 4 187 59.18 50.0 3.41 TGAGACACACTGTGCACTGA 59.4 55.0 3.67 ACATCTCAGGTGTCCGTCAC 24 20 210 20

465 ssr-1-108 5 189 59.59 47.62 2.9 TGAGACACACTGTGCACTGAT 60.11 60.0 4.79 CCACATCTCAGGTGTCCGTC 24 21 212 20

466 ssr-1-109 1 164 59.69 55.0 3.95 TCCTGAGGACGTTGACGAAC 59.97 50.0 3.02 GCATTGCAAGGTTGGCTTCA 0 20 163 20

467 ssr-1-109 2 166 59.69 55.0 3.95 TCCTGAGGACGTTGACGAAC 59.97 50.0 4.35 CAGCATTGCAAGGTTGGCTT 0 20 165 20

468 ssr-1-109 3 134 60.32 55.0 4.4 CAAGATGAGTGCCAGGCTGT 59.97 50.0 3.02 GCATTGCAAGGTTGGCTTCA 30 20 163 20

469 ssr-1-109 4 135 60.32 55.0 4.85 ACAAGATGAGTGCCAGGCTG 59.97 50.0 3.02 GCATTGCAAGGTTGGCTTCA 29 20 163 20

470 ssr-1-109 5 119 60.32 55.0 4.02 GCTGTATGCTTGGGTGTGGA 59.97 50.0 3.02 GCATTGCAAGGTTGGCTTCA 45 20 163 20

471 ssr-1-112 1 124 59.74 50.0 1.98 AGGCCCAGCATGCAAATCTA 60.13 52.38 4.26 CGAGGCATAGAAAGTGGAGCA 72 20 195 21

472 ssr-1-112 2 126 59.74 50.0 1.98 AGGCCCAGCATGCAAATCTA 59.86 52.38 3.86 AGCGAGGCATAGAAAGTGGAG 72 20 197 21

473 ssr-1-112 3 126 59.74 50.0 1.98 AGGCCCAGCATGCAAATCTA 58.8 50.0 4.02 AGCGAGGCATAGAAAGTGGA 72 20 197 20

474 ssr-1-112 4 171 59.68 50.0 4.4 AGACTACGCCCAAATGCACT 60.13 52.38 4.26 CGAGGCATAGAAAGTGGAGCA 25 20 195 21

475 ssr-1-112 5 173 59.68 50.0 4.4 AGACTACGCCCAAATGCACT 59.86 52.38 3.86 AGCGAGGCATAGAAAGTGGAG 25 20 197 21

476 ssr-1-113 1 155 58.81 45.45 3.5 ACTCAGCTCAAATCCCTAACCT 59.86 57.89 4.16 GTTGCAAACCACCCACCAC 1 22 155 19

477 ssr-1-113 2 133 58.81 45.45 3.5 ACTCAGCTCAAATCCCTAACCT 59.81 52.38 2.62 CACCGTGGTCATTCGTGAAAG 1 22 133 21

478 ssr-1-113 3 133 58.81 45.45 3.5 ACTCAGCTCAAATCCCTAACCT 58.78 50.0 2.69 CACCGTGGTCATTCGTGAAA 1 22 133 20

479 ssr-1-113 4 134 58.81 45.45 3.5 ACTCAGCTCAAATCCCTAACCT 61.23 55.0 3.18 GCACCGTGGTCATTCGTGAA 1 22 134 20

480 ssr-1-113 5 138 58.81 45.45 3.5 ACTCAGCTCAAATCCCTAACCT 60.3 57.89 2.67 ACCTGCACCGTGGTCATTC 1 22 138 19

481 ssr-1-114 1 146 59.88 55.0 3.57 TCCTCCTTCCCAGGTTACGT 59.82 55.0 1.75 TGCCGACCCTTGCTCTAATC 66 20 211 20

482 ssr-1-114 2 148 59.88 55.0 3.57 TCCTCCTTCCCAGGTTACGT 60.32 55.0 2.1 GTTGCCGACCCTTGCTCTAA 66 20 213 20

483 ssr-1-114 3 186 59.68 55.0 4.0 TGGCTCTGAGACGAAACCAG 59.82 55.0 1.75 TGCCGACCCTTGCTCTAATC 26 20 211 20

484 ssr-1-114 4 158 59.68 55.0 3.36 GTTCTGTTCCGCTCCTCCTT 59.82 55.0 1.75 TGCCGACCCTTGCTCTAATC 54 20 211 20

485 ssr-1-114 5 160 60.39 60.0 4.3 CAGTTCTGTTCCGCTCCTCC 59.82 55.0 1.75 TGCCGACCCTTGCTCTAATC 52 20 211 20

486 ssr-1-115 1 146 59.88 55.0 3.57 TCCTCCTTCCCAGGTTACGT 59.82 55.0 1.75 TGCCGACCCTTGCTCTAATC 4 20 149 20

487 ssr-1-115 2 148 59.88 55.0 3.57 TCCTCCTTCCCAGGTTACGT 60.32 55.0 2.1 GTTGCCGACCCTTGCTCTAA 4 20 151 20

488 ssr-1-115 3 145 59.88 55.0 3.57 TCCTCCTTCCCAGGTTACGT 59.53 55.0 2.4 GCCGACCCTTGCTCTAATCT 4 20 148 20

489 ssr-1-115 4 173 59.88 55.0 3.57 TCCTCCTTCCCAGGTTACGT 59.47 55.0 4.09 CACATGCCCCTTGAAAGAGC 4 20 176 20

490 ssr-1-115 5 174 59.88 55.0 3.57 TCCTCCTTCCCAGGTTACGT 59.47 55.0 2.85 GCACATGCCCCTTGAAAGAG 4 20 177 20

491 ssr-1-116 1 168 59.82 55.0 5.69 GATTAGAGCAAGGGTCGGCA 60.38 50.0 6.86 ACGAAAAGTAAAGCACGCGC 6 20 173 20

492 ssr-1-116 2 150 59.76 55.0 3.02 CAACCTGCGGCTCTTTCAAG 60.38 50.0 6.86 ACGAAAAGTAAAGCACGCGC 24 20 173 20

493 ssr-1-116 3 166 60.32 55.0 4.17 TTAGAGCAAGGGTCGGCAAC 60.38 50.0 6.86 ACGAAAAGTAAAGCACGCGC 8 20 173 20

494 ssr-1-116 4 141 59.82 55.0 5.69 GATTAGAGCAAGGGTCGGCA 60.53 60.0 3.1 CGCGCTCTCTCTCTCTCTCT 6 20 146 20

495 ssr-1-116 5 123 59.76 55.0 3.02 CAACCTGCGGCTCTTTCAAG 60.53 60.0 3.1 CGCGCTCTCTCTCTCTCTCT 24 20 146 20

496 ssr-1-117 1 150 59.76 55.0 3.02 CAACCTGCGGCTCTTTCAAG 60.38 50.0 6.86 ACGAAAAGTAAAGCACGCGC 9 20 158 20

497 ssr-1-117 2 140 59.47 55.0 4.57 CTCTTTCAAGGGGCATGTGC 60.38 50.0 6.86 ACGAAAAGTAAAGCACGCGC 19 20 158 20

498 ssr-1-117 3 141 59.47 55.0 3.21 GCTCTTTCAAGGGGCATGTG 60.38 50.0 6.86 ACGAAAAGTAAAGCACGCGC 18 20 158 20

499 ssr-1-117 4 149 60.89 55.0 3.61 AACCTGCGGCTCTTTCAAGG 60.38 50.0 6.86 ACGAAAAGTAAAGCACGCGC 10 20 158 20

500 ssr-1-117 5 118 58.96 55.0 1.98 CTCCTGCCACCGACATAGAT 60.38 50.0 6.86 ACGAAAAGTAAAGCACGCGC 41 20 158 20

501 ssr-1-119 1 125 59.37 52.38 3.28 AGTAGGCCAGGACTCTTTTGG 58.93 50.0 4.4 TGTGGACTCTCCTTTGGCAT 35 21 159 20

502 ssr-1-119 2 126 59.37 52.38 3.28 AGTAGGCCAGGACTCTTTTGG 58.93 50.0 4.92 ATGTGGACTCTCCTTTGGCA 35 21 160 20

503 ssr-1-119 3 150 59.37 52.38 3.28 AGTAGGCCAGGACTCTTTTGG 60.07 47.62 3.56 AGTTGTGTCATGCGTGATTGC 35 21 184 21

504 ssr-1-119 4 119 59.18 52.38 3.06 CCAGGACTCTTTTGGCATGTC 58.93 50.0 4.4 TGTGGACTCTCCTTTGGCAT 41 21 159 20

505 ssr-1-119 5 120 59.18 52.38 3.06 CCAGGACTCTTTTGGCATGTC 58.93 50.0 4.92 ATGTGGACTCTCCTTTGGCA 41 21 160 20

506 ssr-1-120 1 105 59.79 52.38 4.85 ACCTCGTCTTCTTCATCTGCC 60.52 55.0 6.86 TACGGTGAAGTGTATCGCGC 79 21 183 20

507 ssr-1-120 2 102 59.79 52.38 4.85 ACCTCGTCTTCTTCATCTGCC 60.81 55.0 4.4 GGTGAAGTGTATCGCGCCAT 79 21 180 20

508 ssr-1-120 3 101 59.79 52.38 4.85 ACCTCGTCTTCTTCATCTGCC 60.0 52.38 2.67 GTGAAGTGTATCGCGCCATTC 79 21 179 21

509 ssr-1-120 4 101 59.79 52.38 4.85 ACCTCGTCTTCTTCATCTGCC 58.99 50.0 3.16 GTGAAGTGTATCGCGCCATT 79 21 179 20

510 ssr-1-120 5 104 58.07 55.0 4.85 CCTCGTCTTCTTCATCTGCC 60.52 55.0 6.86 TACGGTGAAGTGTATCGCGC 80 20 183 20

511 ssr-1-121 1 120 60.07 52.38 4.16 GTCCTCCAATCCGAATGGTGT 59.82 55.0 4.79 TTAGGCATCAGCTTCCGTCC 67 21 186 20

512 ssr-1-121 2 121 60.07 52.38 4.17 TGTCCTCCAATCCGAATGGTG 59.82 55.0 4.79 TTAGGCATCAGCTTCCGTCC 66 21 186 20

513 ssr-1-121 3 110 59.87 52.38 3.01 CCGAATGGTGTCTTGCAATCC 59.82 55.0 4.79 TTAGGCATCAGCTTCCGTCC 77 21 186 20

514 ssr-1-121 4 115 59.87 52.38 4.01 CCAATCCGAATGGTGTCTTGC 59.82 55.0 4.79 TTAGGCATCAGCTTCCGTCC 72 21 186 20

515 ssr-1-121 5 119 59.79 52.38 3.67 TCCTCCAATCCGAATGGTGTC 59.82 55.0 4.79 TTAGGCATCAGCTTCCGTCC 68 21 186 20

516 ssr-1-122 1 138 60.55 55.0 3.32 TATGGGGACGGGTCTGTTGT 58.73 39.13 4.0 TGTACATTGTTTTGGTGTCTGGT 9 20 146 23

517 ssr-1-122 2 147 59.25 55.0 4.79 AACAGACGATATGGGGACGG 58.73 39.13 4.0 TGTACATTGTTTTGGTGTCTGGT 0 20 146 23

518 ssr-1-122 3 143 60.83 60.0 3.85 GACGATATGGGGACGGGTCT 58.73 39.13 4.0 TGTACATTGTTTTGGTGTCTGGT 4 20 146 23

519 ssr-1-122 4 144 60.83 60.0 4.46 AGACGATATGGGGACGGGTC 58.73 39.13 4.0 TGTACATTGTTTTGGTGTCTGGT 3 20 146 23

520 ssr-1-122 5 139 59.16 55.0 3.33 ATATGGGGACGGGTCTGTTG 58.73 39.13 4.0 TGTACATTGTTTTGGTGTCTGGT 8 20 146 23

521 ssr-1-123 1 137 58.99 40.91 3.01 TGCACCTCGTTGAAAAAGTTCT 59.34 41.67 3.58 TGGATTATCCTTCATGGGATGTCA 39 22 175 24

522 ssr-1-123 2 139 58.99 40.91 3.01 TGCACCTCGTTGAAAAAGTTCT 59.33 41.67 3.06 ACTGGATTATCCTTCATGGGATGT 39 22 177 24

523 ssr-1-123 3 142 58.99 40.91 3.01 TGCACCTCGTTGAAAAAGTTCT 59.27 41.67 4.37 TCAACTGGATTATCCTTCATGGGA 39 22 180 24

524 ssr-1-123 4 141 58.99 40.91 3.01 TGCACCTCGTTGAAAAAGTTCT 57.95 43.48 4.37 CAACTGGATTATCCTTCATGGGA 39 22 179 23

525 ssr-1-123 5 142 58.99 40.91 3.01 TGCACCTCGTTGAAAAAGTTCT 57.95 43.48 4.0 TCAACTGGATTATCCTTCATGGG 39 22 180 23

526 ssr-1-125 1 123 59.75 50.0 4.06 TTTCATGGCAGTGCAGCATG 60.04 55.0 4.41 CAACACACCACATCGTGCAG 36 20 158 20

527 ssr-1-125 2 125 59.75 50.0 4.41 CATTTCATGGCAGTGCAGCA 60.04 55.0 4.41 CAACACACCACATCGTGCAG 34 20 158 20

528 ssr-1-125 3 135 59.75 50.0 4.06 TTTCATGGCAGTGCAGCATG 59.9 55.0 3.82 CGACACACCACACAACACAC 36 20 170 20

529 ssr-1-125 4 137 59.75 50.0 4.06 TTTCATGGCAGTGCAGCATG 59.9 55.0 3.32 CACGACACACCACACAACAC 36 20 172 20

530 ssr-1-125 5 147 59.75 50.0 4.06 TTTCATGGCAGTGCAGCATG 59.9 55.0 3.82 CACAACACACCACGACACAC 36 20 182 20

531 ssr-1-126 1 103 60.18 55.0 2.73 AGCACCACAACCACCACTAC 60.04 60.0 3.18 GATGGTGGTGGTGGTGGTAG 73 20 175 20

532 ssr-1-126 2 101 59.82 55.0 3.25 CACCACAACCACCACTACCA 60.04 60.0 3.18 GATGGTGGTGGTGGTGGTAG 75 20 175 20

533 ssr-1-126 3 100 59.82 55.0 4.16 ACCACAACCACCACTACCAC 60.04 60.0 3.18 GATGGTGGTGGTGGTGGTAG 76 20 175 20

534 ssr-1-126 4 109 59.89 55.0 4.0 CTAGCACCACAACCACCACT 60.25 55.0 4.17 ATGTGATGGTGGTGGTGGTG 71 20 179 20

535 ssr-1-126 5 107 60.18 55.0 2.73 AGCACCACAACCACCACTAC 60.25 55.0 4.17 ATGTGATGGTGGTGGTGGTG 73 20 179 20

536 ssr-1-127 1 109 60.11 55.0 4.17 TACCACCACTACCACCACCA 58.02 55.0 2.59 CCTCAGTAGGCGATGGACTA 32 20 140 20

537 ssr-1-127 2 128 59.89 55.0 4.0 CTAGCACCACAACCACCACT 58.02 55.0 2.59 CCTCAGTAGGCGATGGACTA 13 20 140 20

538 ssr-1-127 3 126 60.18 55.0 2.73 AGCACCACAACCACCACTAC 58.02 55.0 2.59 CCTCAGTAGGCGATGGACTA 15 20 140 20

539 ssr-1-127 4 129 60.18 55.0 4.16 ACTAGCACCACAACCACCAC 58.02 55.0 2.59 CCTCAGTAGGCGATGGACTA 12 20 140 20

540 ssr-1-127 5 121 59.82 55.0 4.17 CACAACCACCACTACCACCA 58.02 55.0 2.59 CCTCAGTAGGCGATGGACTA 20 20 140 20

541 ssr-1-128 1 141 59.29 47.62 4.02 TCTTGCTTGGGTAATGGTGGA 59.61 41.67 4.52 TGATTAGATGACAACGACTTTGGC 10 21 150 24

542 ssr-1-128 2 142 58.91 52.38 4.61 CTCTTGCTTGGGTAATGGTGG 59.61 41.67 4.52 TGATTAGATGACAACGACTTTGGC 9 21 150 24

543 ssr-1-128 3 141 59.89 45.45 3.53 TCTTGCTTGGGTAATGGTGGAA 59.61 41.67 4.52 TGATTAGATGACAACGACTTTGGC 10 22 150 24

544 ssr-1-128 4 140 57.79 50.0 4.02 CTTGCTTGGGTAATGGTGGA 59.61 41.67 4.52 TGATTAGATGACAACGACTTTGGC 11 20 150 24

545 ssr-1-128 5 141 57.79 50.0 4.61 TCTTGCTTGGGTAATGGTGG 59.61 41.67 4.52 TGATTAGATGACAACGACTTTGGC 10 20 150 24

546 ssr-1-132 1 143 59.04 44.0 3.1 AGAAAGAGAGAGAGAGAGAGAGAGA 58.85 50.0 3.18 TGGGGAAATGGAGGTGTCAA 11 25 153 20

547 ssr-1-132 2 144 59.04 44.0 3.1 AGAAAGAGAGAGAGAGAGAGAGAGA 58.85 50.0 3.58 TTGGGGAAATGGAGGTGTCA 11 25 154 20

548 ssr-1-132 3 145 59.04 44.0 3.1 AGAGAAAGAGAGAGAGAGAGAGAGA 58.85 50.0 3.18 TGGGGAAATGGAGGTGTCAA 9 25 153 20

549 ssr-1-132 4 146 59.04 44.0 3.1 AGAGAAAGAGAGAGAGAGAGAGAGA 58.85 50.0 3.58 TTGGGGAAATGGAGGTGTCA 9 25 154 20

550 ssr-1-132 5 147 59.04 44.0 3.1 AGAGAGAAAGAGAGAGAGAGAGAGA 58.85 50.0 3.18 TGGGGAAATGGAGGTGTCAA 7 25 153 20

551 ssr-1-133 1 112 60.32 55.0 3.51 GACACAAACGCCAAGCTGAC 59.87 43.48 3.67 ACGAGTGTTTTAGGAGACAACCA 70 20 181 23

552 ssr-1-133 2 111 60.32 55.0 3.51 GACACAAACGCCAAGCTGAC 59.51 47.83 4.16 CGAGTGTTTTAGGAGACAACCAC 70 20 180 23

553 ssr-1-133 3 145 60.32 55.0 3.51 GACACAAACGCCAAGCTGAC 57.5 47.62 2.41 CAGTGGTACCTTGGGTTACAA 70 20 214 21

554 ssr-1-133 4 117 59.26 50.0 3.61 AAAGGGACACAAACGCCAAG 59.87 43.48 3.67 ACGAGTGTTTTAGGAGACAACCA 65 20 181 23

555 ssr-1-133 5 111 60.32 55.0 3.51 GACACAAACGCCAAGCTGAC 58.28 45.45 3.67 CGAGTGTTTTAGGAGACAACCA 70 20 180 22

556 ssr-1-138 1 197 59.45 34.62 3.41 TGTCTTTTCATCAGTTTCCATTGTCT 57.48 33.33 2.52 AGAAAGACAGACAAAAAGCAAGAA 12 26 208 24

557 ssr-1-138 2 197 57.29 33.33 2.71 TGTCTTTTCATCAGTTTCCATTGT 57.48 33.33 2.52 AGAAAGACAGACAAAAAGCAAGAA 12 24 208 24

558 ssr-1-138 3 197 58.26 36.0 3.18 TGTCTTTTCATCAGTTTCCATTGTC 57.48 33.33 2.52 AGAAAGACAGACAAAAAGCAAGAA 12 25 208 24

559 ssr-1-138 4 197 59.45 34.62 3.41 TGTCTTTTCATCAGTTTCCATTGTCT 58.08 32.0 2.52 AGAAAGACAGACAAAAAGCAAGAAA 12 26 208 25

560 ssr-1-138 5 192 57.04 33.33 3.02 TTTCATCAGTTTCCATTGTCTTGA 57.48 33.33 2.52 AGAAAGACAGACAAAAAGCAAGAA 17 24 208 24

561 ssr-1-139 1 129 59.09 55.0 3.55 GGAGCTCTTGATCCCACAGT 60.11 55.0 4.35 CCTTGCCTCTTATGCCGTGA 52 20 180 20

562 ssr-1-139 2 122 59.09 55.0 3.55 GGAGCTCTTGATCCCACAGT 59.82 55.0 5.01 TCTTATGCCGTGAGAGCCAC 52 20 173 20

563 ssr-1-139 3 126 60.0 52.38 3.58 GCTCTTGATCCCACAGTGTGA 60.11 55.0 4.35 CCTTGCCTCTTATGCCGTGA 55 21 180 20

564 ssr-1-139 4 127 60.0 52.38 3.82 AGCTCTTGATCCCACAGTGTG 60.11 55.0 4.35 CCTTGCCTCTTATGCCGTGA 54 21 180 20

565 ssr-1-139 5 124 59.09 55.0 3.55 GGAGCTCTTGATCCCACAGT 60.25 60.0 4.7 CCTCTTATGCCGTGAGAGCC 52 20 175 20

566 ssr-1-140 1 215 60.04 60.0 3.69 GACTGGTCACGGGAAAGGAG 59.89 61.11 4.16 TGACCGCTTGGGAACACC 23 20 237 18

567 ssr-1-140 2 217 60.04 60.0 3.69 GACTGGTCACGGGAAAGGAG 59.89 61.11 3.18 GGTGACCGCTTGGGAACA 23 20 239 18

568 ssr-1-140 3 216 60.04 60.0 3.69 GACTGGTCACGGGAAAGGAG 61.26 63.16 4.16 GTGACCGCTTGGGAACACC 23 20 238 19

569 ssr-1-140 4 217 60.04 60.0 3.69 GACTGGTCACGGGAAAGGAG 61.26 63.16 3.32 GGTGACCGCTTGGGAACAC 23 20 239 19

570 ssr-1-140 5 204 60.32 60.0 3.51 GGAAAGGAGGCAAGGGTGAG 59.89 61.11 4.16 TGACCGCTTGGGAACACC 34 20 237 18

571 ssr-1-141 1 131 57.34 25.93 1.82 TCCTTTTTGTAGTTTTGTAGCAATTTT 57.18 32.0 4.17 ACTACAAAAAGAGAAAATAGCCACA 10 27 140 25

572 ssr-1-141 2 132 57.34 25.93 1.82 TTCCTTTTTGTAGTTTTGTAGCAATTT 57.18 32.0 4.17 ACTACAAAAAGAGAAAATAGCCACA 9 27 140 25

573 ssr-1-141 3 133 57.34 25.93 2.32 TTTCCTTTTTGTAGTTTTGTAGCAATT 57.18 32.0 4.17 ACTACAAAAAGAGAAAATAGCCACA 8 27 140 25

574 ssr-1-141 4 134 57.34 25.93 3.56 TTTTCCTTTTTGTAGTTTTGTAGCAAT 57.18 32.0 4.17 ACTACAAAAAGAGAAAATAGCCACA 7 27 140 25

575 ssr-1-141 5 135 57.34 25.93 3.91 ATTTTCCTTTTTGTAGTTTTGTAGCAA 57.18 32.0 4.17 ACTACAAAAAGAGAAAATAGCCACA 6 27 140 25

576 ssr-1-143 1 172 59.82 55.0 5.73 TGTGTTGCTCATATCCCGGG 59.49 50.0 3.46 ACGACTCGTTGGCAATTTCG 39 20 210 20

577 ssr-1-143 2 169 59.82 55.0 5.73 TGTGTTGCTCATATCCCGGG 59.48 50.0 5.03 ACTCGTTGGCAATTTCGCTC 39 20 207 20

578 ssr-1-143 3 170 59.82 55.0 5.73 TGTGTTGCTCATATCCCGGG 59.48 50.0 4.93 GACTCGTTGGCAATTTCGCT 39 20 208 20

579 ssr-1-143 4 208 59.74 55.0 3.91 ACCCCCTTCAATCTGGATGC 59.49 50.0 3.46 ACGACTCGTTGGCAATTTCG 3 20 210 20

580 ssr-1-143 5 205 59.74 55.0 3.91 ACCCCCTTCAATCTGGATGC 59.48 50.0 5.03 ACTCGTTGGCAATTTCGCTC 3 20 207 20

581 ssr-1-144 1 161 61.03 60.0 4.41 CAGACCCATCTGTGCTGCTG 60.11 65.0 3.69 GAGGGAGGAGGAGGAAGAGG 2 20 162 20

582 ssr-1-144 2 162 61.03 60.0 4.41 CAGACCCATCTGTGCTGCTG 60.11 65.0 2.85 GGAGGGAGGAGGAGGAAGAG 2 20 163 20

583 ssr-1-144 3 201 61.03 60.0 4.41 CAGACCCATCTGTGCTGCTG 60.11 65.0 4.3 GAAGGAGGAGGAGGAGGAGG 2 20 202 20

584 ssr-1-144 4 202 61.03 60.0 4.41 CAGACCCATCTGTGCTGCTG 60.11 65.0 3.69 GGAAGGAGGAGGAGGAGGAG 2 20 203 20

585 ssr-1-144 5 170 61.03 60.0 4.41 CAGACCCATCTGTGCTGCTG 60.25 60.0 4.3 AAAAGAGGGGAGGGAGGAGG 2 20 171 20

586 ssr-1-145 1 100 59.71 57.14 4.3 TCTTCCTCTTCCTCCTCCTCC 59.82 60.0 3.79 CACCTCCATCCCAGACTTCG 60 21 159 20

587 ssr-1-145 2 100 59.66 60.0 4.3 TTCCTCTTCCTCCTCCTCCC 58.88 52.38 3.01 ATCACCTCCATCCCAGACTTC 62 20 161 21

588 ssr-1-145 3 101 60.29 54.55 4.3 TTCTTCCTCTTCCTCCTCCTCC 59.82 60.0 3.79 CACCTCCATCCCAGACTTCG 59 22 159 20

589 ssr-1-145 4 100 59.66 60.0 4.3 TTCCTCTTCCTCCTCCTCCC 57.72 50.0 3.01 ATCACCTCCATCCCAGACTT 62 20 161 20

590 ssr-1-145 5 101 59.71 57.14 4.3 TCTTCCTCTTCCTCCTCCTCC 58.43 55.0 3.01 TCACCTCCATCCCAGACTTC 60 21 160 20

591 ssr-1-146 1 104 59.75 55.0 2.74 TGTAGGTGCTGCTGCTGTAG 59.96 55.0 3.33 GCACATCAGACTCCCCAACA 73 20 176 20

592 ssr-1-146 2 105 59.75 55.0 2.74 CTGTAGGTGCTGCTGCTGTA 59.96 55.0 3.33 GCACATCAGACTCCCCAACA 72 20 176 20

593 ssr-1-146 3 101 59.75 55.0 2.74 TGTAGGTGCTGCTGCTGTAG 59.67 55.0 4.41 CATCAGACTCCCCAACAGCA 73 20 173 20

594 ssr-1-146 4 102 59.75 55.0 2.74 CTGTAGGTGCTGCTGCTGTA 59.67 55.0 4.41 CATCAGACTCCCCAACAGCA 72 20 173 20

595 ssr-1-146 5 102 59.75 55.0 2.74 TGTAGGTGCTGCTGCTGTAG 59.67 55.0 4.4 ACATCAGACTCCCCAACAGC 73 20 174 20

596 ssr-1-147 1 125 59.97 55.0 3.62 TCACTTGCCAGTCGAGGTTC 59.37 55.0 4.24 TAGTGAGAGAGAGGGCTGCT 75 20 199 20

597 ssr-1-147 2 124 59.97 55.0 3.01 CACTTGCCAGTCGAGGTTCT 59.37 55.0 4.24 TAGTGAGAGAGAGGGCTGCT 76 20 199 20

598 ssr-1-147 3 126 59.97 55.0 3.5 CTCACTTGCCAGTCGAGGTT 59.37 55.0 4.24 TAGTGAGAGAGAGGGCTGCT 74 20 199 20

599 ssr-1-147 4 122 59.97 55.0 3.62 TCACTTGCCAGTCGAGGTTC 60.68 60.0 3.35 TGAGAGAGAGGGCTGCTGAG 75 20 196 20

600 ssr-1-147 5 121 59.97 55.0 3.01 CACTTGCCAGTCGAGGTTCT 60.68 60.0 3.35 TGAGAGAGAGGGCTGCTGAG 76 20 196 20

601 ssr-1-148 1 129 59.24 50.0 5.19 CAGATAGGGTTGGAAAAAGCCC 58.56 50.0 2.52 TCCAACCCTGTAGGCAAAGA 51 22 179 20

602 ssr-1-148 2 135 59.24 50.0 5.19 CAGATAGGGTTGGAAAAAGCCC 59.38 52.38 3.93 GGTTTTTCCAACCCTGTAGGC 51 22 185 21

603 ssr-1-148 3 129 59.24 50.0 5.19 CAGATAGGGTTGGAAAAAGCCC 59.22 47.62 2.52 TCCAACCCTGTAGGCAAAGAA 51 22 179 21

604 ssr-1-148 4 130 59.24 50.0 5.19 CAGATAGGGTTGGAAAAAGCCC 59.22 47.62 2.52 TTCCAACCCTGTAGGCAAAGA 51 22 180 21

605 ssr-1-148 5 128 57.9 47.62 5.19 AGATAGGGTTGGAAAAAGCCC 58.56 50.0 2.52 TCCAACCCTGTAGGCAAAGA 52 21 179 20

606 ssr-1-149 1 136 58.56 50.0 3.53 TCTTTGCCTACAGGGTTGGA 59.18 43.48 4.0 ACAGATGACACCATTCAAACCTG 73 20 208 23

607 ssr-1-149 2 131 59.38 52.38 3.27 GCCTACAGGGTTGGAAAAACC 59.18 43.48 4.0 ACAGATGACACCATTCAAACCTG 78 21 208 23

608 ssr-1-149 3 135 58.56 50.0 3.53 TCTTTGCCTACAGGGTTGGA 58.93 43.48 3.86 CAGATGACACCATTCAAACCTGA 73 20 207 23

609 ssr-1-149 4 137 58.56 50.0 3.53 TCTTTGCCTACAGGGTTGGA 58.93 43.48 3.5 GACAGATGACACCATTCAAACCT 73 20 209 23

610 ssr-1-149 5 138 58.56 50.0 3.53 TCTTTGCCTACAGGGTTGGA 58.93 43.48 3.27 AGACAGATGACACCATTCAAACC 73 20 210 23

611 ssr-1-150 1 186 60.03 60.0 3.02 CCCCTTGTTCCTTGGCTAGG 57.86 45.0 2.69 TGTGGTGCTTTCCAGTCAAA 49 20 234 20

612 ssr-1-150 2 184 59.67 55.0 4.58 CCTTGTTCCTTGGCTAGGCT 57.86 45.0 2.69 TGTGGTGCTTTCCAGTCAAA 51 20 234 20

613 ssr-1-150 3 228 60.39 55.0 2.57 CCATCACAGGTGCCGATCAA 57.86 45.0 2.69 TGTGGTGCTTTCCAGTCAAA 7 20 234 20

614 ssr-1-150 4 190 60.47 55.0 4.52 TTGTCCCCTTGTTCCTTGGC 57.86 45.0 2.69 TGTGGTGCTTTCCAGTCAAA 45 20 234 20

615 ssr-1-150 5 185 60.03 60.0 3.02 CCCCTTGTTCCTTGGCTAGG 59.38 45.45 2.57 GTGGTGCTTTCCAGTCAAATCA 49 20 233 22

616 ssr-1-151 1 142 58.83 57.14 3.02 GCTAGGTTAGGCTAGGCTAGG 57.86 45.0 2.69 TGTGGTGCTTTCCAGTCAAA 22 21 163 20

617 ssr-1-151 2 143 58.83 57.14 3.02 GCTAGGTTAGGCTAGGCTAGG 57.86 45.0 3.18 TTGTGGTGCTTTCCAGTCAA 22 21 164 20

618 ssr-1-151 3 143 58.83 57.14 3.42 GGCTAGGTTAGGCTAGGCTAG 57.86 45.0 2.69 TGTGGTGCTTTCCAGTCAAA 21 21 163 20

619 ssr-1-151 4 144 58.83 57.14 3.42 GGCTAGGTTAGGCTAGGCTAG 57.86 45.0 3.18 TTGTGGTGCTTTCCAGTCAA 21 21 164 20

620 ssr-1-151 5 152 58.83 57.14 2.69 GCTAGGCTAGGCTAGGTTAGG 57.86 45.0 2.69 TGTGGTGCTTTCCAGTCAAA 12 21 163 20

621 ssr-1-152 1 100 58.72 41.67 3.86 TCCTCTGAAAATATCACTCCCAGA 59.05 40.91 3.02 AGCAGCAAAAAGTGTAGCTTCA 59 24 158 22

622 ssr-1-152 2 100 57.38 43.48 4.45 TCCTCTGAAAATATCACTCCCAG 59.05 40.91 3.02 AGCAGCAAAAAGTGTAGCTTCA 59 23 158 22

623 ssr-1-152 3 100 58.72 41.67 3.86 TCCTCTGAAAATATCACTCCCAGA 59.62 39.13 2.69 AGCAGCAAAAAGTGTAGCTTCAA 59 24 158 23

624 ssr-1-152 4 101 58.72 41.67 3.86 TCCTCTGAAAATATCACTCCCAGA 59.62 39.13 3.02 AAGCAGCAAAAAGTGTAGCTTCA 59 24 159 23

625 ssr-1-152 5 100 59.09 40.0 2.9 TCCTCTGAAAATATCACTCCCAGAT 59.05 40.91 3.02 AGCAGCAAAAAGTGTAGCTTCA 59 25 158 22

626 ssr-1-153 1 146 59.31 50.0 2.9 ACGACTTCACTTGCCCATCT 59.32 60.0 4.02 GGTCTGGGGATATCGTGTCC 67 20 212 20

627 ssr-1-153 2 157 59.25 50.0 3.02 TGTGGTGCAGTACGACTTCA 59.32 60.0 4.02 GGTCTGGGGATATCGTGTCC 56 20 212 20

628 ssr-1-153 3 151 59.21 55.0 4.52 GCAGTACGACTTCACTTGCC 59.32 60.0 4.02 GGTCTGGGGATATCGTGTCC 62 20 212 20

629 ssr-1-153 4 155 58.96 50.0 3.41 TGGTGCAGTACGACTTCACT 59.32 60.0 4.02 GGTCTGGGGATATCGTGTCC 58 20 212 20

630 ssr-1-153 5 149 58.95 50.0 5.36 AGTACGACTTCACTTGCCCA 59.32 60.0 4.02 GGTCTGGGGATATCGTGTCC 64 20 212 20

631 ssr-1-155 1 151 60.25 50.0 4.41 ATCCTCTGCTTTTGGCTGCA 60.04 55.0 2.83 GGCCTACGGGTGATCAACAA 31 20 181 20

632 ssr-1-155 2 152 59.54 55.0 5.25 GATCCTCTGCTTTTGGCTGC 60.04 55.0 2.83 GGCCTACGGGTGATCAACAA 30 20 181 20

633 ssr-1-155 3 153 60.25 50.0 4.41 ATCCTCTGCTTTTGGCTGCA 59.75 55.0 3.18 AAGGCCTACGGGTGATCAAC 31 20 183 20

634 ssr-1-155 4 162 59.46 55.0 4.26 ACTTCTGCCAGATCCTCTGC 60.04 55.0 2.83 GGCCTACGGGTGATCAACAA 20 20 181 20

635 ssr-1-155 5 155 60.25 50.0 4.41 ATCCTCTGCTTTTGGCTGCA 60.33 55.0 2.92 AGAAGGCCTACGGGTGATCA 31 20 185 20

636 ssr-1-156 1 184 60.04 50.0 5.18 AATGGTCTTTTGCTGCTGCG 59.76 55.0 5.36 GGTACACAACAAGCATGCCC 13 20 196 20

637 ssr-1-156 2 120 59.88 55.0 3.36 TCCTTCCTTCCGTCCTTCCT 59.76 55.0 5.36 GGTACACAACAAGCATGCCC 77 20 196 20

638 ssr-1-156 3 181 60.04 50.0 5.18 AATGGTCTTTTGCTGCTGCG 60.47 50.0 3.86 ACACAACAAGCATGCCCTGA 13 20 193 20

639 ssr-1-156 4 180 59.69 50.0 3.01 GTCTTTTGCTGCTGCGTCTT 59.76 55.0 5.36 GGTACACAACAAGCATGCCC 17 20 196 20

640 ssr-1-156 5 117 59.88 55.0 3.36 TCCTTCCTTCCGTCCTTCCT 60.47 50.0 3.86 ACACAACAAGCATGCCCTGA 77 20 193 20

641 ssr-1-157 1 101 58.85 45.45 3.72 GTGCTCCAACATTCCTAACACA 59.12 43.48 4.04 TGTTAGCGATCATGTAGACGAGA 38 22 138 23

642 ssr-1-157 2 102 58.85 45.45 3.32 TGTGCTCCAACATTCCTAACAC 59.12 43.48 4.04 TGTTAGCGATCATGTAGACGAGA 37 22 138 23

643 ssr-1-157 3 112 58.82 45.45 3.33 TCCCTTAGTATGTGCTCCAACA 59.12 43.48 4.04 TGTTAGCGATCATGTAGACGAGA 27 22 138 23

644 ssr-1-157 4 102 60.43 43.48 3.72 TGTGCTCCAACATTCCTAACACA 59.12 43.48 4.04 TGTTAGCGATCATGTAGACGAGA 37 23 138 23

645 ssr-1-157 5 101 58.85 45.45 3.72 GTGCTCCAACATTCCTAACACA 57.79 45.45 4.18 TGTTAGCGATCATGTAGACGAG 38 22 138 22

646 ssr-1-158 1 110 57.18 32.0 2.83 ATGTTAGAAGTGTTGGAGAATTTGT 59.75 50.0 3.85 AGCGTTCACATAAGCGAGGT 25 25 134 20

647 ssr-1-158 2 127 59.11 43.48 4.0 AGGACGTTATATAGCGAACAGGT 59.29 41.67 4.35 AGCTTGGAGAAACTATAGACACGA 73 23 199 24

648 ssr-1-158 3 127 60.02 45.83 3.85 AGGACGTTATATAGCGAACAGGTC 59.29 41.67 4.35 AGCTTGGAGAAACTATAGACACGA 73 24 199 24

649 ssr-1-158 4 128 60.02 45.83 4.0 GAGGACGTTATATAGCGAACAGGT 59.29 41.67 4.35 AGCTTGGAGAAACTATAGACACGA 72 24 199 24

650 ssr-1-158 5 126 59.11 43.48 4.0 AGGACGTTATATAGCGAACAGGT 58.0 43.48 4.35 GCTTGGAGAAACTATAGACACGA 73 23 198 23

651 ssr-1-159 1 134 60.32 60.0 3.86 CAGGTGATGGTGGTGTGAGG 60.11 55.0 4.0 CTTTACCGATCACGCCACCT 60 20 193 20

652 ssr-1-159 2 135 59.6 55.0 3.51 ACAGGTGATGGTGGTGTGAG 60.11 55.0 4.0 CTTTACCGATCACGCCACCT 59 20 193 20

653 ssr-1-159 3 131 59.6 55.0 3.97 GTGATGGTGGTGTGAGGGAA 60.11 55.0 4.0 CTTTACCGATCACGCCACCT 63 20 193 20

654 ssr-1-159 4 125 60.32 60.0 3.86 CAGGTGATGGTGGTGTGAGG 59.74 55.0 2.57 TCACGCCACCTAGGTCCTAT 60 20 184 20

655 ssr-1-159 5 126 60.32 60.0 3.86 CAGGTGATGGTGGTGTGAGG 59.74 55.0 2.94 ATCACGCCACCTAGGTCCTA 60 20 185 20

656 ssr-1-160 1 151 60.04 55.0 3.91 CCTGGGTTCGAATCCTTGCT 59.46 55.0 4.51 GCGTATTTCAGGAGGACCCA 21 20 171 20

657 ssr-1-160 2 152 60.04 55.0 3.91 CCTGGGTTCGAATCCTTGCT 59.46 55.0 4.46 TGCGTATTTCAGGAGGACCC 21 20 172 20

658 ssr-1-160 3 154 60.04 55.0 3.36 CTGCCTGGGTTCGAATCCTT 59.46 55.0 4.51 GCGTATTTCAGGAGGACCCA 18 20 171 20

659 ssr-1-160 4 155 60.04 55.0 3.36 CTGCCTGGGTTCGAATCCTT 59.46 55.0 4.46 TGCGTATTTCAGGAGGACCC 18 20 172 20

660 ssr-1-160 5 120 59.82 60.0 2.67 CAGTGGGAGGGGTAGCATTC 59.46 55.0 4.51 GCGTATTTCAGGAGGACCCA 52 20 171 20

661 ssr-1-161 1 183 59.96 55.0 4.95 TCGCAGTTTAGTTGGACCCC 59.93 47.62 4.02 AGCAGCAAAATGAGAGGACCA 44 20 226 21

662 ssr-1-161 2 182 59.96 55.0 4.79 CGCAGTTTAGTTGGACCCCT 59.93 47.62 4.02 AGCAGCAAAATGAGAGGACCA 45 20 226 21

663 ssr-1-161 3 182 59.96 55.0 4.95 TCGCAGTTTAGTTGGACCCC 58.46 50.0 4.02 GCAGCAAAATGAGAGGACCA 44 20 225 20

664 ssr-1-161 4 181 59.96 55.0 4.79 CGCAGTTTAGTTGGACCCCT 58.46 50.0 4.02 GCAGCAAAATGAGAGGACCA 45 20 225 20

665 ssr-1-161 5 183 59.96 55.0 4.95 TCGCAGTTTAGTTGGACCCC 58.17 50.0 4.46 AGCAGCAAAATGAGAGGACC 44 20 226 20

666 ssr-1-162 1 113 59.96 55.0 3.91 GGCTGGTTTCCTCTCTTGCT 57.35 32.0 3.18 AGGACAAAATAAAAAGACCTGACAA 75 20 187 25

667 ssr-1-162 2 114 59.96 55.0 3.91 GGCTGGTTTCCTCTCTTGCT 57.35 32.0 3.58 AAGGACAAAATAAAAAGACCTGACA 75 20 188 25

668 ssr-1-162 3 114 59.96 55.0 4.01 AGGCTGGTTTCCTCTCTTGC 57.35 32.0 3.18 AGGACAAAATAAAAAGACCTGACAA 74 20 187 25

669 ssr-1-162 4 115 59.96 55.0 4.01 AGGCTGGTTTCCTCTCTTGC 57.35 32.0 3.58 AAGGACAAAATAAAAAGACCTGACA 74 20 188 25

670 ssr-1-162 5 153 60.11 60.0 3.24 GTCTCTCCCCGGGTTAGGAT 57.35 32.0 3.18 AGGACAAAATAAAAAGACCTGACAA 35 20 187 25

671 ssr-1-163 1 178 59.96 55.0 3.36 CCGCTCCTTGTGAGTTTCCT 59.08 47.62 4.58 AGGACAAGGCAGAATAAGGCT 30 20 207 21

672 ssr-1-163 2 179 59.96 55.0 3.36 CCGCTCCTTGTGAGTTTCCT 58.98 52.38 4.35 CAGGACAAGGCAGAATAAGGC 30 20 208 21

673 ssr-1-163 3 180 59.96 55.0 3.36 CCGCTCCTTGTGAGTTTCCT 58.98 52.38 2.69 GCAGGACAAGGCAGAATAAGG 30 20 209 21

674 ssr-1-163 4 183 59.96 55.0 3.36 CCGCTCCTTGTGAGTTTCCT 57.77 45.0 2.4 AATGCAGGACAAGGCAGAAT 30 20 212 20

675 ssr-1-163 5 195 60.39 55.0 5.52 TGTCATCCGTAACACAGCCG 59.08 47.62 4.58 AGGACAAGGCAGAATAAGGCT 13 20 207 21

676 ssr-1-164 1 178 59.96 55.0 3.36 CCGCTCCTTGTGAGTTTCCT 59.08 47.62 4.58 AGGACAAGGCAGAATAAGGCT 6 20 183 21

677 ssr-1-164 2 179 59.96 55.0 3.36 CCGCTCCTTGTGAGTTTCCT 58.98 52.38 4.35 CAGGACAAGGCAGAATAAGGC 6 20 184 21

678 ssr-1-164 3 180 59.96 55.0 3.36 CCGCTCCTTGTGAGTTTCCT 58.98 52.38 2.69 GCAGGACAAGGCAGAATAAGG 6 20 185 21

679 ssr-1-164 4 183 59.96 55.0 3.36 CCGCTCCTTGTGAGTTTCCT 57.77 45.0 2.4 AATGCAGGACAAGGCAGAAT 6 20 188 20

680 ssr-1-164 5 179 59.96 55.0 3.36 CCGCTCCTTGTGAGTTTCCT 60.36 50.0 4.58 CAGGACAAGGCAGAATAAGGCT 6 20 184 22

681 ssr-1-165 1 169 60.32 55.0 3.16 GTCCGAAGCCTTCCACCAAT 59.97 55.0 6.13 TTGCCTATTAGTAGCGCCGG 23 20 191 20

682 ssr-1-165 2 112 60.32 55.0 3.16 GTCCGAAGCCTTCCACCAAT 60.11 55.0 2.71 CCCCCAAATCCGCCTATGTT 23 20 134 20

683 ssr-1-165 3 168 59.6 50.0 2.32 TCCGAAGCCTTCCACCAATT 59.97 55.0 6.13 TTGCCTATTAGTAGCGCCGG 24 20 191 20

684 ssr-1-165 4 111 59.6 50.0 2.32 TCCGAAGCCTTCCACCAATT 60.11 55.0 2.71 CCCCCAAATCCGCCTATGTT 24 20 134 20

685 ssr-1-165 5 166 60.32 55.0 3.16 GTCCGAAGCCTTCCACCAAT 59.75 55.0 4.4 CCTATTAGTAGCGCCGGCAT 23 20 188 20

686 ssr-1-166 1 232 59.3 47.62 4.34 TGGGACGAGAAATGAAACCCT 60.18 60.0 4.35 GCTGACTAGCTGGAGTGACG 20 21 251 20

687 ssr-1-166 2 227 59.3 47.62 4.34 TGGGACGAGAAATGAAACCCT 60.18 60.0 4.35 CTAGCTGGAGTGACGTGTCG 20 21 246 20

688 ssr-1-166 3 246 59.2 52.38 4.79 CCAACACAACACTATGGGACG 60.18 60.0 4.35 GCTGACTAGCTGGAGTGACG 6 21 251 20

689 ssr-1-166 4 241 59.2 52.38 4.79 CCAACACAACACTATGGGACG 60.18 60.0 4.35 CTAGCTGGAGTGACGTGTCG 6 21 246 20

690 ssr-1-166 5 226 59.3 47.62 4.34 TGGGACGAGAAATGAAACCCT 60.6 55.0 4.2 TAGCTGGAGTGACGTGTCGA 20 21 245 20

691 ssr-1-167 1 110 60.18 60.0 3.42 CGACACGTCACTCCAGCTAG 59.69 55.0 4.45 CTGAGTTTGCTTGTGCCTGG 79 20 188 20

692 ssr-1-167 2 111 60.6 55.0 3.32 TCGACACGTCACTCCAGCTA 59.69 55.0 4.45 CTGAGTTTGCTTGTGCCTGG 78 20 188 20

693 ssr-1-167 3 109 59.12 55.0 2.57 GACACGTCACTCCAGCTAGT 59.69 55.0 4.45 CTGAGTTTGCTTGTGCCTGG 80 20 188 20

694 ssr-1-167 4 111 60.18 60.0 3.42 CGACACGTCACTCCAGCTAG 58.97 50.0 4.85 ACTGAGTTTGCTTGTGCCTG 79 20 189 20

695 ssr-1-167 5 105 61.09 55.0 5.03 ATCAATCCACGCCTTGCTCG 59.88 55.0 4.37 AGGAGGAAGTGATGGTGGGA 61 20 165 20

696 ssr-1-168 1 110 58.49 39.13 3.21 AGAATGAAGCAATCTCGACATGT 59.12 50.0 3.41 GACAGACAGACAGAGACAGACA 53 23 162 22

697 ssr-1-168 2 114 58.49 39.13 3.21 AGAATGAAGCAATCTCGACATGT 59.12 50.0 3.41 GACAGACAGACAGACAGAGACA 53 23 166 22

698 ssr-1-168 3 109 58.49 39.13 3.21 AGAATGAAGCAATCTCGACATGT 59.12 50.0 3.51 ACAGACAGACAGAGACAGACAG 53 23 161 22

699 ssr-1-168 4 113 58.49 39.13 3.21 AGAATGAAGCAATCTCGACATGT 59.12 50.0 3.51 ACAGACAGACAGACAGAGACAG 53 23 165 22

700 ssr-1-168 5 109 58.49 39.13 3.21 AGAATGAAGCAATCTCGACATGT 58.06 47.62 3.41 ACAGACAGACAGAGACAGACA 53 23 161 21

701 ssr-1-169 1 100 59.12 50.0 3.51 TGTCTGTCTGTCTCTGTCTGTC 60.1 48.0 3.1 AGAGAGAGAGAGAGAGAGAGAGAGA 61 22 160 25

702 ssr-1-169 2 102 59.12 50.0 3.51 TGTCTGTCTGTCTCTGTCTGTC 60.1 48.0 3.1 AGAGAGAGAGAGAGAGAGAGAGAGA 61 22 162 25

703 ssr-1-169 3 104 59.12 50.0 3.51 TGTCTGTCTGTCTCTGTCTGTC 60.1 48.0 3.1 AGAGAGAGAGAGAGAGAGAGAGAGA 61 22 164 25

704 ssr-1-169 4 106 59.12 50.0 3.51 TGTCTGTCTGTCTCTGTCTGTC 60.1 48.0 3.1 AGAGAGAGAGAGAGAGAGAGAGAGA 61 22 166 25

705 ssr-1-169 5 108 59.12 50.0 3.51 TGTCTGTCTGTCTCTGTCTGTC 60.1 48.0 3.1 AGAGAGAGAGAGAGAGAGAGAGAGA 61 22 168 25

706 ssr-1-172 1 120 59.38 52.38 3.16 ACCCCTGTGCTACACTACTTG 60.03 50.0 4.12 AGCTGAGTAGTGGAATGTTGGG 17 21 136 22

707 ssr-1-172 2 119 59.1 52.38 3.02 CCCCTGTGCTACACTACTTGA 60.03 50.0 4.12 AGCTGAGTAGTGGAATGTTGGG 18 21 136 22

708 ssr-1-172 3 119 59.38 52.38 3.16 ACCCCTGTGCTACACTACTTG 58.64 52.38 4.12 GCTGAGTAGTGGAATGTTGGG 17 21 135 21

709 ssr-1-172 4 120 57.97 50.0 2.24 ACCCCTGTGCTACACTACTT 60.03 50.0 4.12 AGCTGAGTAGTGGAATGTTGGG 17 20 136 22

710 ssr-1-172 5 118 59.1 52.38 3.02 CCCCTGTGCTACACTACTTGA 58.64 52.38 4.12 GCTGAGTAGTGGAATGTTGGG 18 21 135 21

711 ssr-1-176 1 141 60.11 60.0 4.81 GTGCTAATGGAACCCTCCCC 58.73 50.0 4.02 TGGTACTCACAATGCCGGTA 72 20 212 20

712 ssr-1-176 2 141 60.11 60.0 4.81 GTGCTAATGGAACCCTCCCC 59.17 47.62 2.73 TGGTACTCACAATGCCGGTAT 72 20 212 21

713 ssr-1-176 3 141 60.11 60.0 4.81 GTGCTAATGGAACCCTCCCC 58.94 52.63 5.28 TGGTACTCACAATGCCGGT 72 20 212 19

714 ssr-1-176 4 142 59.08 55.0 4.3 AGTGCTAATGGAACCCTCCC 58.73 50.0 4.02 TGGTACTCACAATGCCGGTA 71 20 212 20

715 ssr-1-176 5 140 61.29 60.0 5.4 TGCTAATGGAACCCTCCCCC 58.73 50.0 4.02 TGGTACTCACAATGCCGGTA 73 20 212 20

716 ssr-1-177 1 151 58.99 36.0 2.57 TGCACACAAAATACCTCCTAAATCA 60.25 55.0 3.16 CCACGTGAAACCCCTCCATT 4 25 154 20

717 ssr-1-177 2 148 58.99 36.0 2.57 TGCACACAAAATACCTCCTAAATCA 60.32 55.0 3.85 CGTGAAACCCCTCCATTCGT 4 25 151 20

718 ssr-1-177 3 149 58.99 36.0 2.57 TGCACACAAAATACCTCCTAAATCA 60.32 55.0 3.34 ACGTGAAACCCCTCCATTCG 4 25 152 20

719 ssr-1-177 4 152 59.51 34.62 2.57 TTGCACACAAAATACCTCCTAAATCA 60.25 55.0 3.16 CCACGTGAAACCCCTCCATT 3 26 154 20

720 ssr-1-177 5 150 57.49 37.5 2.57 GCACACAAAATACCTCCTAAATCA 60.25 55.0 3.16 CCACGTGAAACCCCTCCATT 5 24 154 20

721 ssr-1-178 1 174 57.01 40.91 3.71 AGGTACACAAAAATCTGGGAGA 58.32 45.0 3.01 TGCTTCTTTGCTGCAGAACT 7 22 180 20

722 ssr-1-178 2 177 59.93 40.0 3.71 AGAAGGTACACAAAAATCTGGGAGA 58.32 45.0 3.01 TGCTTCTTTGCTGCAGAACT 4 25 180 20

723 ssr-1-178 3 115 59.88 32.0 4.41 ACAACAAACAAAAATTCATGCAGCA 58.32 45.0 3.01 TGCTTCTTTGCTGCAGAACT 66 25 180 20

724 ssr-1-178 4 177 57.01 40.91 3.71 AGGTACACAAAAATCTGGGAGA 58.03 45.0 4.26 TCTTGCTTCTTTGCTGCAGA 7 22 183 20

725 ssr-1-178 5 176 58.69 41.67 3.71 GAAGGTACACAAAAATCTGGGAGA 58.32 45.0 3.01 TGCTTCTTTGCTGCAGAACT 5 24 180 20

726 ssr-1-180 1 156 59.83 55.0 4.17 GTGTGTGTGTGTGTGTGTGG 57.01 32.0 1.94 AGATTAACGGTAGCCTTAAGTTTTT 10 20 165 25

727 ssr-1-180 2 158 59.83 55.0 4.17 GTGTGTGTGTGTGTGTGTGG 57.52 34.62 2.43 GTAGATTAACGGTAGCCTTAAGTTTT 10 20 167 26

728 ssr-1-180 3 157 59.13 50.0 3.82 TGTGTGTGTGTGTGTGTGTG 57.01 32.0 1.94 AGATTAACGGTAGCCTTAAGTTTTT 9 20 165 25

729 ssr-1-180 4 159 59.13 50.0 3.82 TGTGTGTGTGTGTGTGTGTG 57.01 32.0 1.94 AGATTAACGGTAGCCTTAAGTTTTT 7 20 165 25

730 ssr-1-180 5 161 59.13 50.0 3.82 TGTGTGTGTGTGTGTGTGTG 57.01 32.0 1.94 AGATTAACGGTAGCCTTAAGTTTTT 5 20 165 25

731 ssr-1-181 1 197 59.89 55.0 2.01 GGCATCGCCCCATAGGTAAA 59.96 55.0 5.01 TCCTGGTGAAAGGATGGTGC 9 20 205 20

732 ssr-1-181 2 196 59.89 55.0 2.01 GGCATCGCCCCATAGGTAAA 59.96 55.0 4.4 CCTGGTGAAAGGATGGTGCT 9 20 204 20

733 ssr-1-181 3 228 59.89 55.0 2.01 GGCATCGCCCCATAGGTAAA 59.76 55.0 5.69 GTGTGTATTGTGGTGTGCGG 9 20 236 20

734 ssr-1-181 4 194 59.89 55.0 2.01 GGCATCGCCCCATAGGTAAA 60.25 55.0 4.85 TGGTGAAAGGATGGTGCTGG 9 20 202 20

735 ssr-1-181 5 192 59.89 55.0 2.01 GGCATCGCCCCATAGGTAAA 60.25 55.0 4.51 GTGAAAGGATGGTGCTGGGT 9 20 200 20

736 ssr-1-182 1 167 59.47 36.0 3.91 GCCAATAAATGCCACATAATTTGCT 59.41 55.0 4.26 CCTGTAGATTGCGCATGAGC 32 25 198 20

737 ssr-1-182 2 168 59.47 36.0 3.68 AGCCAATAAATGCCACATAATTTGC 59.41 55.0 4.26 CCTGTAGATTGCGCATGAGC 31 25 198 20

738 ssr-1-182 3 171 57.33 34.78 1.9 ACAAGCCAATAAATGCCACATAA 59.41 55.0 4.26 CCTGTAGATTGCGCATGAGC 28 23 198 20

739 ssr-1-182 4 173 57.33 34.78 3.21 TTACAAGCCAATAAATGCCACAT 59.41 55.0 4.26 CCTGTAGATTGCGCATGAGC 26 23 198 20

740 ssr-1-182 5 174 57.33 34.78 4.17 ATTACAAGCCAATAAATGCCACA 59.41 55.0 4.26 CCTGTAGATTGCGCATGAGC 25 23 198 20

741 ssr-1-183 1 181 57.06 50.0 4.18 CCTTAGTAAGGCGAACGTCT 59.04 43.48 3.1 TGTGTGAGGTTCTTGAAGAGAGA 74 20 254 23

742 ssr-1-183 2 181 57.06 50.0 4.18 CCTTAGTAAGGCGAACGTCT 59.96 45.83 3.2 TGTGTGAGGTTCTTGAAGAGAGAG 74 20 254 24

743 ssr-1-183 3 180 57.06 50.0 4.18 CCTTAGTAAGGCGAACGTCT 59.72 45.83 3.1 GTGTGAGGTTCTTGAAGAGAGAGA 74 20 253 24

744 ssr-1-183 4 181 57.06 50.0 4.18 CCTTAGTAAGGCGAACGTCT 57.67 45.45 3.2 TGTGTGAGGTTCTTGAAGAGAG 74 20 254 22

745 ssr-1-183 5 181 59.62 47.83 2.57 CCTTAGTAAGGCGAACGTCTAGT 59.04 43.48 3.1 TGTGTGAGGTTCTTGAAGAGAGA 74 23 254 23

746 ssr-1-184 1 196 57.06 50.0 4.18 CCTTAGTAAGGCGAACGTCT 58.98 40.91 3.58 AGCACTTCCATGTTTTGTGTGA 10 20 205 22

747 ssr-1-184 2 196 57.06 50.0 4.18 CCTTAGTAAGGCGAACGTCT 59.93 43.48 3.51 AGCACTTCCATGTTTTGTGTGAG 10 20 205 23

748 ssr-1-184 3 195 57.06 50.0 4.18 CCTTAGTAAGGCGAACGTCT 58.63 45.45 3.51 GCACTTCCATGTTTTGTGTGAG 10 20 204 22

749 ssr-1-184 4 194 57.06 50.0 4.18 CCTTAGTAAGGCGAACGTCT 59.62 43.48 3.85 CACTTCCATGTTTTGTGTGAGGT 10 20 203 23

750 ssr-1-184 5 195 57.06 50.0 4.18 CCTTAGTAAGGCGAACGTCT 57.56 42.86 3.58 GCACTTCCATGTTTTGTGTGA 10 20 204 21

751 ssr-1-185 1 173 59.39 50.0 4.0 TATTTCAGCGGCACTCACCT 57.83 41.67 3.5 TCCTAACCTAACCTAACCTAACCT 13 20 185 24

752 ssr-1-185 2 165 60.88 55.0 3.85 CGGCACTCACCTGAAACGAA 57.83 41.67 3.5 TCCTAACCTAACCTAACCTAACCT 21 20 185 24

753 ssr-1-185 3 172 60.96 55.0 4.0 ATTTCAGCGGCACTCACCTG 57.83 41.67 3.5 TCCTAACCTAACCTAACCTAACCT 14 20 185 24

754 ssr-1-185 4 174 59.39 50.0 4.0 TATTTCAGCGGCACTCACCT 58.43 40.0 3.5 TTCCTAACCTAACCTAACCTAACCT 13 20 186 25

755 ssr-1-185 5 174 59.79 47.62 4.0 ATATTTCAGCGGCACTCACCT 57.83 41.67 3.5 TCCTAACCTAACCTAACCTAACCT 12 21 185 24

756 ssr-1-186 1 137 58.08 41.67 3.08 AGGTTAGGTTAGGTTAGGTTAGGT 59.66 45.83 3.97 GGCCAGTTAAAGATGGTTATTCCC 47 24 183 24

757 ssr-1-186 2 142 58.08 41.67 3.08 AGGTTAGGTTAGGTTAGGTTAGGT 59.66 45.83 3.97 GGCCAGTTAAAGATGGTTATTCCC 42 24 183 24

758 ssr-1-186 3 137 58.67 40.0 3.5 AGGTTAGGTTAGGTTAGGTTAGGTT 59.66 45.83 3.97 GGCCAGTTAAAGATGGTTATTCCC 47 25 183 24

759 ssr-1-186 4 142 58.67 40.0 3.5 AGGTTAGGTTAGGTTAGGTTAGGTT 59.66 45.83 3.97 GGCCAGTTAAAGATGGTTATTCCC 42 25 183 24

760 ssr-1-186 5 138 58.08 41.67 3.08 AGGTTAGGTTAGGTTAGGTTAGGT 59.05 41.67 3.01 TGGCCAGTTAAAGATGGTTATTCC 47 24 184 24

761 ssr-1-187 1 134 59.2 55.0 3.58 GTTCTGGAACTTGGCGTAGC 58.42 40.91 2.52 AGGGTAAATGGCACCAAAAAGA 60 20 193 22

762 ssr-1-187 2 134 59.2 55.0 3.58 GTTCTGGAACTTGGCGTAGC 60.02 41.67 2.9 AGGGTAAATGGCACCAAAAAGATG 60 20 193 24

763 ssr-1-187 3 133 59.2 55.0 3.58 GTTCTGGAACTTGGCGTAGC 60.02 41.67 2.92 GGGTAAATGGCACCAAAAAGATGA 60 20 192 24

764 ssr-1-187 4 134 59.2 55.0 3.58 GTTCTGGAACTTGGCGTAGC 58.83 39.13 2.4 AGGGTAAATGGCACCAAAAAGAT 60 20 193 23

765 ssr-1-187 5 133 59.2 55.0 3.58 GTTCTGGAACTTGGCGTAGC 58.75 43.48 2.9 GGGTAAATGGCACCAAAAAGATG 60 20 192 23

766 ssr-1-188 1 205 60.25 55.0 2.17 GCGTCGCTCCATAGGGAAAT 59.82 50.0 3.32 TCATGGTCAACGTGGGTGTT 3 20 207 20

767 ssr-1-188 2 206 60.25 55.0 2.17 GCGTCGCTCCATAGGGAAAT 59.82 50.0 4.16 TTCATGGTCAACGTGGGTGT 3 20 208 20

768 ssr-1-188 3 173 60.25 55.0 2.17 GCGTCGCTCCATAGGGAAAT 60.39 60.0 3.67 GTGATGGTCGTCCTGGTGAC 3 20 175 20

769 ssr-1-188 4 156 60.25 55.0 2.17 GCGTCGCTCCATAGGGAAAT 60.68 60.0 3.62 GACAGGATGGTGCTGGGTTC 3 20 158 20

770 ssr-1-188 5 172 60.25 55.0 2.17 GCGTCGCTCCATAGGGAAAT 60.83 55.0 3.58 TGATGGTCGTCCTGGTGACA 3 20 174 20

771 ssr-1-190 1 128 58.32 42.86 3.16 TGGAATTTGGCAGTTCCTTGT 59.82 50.0 5.68 TGAATTCATGATCCGGGCGT 78 21 205 20

772 ssr-1-190 2 128 58.96 40.91 2.83 TGGAATTTGGCAGTTCCTTGTT 59.82 50.0 5.68 TGAATTCATGATCCGGGCGT 78 22 205 20

773 ssr-1-190 3 129 58.96 40.91 3.16 TTGGAATTTGGCAGTTCCTTGT 59.82 50.0 5.68 TGAATTCATGATCCGGGCGT 77 22 205 20

774 ssr-1-190 4 125 58.32 42.86 3.16 TGGAATTTGGCAGTTCCTTGT 60.82 55.0 3.77 ATTCATGATCCGGGCGTTGG 78 21 202 20

775 ssr-1-190 5 129 58.32 42.86 3.16 TGGAATTTGGCAGTTCCTTGT 59.13 55.0 6.13 CTGAATTCATGATCCGGGCG 78 21 206 20

776 ssr-1-193 1 100 59.23 47.62 4.02 TCTGAGCAAGGAGTTTGGTGA 59.54 50.0 4.83 TGCTCAGTTGTCTTCACCGT 58 21 157 20

777 ssr-1-193 2 112 59.23 47.62 3.27 TGAGCAAGGAGTTTGGTGAGA 59.47 50.0 4.26 TAGCTCGCTTGTCATGCTCA 60 21 171 20

778 ssr-1-193 3 114 59.23 47.62 4.02 TCTGAGCAAGGAGTTTGGTGA 59.47 50.0 4.26 TAGCTCGCTTGTCATGCTCA 58 21 171 20

779 ssr-1-193 4 114 59.23 47.62 3.27 TGAGCAAGGAGTTTGGTGAGA 59.47 50.0 3.79 AGTAGCTCGCTTGTCATGCT 60 21 173 20

780 ssr-1-193 5 116 59.23 47.62 4.02 TCTGAGCAAGGAGTTTGGTGA 59.47 50.0 3.79 AGTAGCTCGCTTGTCATGCT 58 21 173 20

781 ssr-1-194 1 145 60.16 50.0 3.71 AGCGAGCTACTAATCTGGAGGA 60.03 60.0 4.51 GATAGTAGGGTGGGGTGGGT 0 22 144 20

782 ssr-1-194 2 146 60.16 50.0 3.71 AGCGAGCTACTAATCTGGAGGA 60.03 60.0 4.61 TGATAGTAGGGTGGGGTGGG 0 22 145 20

783 ssr-1-194 3 144 59.77 54.55 3.69 GCGAGCTACTAATCTGGAGGAG 60.03 60.0 4.51 GATAGTAGGGTGGGGTGGGT 1 22 144 20

784 ssr-1-194 4 145 59.77 54.55 3.69 GCGAGCTACTAATCTGGAGGAG 60.03 60.0 4.61 TGATAGTAGGGTGGGGTGGG 1 22 145 20

785 ssr-1-194 5 144 58.77 52.38 3.71 GCGAGCTACTAATCTGGAGGA 60.03 60.0 4.51 GATAGTAGGGTGGGGTGGGT 1 21 144 20

786 ssr-1-195 1 132 57.43 50.0 3.85 TCTCTGACCTCTGTCTTCGT 60.04 60.0 3.14 GTACGGGATGAGGGCTATGC 47 20 178 20

787 ssr-1-195 2 131 57.43 50.0 3.85 TCTCTGACCTCTGTCTTCGT 60.18 55.0 3.79 TACGGGATGAGGGCTATGCT 47 20 177 20

788 ssr-1-195 3 132 58.13 47.62 3.85 TCTCTGACCTCTGTCTTCGTT 60.04 60.0 3.14 GTACGGGATGAGGGCTATGC 47 21 178 20

789 ssr-1-195 4 127 57.43 50.0 3.85 TCTCTGACCTCTGTCTTCGT 59.61 55.0 3.85 GGATGAGGGCTATGCTCGTT 47 20 173 20

790 ssr-1-195 5 131 58.13 47.62 3.85 TCTCTGACCTCTGTCTTCGTT 60.18 55.0 3.79 TACGGGATGAGGGCTATGCT 47 21 177 20

791 ssr-1-196 1 127 60.25 55.0 3.53 CCACGAGCTGACTGTTGGAA 59.89 50.0 2.83 TTCGTTGGCGGAGGTAACAA 19 20 145 20

792 ssr-1-196 2 131 60.25 55.0 3.16 GGAACCACGAGCTGACTGTT 59.89 50.0 2.83 TTCGTTGGCGGAGGTAACAA 15 20 145 20

793 ssr-1-196 3 128 60.25 55.0 3.53 CCACGAGCTGACTGTTGGAA 60.32 55.0 2.41 CTTCGTTGGCGGAGGTAACA 19 20 146 20

794 ssr-1-196 4 131 60.25 55.0 3.53 CCACGAGCTGACTGTTGGAA 60.32 55.0 3.08 CAACTTCGTTGGCGGAGGTA 19 20 149 20

795 ssr-1-196 5 132 60.25 55.0 3.16 GGAACCACGAGCTGACTGTT 60.32 55.0 2.41 CTTCGTTGGCGGAGGTAACA 15 20 146 20

796 ssr-1-198 1 151 59.13 50.0 3.82 TGTGTGTGTGTGTGTGTGTG 59.96 50.0 4.26 ACCAAATGATGCCCAGCTCA 1 20 151 20

797 ssr-1-198 2 150 59.13 50.0 3.72 GTGTGTGTGTGTGTGTGTGT 59.96 50.0 4.26 ACCAAATGATGCCCAGCTCA 2 20 151 20

798 ssr-1-198 3 152 59.13 50.0 3.72 GTGTGTGTGTGTGTGTGTGT 59.96 50.0 4.26 ACCAAATGATGCCCAGCTCA 0 20 151 20

799 ssr-1-198 4 153 59.13 50.0 3.82 TGTGTGTGTGTGTGTGTGTG 59.67 50.0 4.24 AGACCAAATGATGCCCAGCT 1 20 153 20

800 ssr-1-198 5 152 59.13 50.0 3.72 GTGTGTGTGTGTGTGTGTGT 59.67 50.0 4.24 AGACCAAATGATGCCCAGCT 2 20 153 20

801 ssr-1-199 1 193 60.11 60.0 4.95 GGCATCTAGACCAACACCCC 59.42 47.83 3.58 AGAGAGAGAGAGAGACTGTCACA 14 20 206 23

802 ssr-1-199 2 163 60.11 60.0 4.95 GGCATCTAGACCAACACCCC 57.41 42.86 3.86 TGAAAGGAAAAAGGCTCAGGA 14 20 176 21

803 ssr-1-199 3 191 59.82 60.0 5.73 CATCTAGACCAACACCCCGG 59.42 47.83 3.58 AGAGAGAGAGAGAGACTGTCACA 16 20 206 23

804 ssr-1-199 4 161 59.82 60.0 5.73 CATCTAGACCAACACCCCGG 57.41 42.86 3.86 TGAAAGGAAAAAGGCTCAGGA 16 20 176 21

805 ssr-1-199 5 190 60.25 55.0 5.28 ATCTAGACCAACACCCCGGT 59.42 47.83 3.58 AGAGAGAGAGAGAGACTGTCACA 17 20 206 23

806 ssr-1-200 1 137 57.41 42.86 2.69 TCCTGAGCCTTTTTCCTTTCA 59.63 55.0 1.75 GTTGGTCGGCGTTCGTAATC 64 21 200 20

807 ssr-1-200 2 130 57.41 42.86 2.69 TCCTGAGCCTTTTTCCTTTCA 59.62 55.0 2.59 GGCGTTCGTAATCCCGACTA 64 21 193 20

808 ssr-1-200 3 144 57.41 42.86 2.69 TCCTGAGCCTTTTTCCTTTCA 60.39 55.0 4.84 GGAATAGGTTGGTCGGCGTT 64 21 207 20

809 ssr-1-200 4 148 57.41 42.86 2.69 TCCTGAGCCTTTTTCCTTTCA 59.38 55.0 4.79 TGGTGGAATAGGTTGGTCGG 64 21 211 20

810 ssr-1-200 5 137 58.09 40.91 2.69 TCCTGAGCCTTTTTCCTTTCAA 59.63 55.0 1.75 GTTGGTCGGCGTTCGTAATC 64 22 200 20
